# Organophosphorus-catalyzed relay oxidation of H-Bpin: electrophilic C–H borylation of heteroarenes†

**DOI:** 10.1039/d0sc05620k

**Published:** 2020-11-19

**Authors:** Jeffrey M. Lipshultz, Yue Fu, Peng Liu, Alexander T. Radosevich

**Affiliations:** Department of Chemistry, Massachusetts Institute of Technology 77 Massachusetts Avenue Cambridge MA 02139 USA radosevich@mit.edu; Department of Chemistry, University of Pittsburgh 219 Parkman Avenue Pittsburgh PA 15260 USA pengliu@pitt.edu

## Abstract

A nontrigonal phosphorus triamide (1, P{N[*o*-NMe-C_6_H_4_]_2_}) is shown to catalyze C–H borylation of electron-rich heteroarenes with pinacolborane (HBpin) in the presence of a mild chloroalkane reagent. C–H borylation proceeds for a range of electron-rich heterocycles including pyrroles, indoles, and thiophenes of varied substitution. Mechanistic studies implicate an initial P–N cooperative activation of HBpin by 1 to give *P*-hydrido diazaphospholene 2, which is diverted by Atherton–Todd oxidation with chloroalkane to generate *P*-chloro diazaphospholene 3. DFT calculations suggest subsequent oxidation of pinacolborane by 3 generates chloropinacolborane (ClBpin) as a transient electrophilic borylating species, consistent with observed substituent effects and regiochemical outcomes. These results illustrate the targeted diversion of established reaction pathways in organophosphorus catalysis to enable a new mode of main group-catalyzed C–H borylation.

## Abstract 2

A nontrigonal phosphorus triamide (1, P{N[*o*-NMe-C_6_H_4_]_2_}) is shown to catalyze C–H borylation of electron-rich heteroarenes with pinacolborane (HBpin) in the presence of a mild chloroalkane reagent.

## Graphical abstract

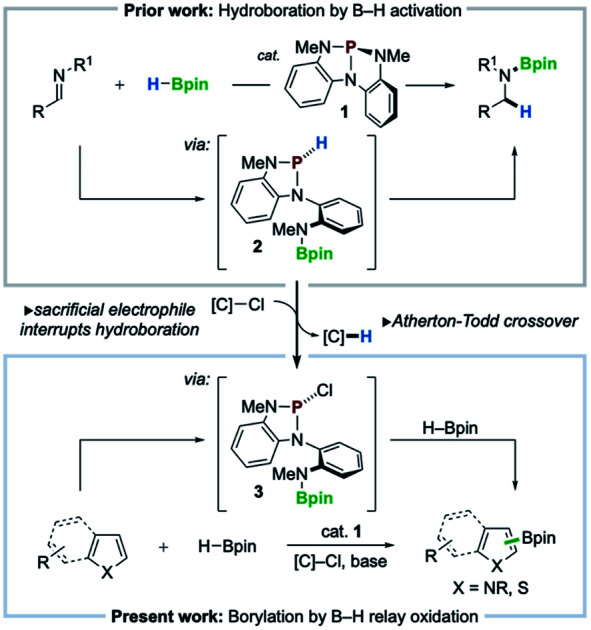

## Introduction

**Scheme 1:**
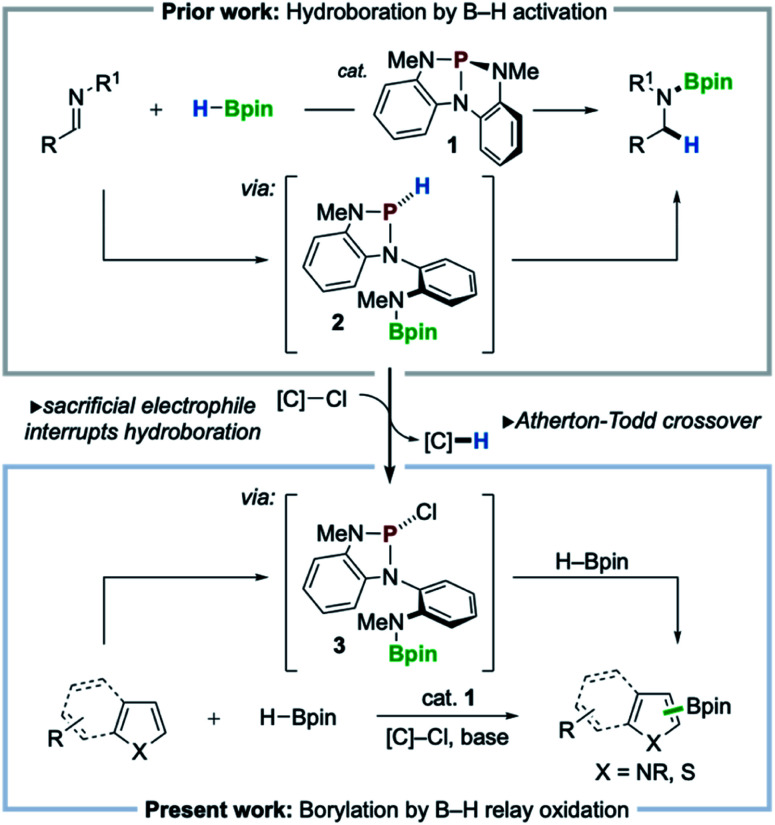
Previous work: imine hydroboration catalyzed by nontrigonal phosphorus triamide 1. Guiding hypothesis: diversion of reactivity *via* relay oxidation can be accomplished by Atherton–Todd crossover of P–H in 2 for P–Cl in 3. Present work: direct heteroarene C–H borylation with HBpin catalyzed by 1 in presence of chloroalkane electrophile.

Recent innovations in synthetic organophosphorus chemistry are fueling new opportunities for catalysis.^1^ As a complement to well-known nucleophilic (Lewis basic) reactivity,^2^ new structural design principles are emerging that now enable organophosphorus catalysis to comprise Lewis acidic,^3^ dehydrative,^4^ redox O-atom transfer,^5^ and reductive^6^ activation modes for catalysis. Within this vein, nontrigonal phosphorus triamide 1 (ref. 7) was reported to catalyze the activation and transfer of H–Bpin to imines in a ligand cooperative^8^ fashion *via* the intermediacy of *P*-hydrido diazaphospholene 2 (Scheme 1, top).^9^ The pronounced hydricity of related *P*-hydrido diazaphospholenes—studied extensively by Gudat^10^— has been advanced by Kinjo,^11^ Speed,^12^ Cramer,^13^ and Melen^14^ within the context of hydroboration catalysis to effect either 1,2- or 1,4-addition of H–Bpin to π-electrophiles (*e.g.* imines, carbonyls, and pyridines).

With a view toward enabling new reactivity, we considered that catalytic hydroboration by 1 might be interrupted by the inclusion of an exogenous sacrificial electrophile to scavenge the activated hydride of 2, achieving catalytic oxidative crossover to *P*-chloro diazaphospholene 3. Doing so would present the possibility that the established reductive manifold, *i.e.* hydroboration with H–Bpin, of diazaphospholene catalysis might be diverted *via* sequential oxidative transfers, or relay oxidation, to access electrophilic “pinB^+^” synthons^15^ for delivery to suitable nucleophilic substrates for C–H borylation (Scheme 1, bottom). Herein, we realize this vision and achieve a C–H borylation^16^ of electron-rich heteroarenes with H–Bpin as the boron donor under the catalytic action of phosphorus triamide 1 and a mild chloroalkane oxidant, establishing a new organophosphorus catalyzed platform for C–H borylation.

## Results and discussion

The success of the proposed catalytic platform is predicated upon the compatibility and interplay of both reducing (HBpin) and oxidizing (sacrificial electrophile) reagents with the organophosphorus catalyst 1. Inspired by an observed Atherton–Todd-like^17^ chlorination of *P*-hydrido diazaphospholene 2,^9^ we considered the possibility that even a weak electrophilic reagent such as chloroform might efficiently serve to capture the P–H hydride of 2 and thus divert H–Bpin activation toward electrophilic borylation *via* relay oxidation. As an initial probe of this hypothesis, the reaction of *N*-Me-pyrrole (4a) with HBpin (1 equiv.) and NEt_3_ under the action of catalytic phosphorus triamide 1 (10 mol%) in chloroform at 80 °C was attempted. In the event, C–H borylation of 4a was indeed observed, giving C2-functionalized product 5a in 8% yield (Table 1, entry 1). Use of CHCl_3_ in reagent quantities in MeCN proved equally effective (entry 2), and increasing the reaction temperature to 100 °C resulted in improved 29% yield (entry 3). Although cationic borenium reagents are known to produce a mixture of borylation regioisomers,^15*b*^ the C3-functionalized isomer was not detected under these catalytic conditions. When bromoform was used in place of chloroform over a range of temperatures, no borylated product was observed (entry 4), and the use of exogenous bromide with CHCl_3_ saw no improvement in yield (28% yield, entry 5). An evaluation of organic and inorganic bases determined that Hünig's base was optimal, providing 35% yield (entry 6), while optimization of reagent and catalyst loading resulted in further improvement to 50% yield (entry 7). Replacement of CHCl_3_ with a higher-boiling chloroalkane (α,α-dichlorotoluene,^18^ entries 9 and 10) improved the efficiency to 60% yield. Use of catecholborane in place of pinacolborane resulted in no borylation, as rapid decomposition of catalyst 1 was observed (entry 11). Control experiments establish that catalyst 1, base, and chloroalkane are each required for C–B bond formation (entries 12–14). Thus, phosphorus triamide 1 is indeed responsible for catalyzing this C–H borylation reaction, establishing new precedent for organophosphorus catalysis of this valuable transformation as a complement to established transition metal-^8*b*,19,29^ and organoboron-catalyzed^20^ methods.

**Table 1:** Discovery and optimization of organophosphorus-catalyzed C–H borylation of electron-rich heterocycles^a^

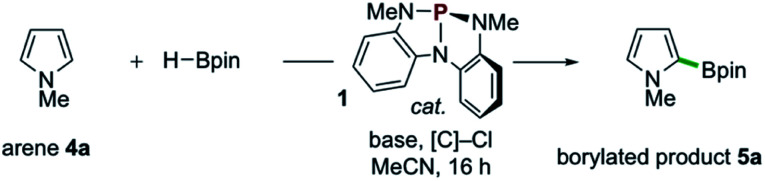						
Entry	1 (mol%)	HBpin (equiv.)	Base (equiv.)	[C]–Cl (equiv.)	Temp (°C)	5a^b^ (%)
1	10	1	NEt_3_ (1)	CHCl_3_^c^	80	8
2	10	1	NEt_3_ (1)	CHCl_3_ (2)	80	8
3	10	1	NEt_3_ (1)	CHCl_3_ (2)	100	29
4	10	1	NEt_3_ (1)	CHBr_3_ (2)	100^d^	0
5	10	1	NEt_3_ (1)	CHCl_3_ (2)^e^	100	28
6	10	1	EtN^i^Pr_2_ (1)	CHCl_3_ (2)	100	35
8	20	2	EtN^i^Pr_2_ (2)	CHCl_3_ (2)	100	50
9	20	2	EtN^i^Pr_2_ (2)	PhCHCl_2_ (2)	100	60
10	20	2	EtN^i^Pr_2_ (2)	PhCHCl_2_ (1)	100	60
11	20	0^f^	EtN^i^Pr_2_ (2)	PhCHCl_2_ (1)	100	0
12	0	2	EtN^i^Pr_2_ (2)	PhCHCl_2_ (1)	100	0
13	20	2	0	PhCHCl_2_ (1)	100	0
14	20	2	EtN^i^Pr_2_ (2)	None	100	0

a Reactions conducted on 0.125 mmol scale, 0.25 M in MeCN.

b ^1^H NMR yields compared to internal standard.

c CHCl_3_ used as solvent in place of MeCN.

d Reactions conducted at 25, 50, and 80 °C also yielded 0% 5a.

e Et_4_NBr (0.1 equiv.) additive.

f HBcat (2 equiv.) used in place of HBpin. HBpin = pinacolborane. HBcat = catecholborane.

**Scheme 2:**
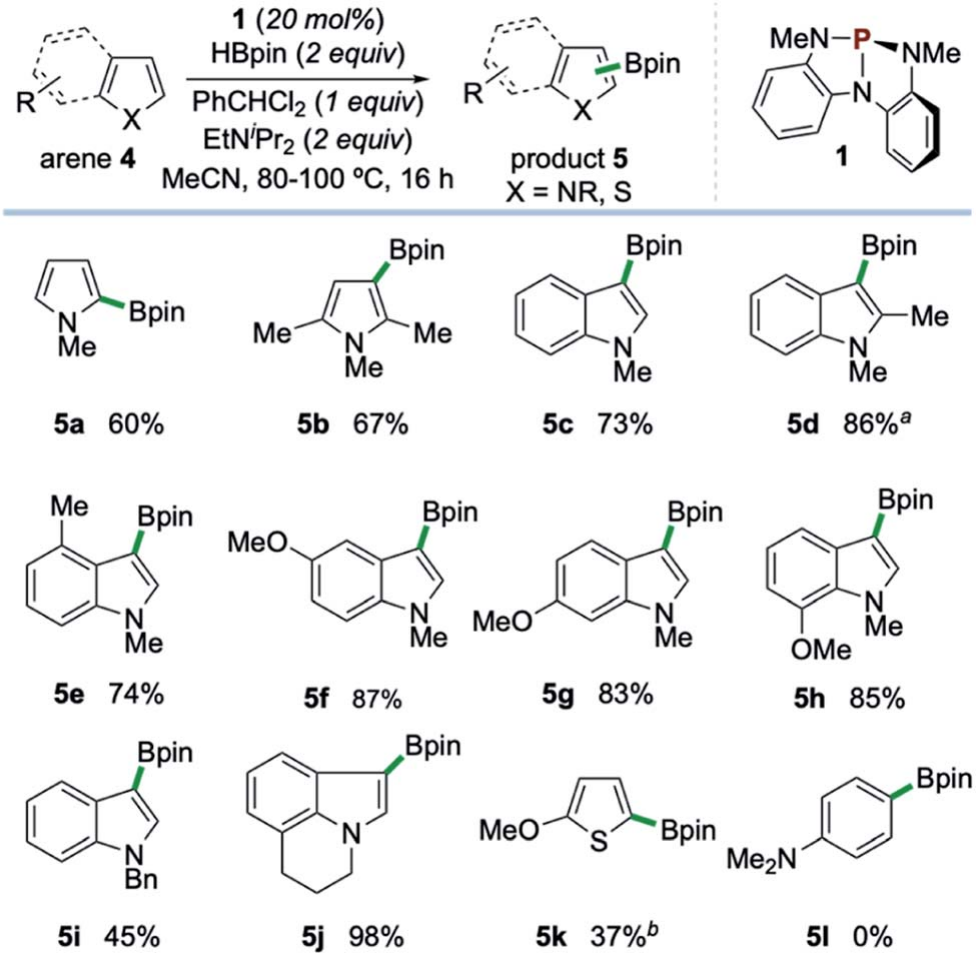
Synthetic scope of organophosphorus-catalyzed C–H borylation of electron-rich heterocycles. All yields isolated from 0.5 mmol scale reactions conducted for 16 hours. See ESI† for full synthetic details. ^*a*^Reaction conducted at 60 °C. ^*b*^Reaction conducted with 2 equivalents of 2-methoxythiophene (4k), yield based on HBpin as limiting reagent. See ESI.†

Examples of the C–H borylation of electron-rich heteroarenes illustrating the scope and limitations of this phosphacatalytic method are shown in Scheme 2. While the borylation of *N*-Me-pyrrole (4a) provided exclusively C2-borylated product 5a in 60% yield, borylation is efficiently diverted to the C3 position by blocking both the C2- and C5-positions with methyl groups as in 5b (67% yield at 80 °C). When the same conditions were applied to *N*-Me-indole, similarly efficient C3-borylation was achieved, delivering product 5c as the sole regioisomer in 73% yield. Substitution on the 5-membered ring, as in 1,2-dimethylindole, led to increased reactivity at the sterically encumbered C3-position even at lower temperature (5d, 86% yield, 60 °C), indicating an overwhelming electronic bias relative to steric effects. Substitution at all positions of the benzenoid ring could be similarly tolerated, as methyl substitution at the C4-position had no effect on the efficiency with respect to unsubstituted indole substrate, yielding 5e in 74% yield. Substitution at each of the C5-, C6-, and C7-positions with a methoxy group boosted efficiency, delivering C3-borylated product in excellent yield (5f–5h, 83–87% yields). While *N*-H and *N*-silyl indoles did not deliver synthetically useful yield of borylation products, *N*-Bn-indole could be borylated in good efficiency (5i, 45% yield, >99% yield based on recovered starting material). Also, tricyclic indole alkaloid lilolidine could be borylated in near-quantitative efficiency to deliver 5j (98% yield). Other electron-rich heterocycles are subject to borylation, as demonstrated by the formation of 5k from 2-OMe-thiophene. However, π-rich benzenes are unreactive, as exemplified by no formation of 5l from *N*,*N*-dimethylaniline.^21^

**Scheme 3:**
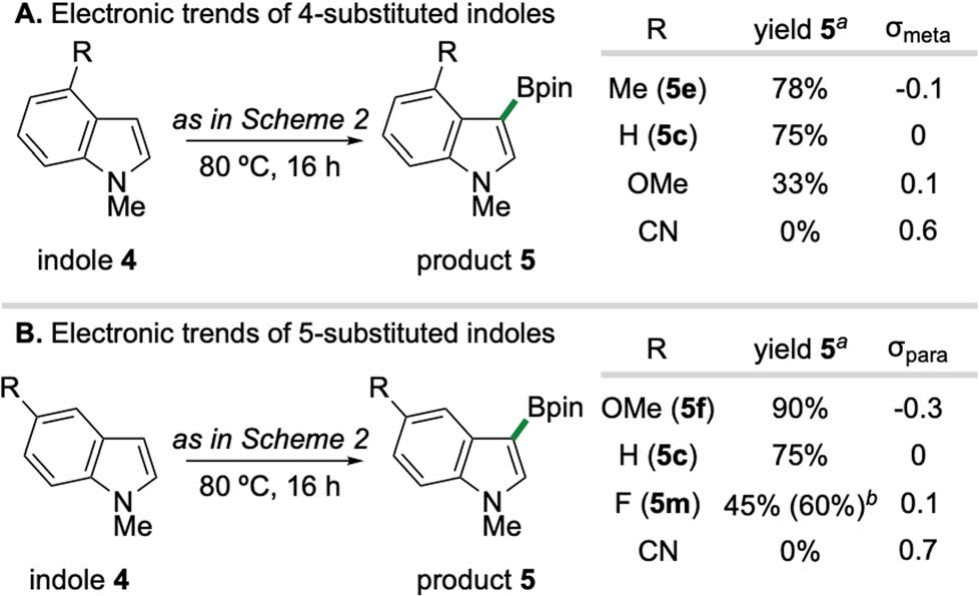
Electronic trends of indole substitution. ^*a*1^H NMR yields compared to internal standard for 0.125 mmol scale reactions conducted for 16 hours. ^*b*^Isolated yield of product 5m from 0.5 mmol scale reaction at 100 °C for 24 hours.

A systematic variation of substituents on the benzenoid ring of indole substrates revealed a reactivity pattern which is gated by heteroarene nucleophilicity (Scheme 3),^22^ indicative of a borylation event proceeding *via* an electrophilic aromatic substitution reaction (S_E_Ar) pathway.^23^ In the case of C4- substituted indoles (Scheme 3A), the formation of borylation products 5 correlates with Hammett substituent constant *σ*_*meta*_; specifically, inclusion of a 4-methoxy substituent (*σ*_*meta*_ = 0.1) leads to lower yield (33%) than the parent 5-unsubstituted substrate (75%). Relatedly, C5-substitution of indoles trend with substituent constant *σ*_para_ (Scheme 3B), such that 5-methoxy substitution (*σ*_*para*_ = −0.3) gives higher yield (90%) than the parent 4-unsubstituted substrate.^24^ While strongly electron-withdrawing substituents such as CN completely suppressed borylation independent of C4/C5 position on the indole, weakly withdrawing 5-F substitution on 1-methylindole provided synthetically useful levels of efficiency upon conducting the reaction at 100 °C for 24 hours (5m, 60% yield).

**Scheme 4:**
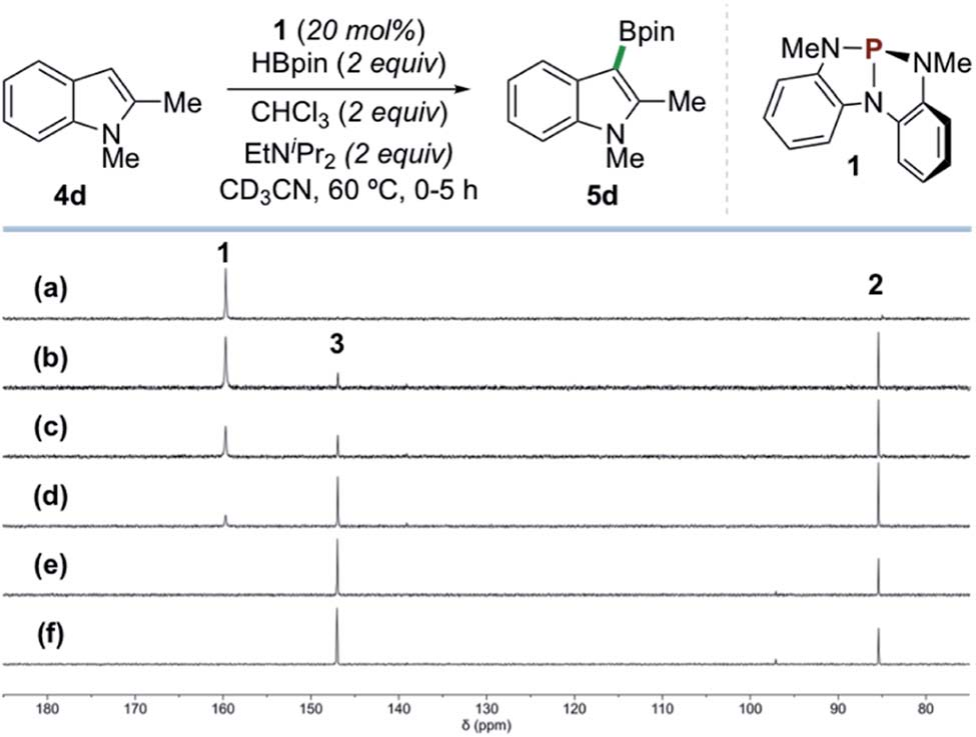
Time-stacked *in situ*^31^P NMR spectra of the borylation of 4d by HBpin as in Scheme 2 (CD_3_CN, 202 MHz, 60 °C) at the following time points: (a) 0 min, (b) 15 min, (c) 30 min, (d) 1 h, (e) 3 h, and (f) 5 h, showing 1 (*δ* 159.7 ppm), 2 (*δ* 85.4 ppm), and 3 (*δ* 147.0 ppm).

*In situ* NMR analysis of a representative catalytic reaction is consistent with the evolution of compounds 1, 2, and 3 as envisioned (Scheme 4). Specifically, ^31^P{^1^H} NMR spectral monitoring of the catalytic borylation of 1,2-dimethylindole (4d) with HBpin and CHCl_3_ by catalyst 1 shows initial conversion of 1 (*δ* 159.7 ppm) to *P*-hydrido diazaphospholene 2 (*δ* 85.4 ppm) by reaction with HBpin. Compound 2 is further converted to *P*-chloro diazaphospholene 3 (*δ* 147.0 ppm) within *ca.* 2 h, ultimately reaching a steady state ratio of 3 : 2 (*ca.* 4 : 1) that persists for the duration of the borylation reaction (96% yield of 5d after 16 h). Complementary monitoring in the ^1^H and ^11^B NMR channels indicates a delay in formation of borylation product 5d until a significant concentration of 3 is accrued (2% yield of 5d at 2 h). Evidently, *P*-chloro diazaphospholene 3 is necessary for product formation; indeed, when 3 is employed directly as precatalyst under otherwise identical conditions, formation of product 5d is observed without an induction period. Moreover, ^31^P NMR spectra confirm the formation of 2 under these catalytic reaction conditions with precatalyst 3, converging on a 4 : 1 steady state ratio of 3 : 2 as was observed by reaction with precatalyst 1. Taken together, these spectroscopic results are consistent with sequential activation of HBpin by 1 and of CHCl_3_ by 2, followed by turnover-limiting reaction of 3, presumably to effect C–H borylation of 4d.

**Scheme 5:**
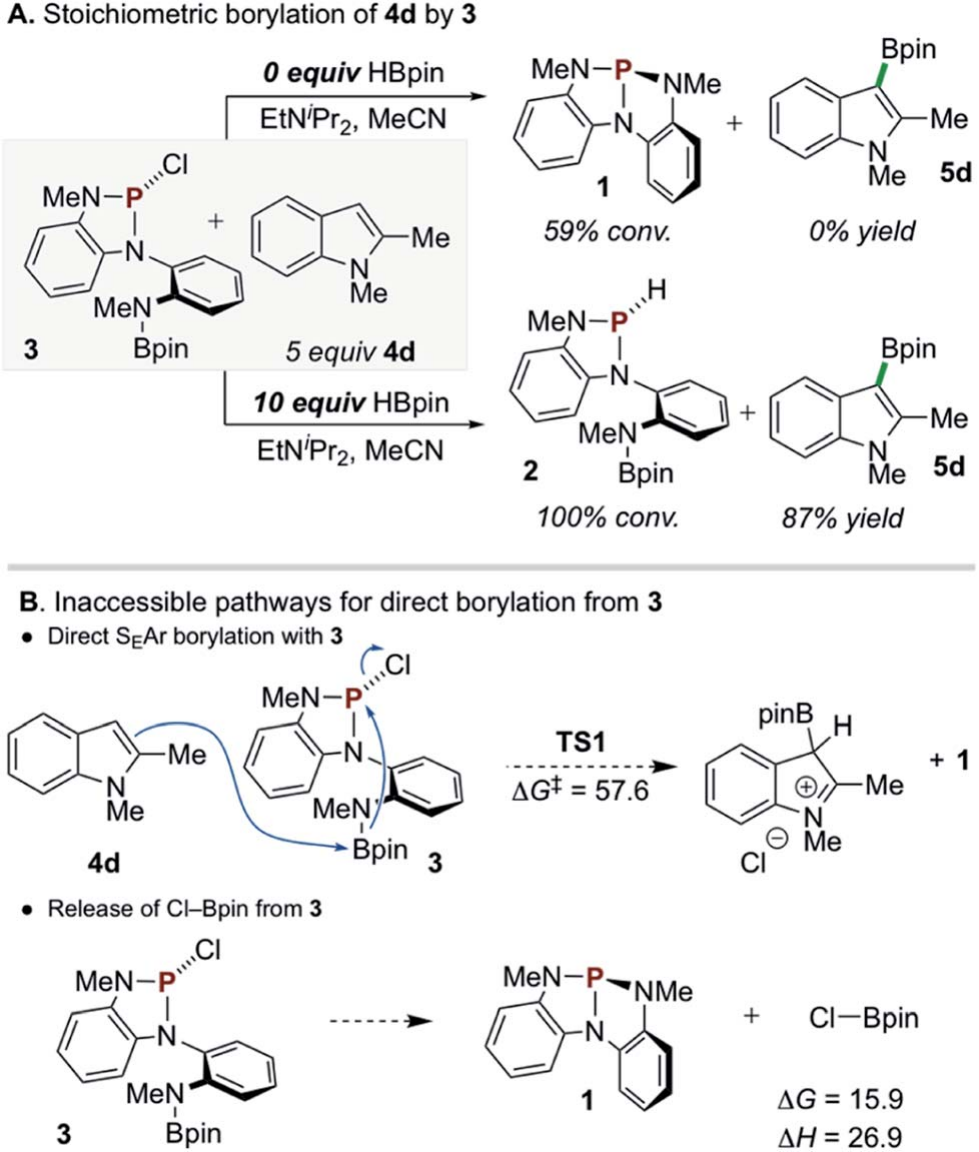
(A) Stoichiometric borylation of 4d occurs from 3 only in the presence of additional HBpin. EtN^i^Pr_2_ (1 equiv.), MeCN, 60 °C, 3 h. ^1^H NMR yields compared to internal standard. (B) DFT calculations indicate (top) direct borylation from 3 is kinetically inaccessible, while (bottom) release of electrophilic Cl–Bpin from 3 is substantially thermodynamically uphill. DFT calculations were performed at the M06-2X/6-311+G(d,p), SMD(MeCN)//M06-2X/6-31G(d), SMD(MeCN) level of theory. All energies are in kcal mol^−1^.

A single turnover experiment reacting 3 with 4d under conditions that lack exogenous chloroalkane reagent but otherwise approximate the catalytic reaction (*i.e.* containing HBpin and EtN^i^Pr_2_) resulted in formation of borylated product 5d in 87% yield with respect to 3 (Scheme 5A, bottom). In this reaction, *P*-chloro diazaphospholene 3 is converted cleanly into *P*-hydrido diazaphospholene 2 with no observable intermediates along the reaction pathway (see ESI† for *in situ*^1^H and ^31^P NMR reaction profile). However, an analogous stoichiometric reaction of 3 with 4d omitting the additional HBpin did not lead to C–H borylation (Scheme 5A, top); moreover, ^31^P NMR spectra demonstrate the conversion of *P*-chloro diazaphospholene 3 to the nontrigonal phosphorus triamide 1 in this experiment. Evidently, *P*-chloro diazaphospholene 3 is, in itself, necessary but insufficient to effect the C–H borylation of 4d under these single turnover conditions. Although a downstream borylating species arising from the interaction of 3 and HBpin might be presumed, the simple mixture of 3 and HBpin at 60 °C does not yield any spectroscopic changes. Regrettably, then, the identity of the active borylating species cannot be unambiguously assigned at this time *via* experimental means.

Density functional theory (DFT) calculations were performed to investigate the reactivity of 3 as the active borylating species or precursor. Consistent with the aforementioned experimental results, direct S_E_Ar borylation of 4d with the pendant Bpin moiety of 3 (TS1, Δ*G*^‡^ = 57.6 kcal mol^−1^) requires an insurmountably high activation energy (Scheme 5B, top).^25^ Further, unimolecular decomposition of 3 to generate 1 and electrophilic Cl–Bpin was found to be substantially uphill (Scheme 5B, bottom, Δ*G* = 15.9 kcal mol^−1^),^26^ indicating that, while 1 is observed as a decay product of 3 in the presence of 4d and EtN^i^Pr_2_ (see Scheme 5A, top), it is likely not generating Cl–Bpin in this process due to the energetic penalty.

**Scheme 6:**
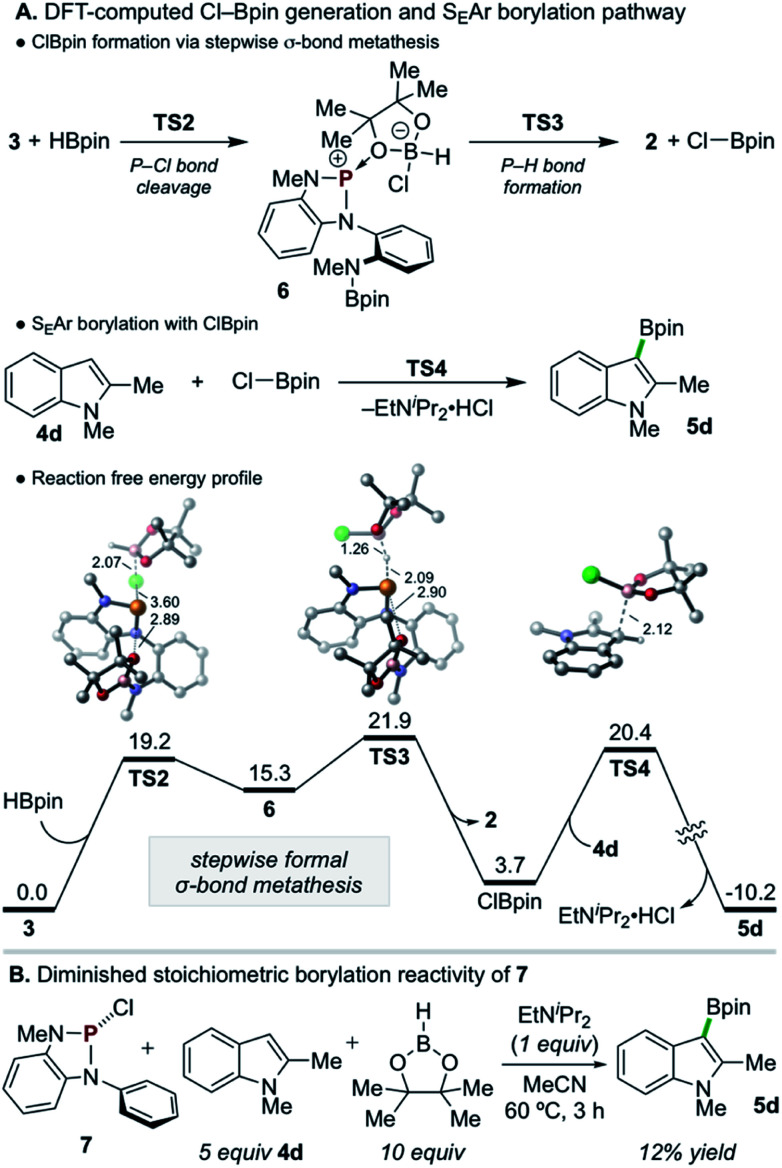
(A) DFT calculations indicate (top) a kinetically accessible, minimally uphill stepwise metathesis reaction between 3 and H–Bpin to generate Cl–Bpin, with a contraction of *d*(P–O) in TS2 and TS3, and (bottom) facile S_E_Ar borylation of 4d with Cl–Bpin. DFT calculations were performed at the M06-2X/6-311+G(d,p), SMD(MeCN)//M06-2X/6-31G(d), SMD(MeCN) level of theory. Bond distances are in angstroms. All energies are in kcal mol^−1^. Hydrogen atoms in 3D structures are omitted for clarity. (B) Stoichiometric borylation of 4d with 7, lacking the *N*(Me)Bpin moiety, is significantly diminished relative to 3.

Given the evident requirement of H–Bpin in addition to 3 to achieve C–H borylation, DFT calculations were performed on higher-order reaction pathways (see ESI† for full details). These calculations suggest that highly electrophilic chloropinacolborane (Cl–Bpin)^27^ can be generated *via* a stepwise, formal σ-bond metathesis between H–Bpin and the P–Cl bond of 3 with kinetically accessible barriers of Δ*G*^‡^ = 19.2 and 21.9 kcal mol^−1^ for P–Cl cleavage (TS2) and P–H formation (TS3), respectively, *via* the intermediacy of 6 (Scheme 6A). Although the conversion of H–Bpin to Cl–Bpin is endergonic by 3.7 kcal mol^−1^, the subsequent borylation of 4d with Cl–Bpin proceeds with a low relative barrier of 16.7 kcal mol^−1^ (TS4) and is highly exergonic upon deprotonation and rearomatization.

Although such an endergonic exchange reaction between HBpin and 3 was not observed experimentally (*via in situ* NMR of the full reaction mixture or isolated reaction of the two species), insights can be drawn from analysis of the stepwise metathesis pathway. In the course of the reaction of 3 and HBpin to form 2, the distance between an O-atom of the Bpin moiety of 3 and the electropositive P-atom shortens in the rate-limiting TS (*d*(P–O) = 2.95 Å and 2.90 Å in 3 and TS3, respectively), indicating the formation of Cl–Bpin is possibly promoted by a stabilizing P–O interaction in the transition state. To probe this hypothesis, diazaphospholene 7, which lacks the pendant N(Me)Bpin moiety, was synthesized and exposed to the single turnover conditions (Scheme 6B). Consistent with the delineated hypothesis, product 5d was formed in substantially diminished yield of 12%.^28^ Thus, this unexpected Lewis base-stabilization effect provides a potential guiding principle for future development.

**Scheme 7:**
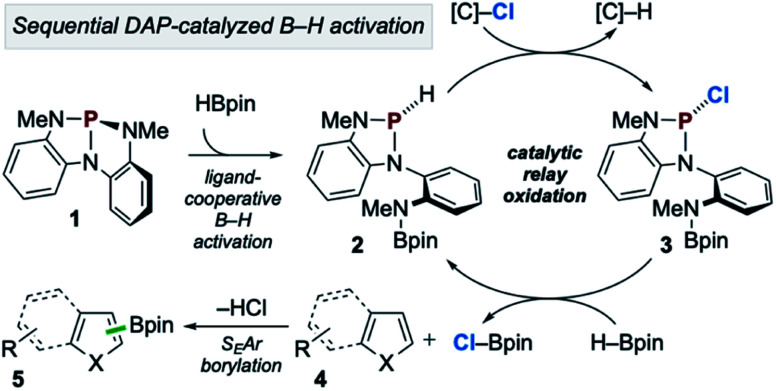
Proposed catalytic reaction pathway for C–H borylation of heteroarenes *via* organophosphorus-catalyzed relay oxidation.

In accordance with the preceding experimental and computational results, the mechanism in Scheme 7 is proposed. First, during the induction period phosphorus triamide 1 activates HBpin to generate *P*-hydrido diazaphospholene 2. Then, catalytic relay oxidation occurs in which 2 reacts with chloroalkane to generate *P*-chloro diazaphospholene 3, followed by stepwise metathesis with H–Bpin to provide Cl–Bpin and regenerate 2. In effect, the facile oxidation of the P–H bond of 2 enables the downstream oxidation of the B–H bond of H–Bpin. Subsequently, the catalytically generated, substoichiometric electrophile Cl–Bpin can undergo S_E_Ar borylation with substrate to provide borylated product. Notably, Cl–Bpin is known to be extremely unstable and difficult to prepare,^27^ and its reactivity in S_E_Ar borylation has therefore not been previously reported. Thus, a catalytic platform for the *in situ* substoichiometric generation of Cl–Bpin from H–Bpin is a novel approach to borylative chemistry *via* relay oxidation, as proposed in Scheme 1.

## Conclusions

To summarize, nontrigonal phosphorus triamide 1 represents the first organophosphorus catalyst to enable C–H borylation of electron-rich heteroarenes. In this transformation, a novel mode of catalysis is realized by targeted diversion of an established hydroboration pathway of 2 with a sacrificial chloroalkane electrophile *via* Atherton–Todd oxidation, diverting the reactivity towards electrophilic borylation from 3. Computational studies support the *in situ* generation of the highly electrophilic Cl–Bpin, which serves as a fleeting intermediate for S_E_Ar borylation, avoiding the difficulties inherent to working with stoichiometrically-generated Cl–Bpin. This novel phosphacatalytic system is poised for further study of the impact of catalyst structure on this mode of catalysis in borylative transformations.

## Conflicts of interest

There are no conflicts to declare.

## Supplementary Material

SC-012-D0SC05620K-s001
