# Supplementary material for: Organophosphorus-catalyzed relay oxidation of H-Bpin: electrophilic C–H borylation of heteroarenes
Source: Chem Sci. 2020 Nov 19;12(3):1031–7. doi: 10.1039/d0sc05620k (PMC8179051; doi:10.1039/d0sc05620k)

Electronic Supplementary Material (ESI) for Chemical Science.
This journal is © The Royal Society of Chemistry 2020

Organophosphorus-Catalyzed Relay Oxidation of H–Bpin: Electrophilic C–H Borylation of Heteroarenes

Jeffrey M. Lipshultz,[†] Yue Fu,[‡] Peng Liu,[‡,*] and Alexander T. Radosevich[†,*]

[†]Department of Chemistry, Massachusetts Institute of Technology
Cambridge, Massachusetts, 02139, United States
email: radosevich@mit.edu

[‡]Department of Chemistry, University of Pittsburgh
Pittsburgh, Pennsylvania, 15260, United States
email: pengliu@pitt.edu

Table of Contents

I. General Materials and Methods S2
II. Optimization of Reaction Conditions S3
III. Examples of Catalytic C–H Borylation S4
A. General Procedure S4
B. Analytical Data S5
IV. Mechanistic Investigations S12
A. Time study of catalytic borylation of **4d** by **1** S12
B. In situ NMR time study of catalytic borylation of **4d** by **1** S14
C. In situ NMR time study of catalytic borylation of **4d** by **3** S16
D. Stoichiometric borylation of **4d** by **3** S18
E. In situ NMR time study of stoichiometric borylation of **4d** with HBpin by **3** S20
F. Stoichiometric borylation of **4d** with HB(pin-d_6) by **3** S22
G. Stoichiometric borylative exchange of **5d** with HB(pin-d_6) by **3** S24
H. Catalytic borylative exchange of **5d** with HB(pin-d_6) by **1** S25
I. Catalytic borylation of **4d** by **7** S27
J. Stoichiometric borylation of **4d** with HBpin by **7** S28
K. Synthesis of HB(pin-d_6) S29
V. Computational Results and Discussion S30
A. General computational details S30
B. Computed reaction energy profiles S31
C. Origin of the reactivity difference between **3** and **7** S33
VI. Synthesis of Phosphorus Compounds S35
A. Synthesis of **1** S35
B. Synthesis of **3** S38
C. Synthesis of **7** S39
VII. References S40
VIII. Cartesian Coordinates and Energies of Optimized Structures S41
IX. Spectral Data S113

I. General Materials and Methods

All reagents were purchased from commercial vendors (Sigma-Aldrich, Alfa Aesar, Acros, TCI, Oakwood Chemical, Combi-Blocks) and used without further purification unless otherwise indicated. Indicated substrates were synthesized according to literature procedure. Pinacolborane (Oakwood Chemical, 98%) was distilled under vacuum, brought into the glovebox, and stored indefinitely in a -35 ºC freezer. *N*,*N*-Diisopropylethylamine (Sigma Aldrich, purified by redistillation, 99.5%) was distilled over KOH under N_2 and brought into the glovebox. α,α-Dichlorotoluene (TCI, 95%) and $CHCl_3$ (Sigma Aldrich, anhydrous, contains amylenes as stabilizer, ≥99%) were degassed by three cycles of freeze-pump-thaw in Schlenk tubes, brought into the glovebox, and stored in amber bottles. Acetonitrile was purified and collected under argon using a Glass Contour Solvent Purification System. All other solvents were ACS grade or better and were used without further purification unless otherwise noted. Manipulations were conducted under an atmosphere of dry N_2 gas unless otherwise noted. Reactions conducted in 4 mL vials used white polypropylene vial caps with PTFE faced foamed polyethylene liner from Wheaton (Item# W238520-1325). Column chromatography was carried out on silica gel (SiliFlash® Irregular Silica Gel, P60 40-63μm). ^{1}H, ^{13}C, and ^{31}P NMR were collected with Bruker Neo 600 (QCI-F helium cryoprobe), Bruker Neo 500 (BBO Prodigy nitrogen cryoprobe or BBFO SmartProbe), or Bruker AVANCE III HD 400 (BBO Prodigy nitrogen cryoprobe) spectrometers and processed using MestReNova software. ^{1}H NMR chemical shifts are given in ppm with respect to solvent residual peak ($CDCl_3$, δ 7.26 ppm). ^{2}H NMR chemical shifts are given in ppm with respect to added solvent peak ($CDCl_3$, δ 7.26 ppm). $^{13}C\{^{1}H\}$ NMR shifts are given in ppm with respect to solvent peak ($CDCl_3$ δ 77.16 ppm). ^{31}P NMR shifts are given in ppm with respect to 85% H_3PO_4 (δ 0.0 ppm) as an external standard. Multiplicities are described as s = singlet, bs = broad singlet, d = doublet, t = triplet, q = quartet, dd = doublet of doublets, td = triplet of doublets, m = multiplet. Coupling constants are reported in Hertz (Hz). High-resolution mass spectra were obtained at the Mass Spectrometry Laboratory in the Department of Chemistry Instrumentation Facility, MIT, using either Agilent QTOF 6545 with ESI ionization source or a JEOL AccuTOF-DART (JMS-T100LP, ionSense DART source).

II. Optimization of Reaction Conditions

In a glovebox, a stock solution was prepared in a 2 mL volumetric flask. The 2 mL volumetric flask was charged with **1** and 1-methyl-1*H*-pyrrole (**4a**, 40.6 mg, 0.5 mmol, 1 equiv). CH_3CN was added to reach 2 mL volume. 500 µL of this stock solution was added to a 4 mL vial previously charged with a magnetic stir bar. To each reaction vessel was then added by blunt-tipped microsyringe base and oxidant, followed by pinacolborane. The reaction was capped, removed from the glovebox, and heated in a pie block to the indicated temperature for 16 h with 300 rpm stirring. After 16 h, each reaction was allowed to cool to ambient temperature and 1,1,2,2-tetrachloroethane (10 µL, 0.093 mmol, 0.075 equiv, 98%) was added as internal standard. An aliquot was then diluted in $CDCl_3$ and analyzed by ^{1}H NMR. The yield was determined by relative integration between $C_2H_2Cl_4$ (5.95 ppm, s, 2 H, 0.075 equiv, integrate to 149.09), starting material **4a** (6.39 ppm, bs, 1H), and product **5a** (7.60 ppm, t, 1H). Number of scans = 8 and relaxation delay = 4 seconds.

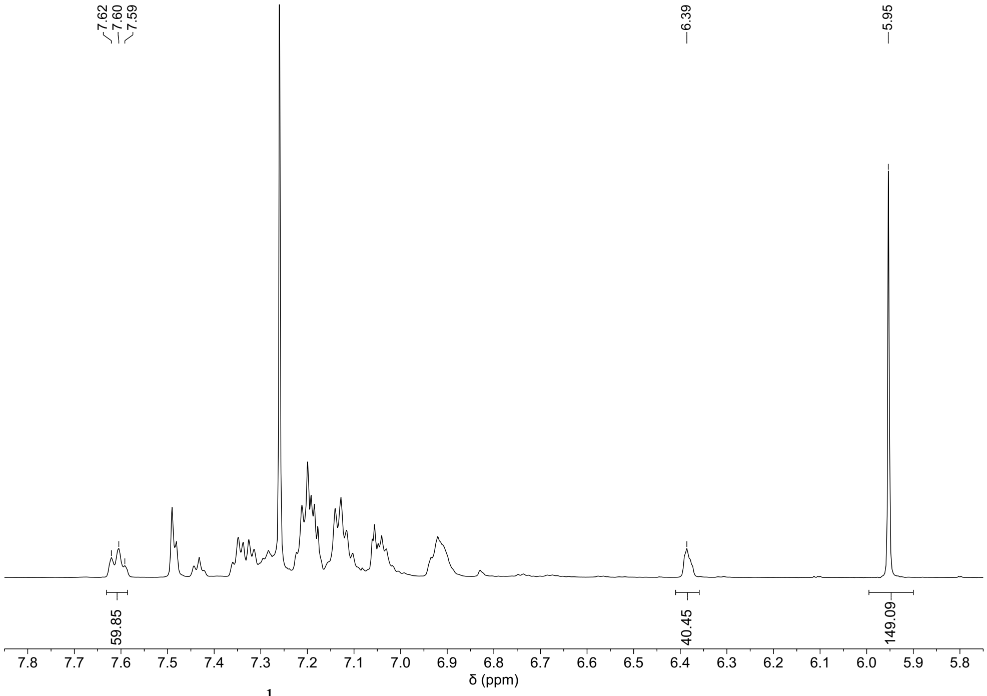

Figure S1. Representative ^{1}H NMR spectrum for yield determination for optimization of borylation of **4a** to **5a** by **1** (optimal conditions).

III. Examples of Catalytic C–H Borylation

A. General Procedure

In the glovebox, a 2.5x scale stock solution was prepared in a 5 mL volumetric flask. The 5 mL volumetric flask was charged with catalyst **1** (63.6 mg, 0.25 mmol, 0.20 equiv), *N*,*N*-diisiopropylethylamine (323.1 mg, 2.5 mmol, 2 equiv), and α,α-dichlorotoluene (211.9 mg, 1.25 mmol, 1 equiv, 95%). CH_3CN was added to reach 5 mL volume. 2 mL of this stock solution was added to a 4 mL vial previously charged with heteroarene substrate (0.5 mmol) and magnetic stir bar (note that reactions were set up in pairs such that each 5 mL stock solution was used for two 0.5 mmol scale reactions). To the reaction vessel was then added pinacolborane (145 µL, 1 mmol, 2 equiv). The reaction was capped, removed from the glovebox, and heated in a pie block to the indicated reaction temperature for 16 hours with 300 rpm stirring, unless otherwise indicated. After 16 hours, the reaction was allowed to cool to ambient temperature and transferred to a 40 mL vial with washing with EtOAc. The crude reaction was concentrated, dissolved in acetone, and adsorbed onto silica gel under vacuum. The crude residue was then purified by flash column chromatography on silica gel (dry loading) by eluting with the indicated solvent.

B. Analytical Data

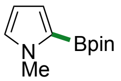

1-Methyl-2-(4,4,5,5-tetramethyl-1,3,2-dioxaborolan-2-yl)-1*H*-pyrrole (5a).

Prepared according to the general procedure using 1-methyl-1*H*-pyrrole (40.6 mg, 0.5 mmol). Reaction conducted at 100 ºC. Column eluted with 8% EtOAc/hexanes. Yield: 60% (62.3 mg). Off-white solid.

^{1}H NMR (400 MHz, $CDCl_3$) δ 6.82 – 6.80 (m, 2H), 6.16 (dd, J = 3.6, 2.4 Hz, 1H), 3.84 (s, 3H), 1.31 (s, 12H).

^{13}C NMR (101 MHz, $CDCl_3$) δ 128.33, 122.03, 108.52, 83.21, 36.74, 24.94.

^{11}B NMR (128 MHz, $CDCl_3$) δ 28.12.

HRMS (DART) calculated for $C_{11}H_{19}BO_2N$ $[M+H]^+$ 208.1509, found 208.1519.

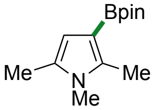

1,2,5-Trimethyl-3-(4,4,5,5-tetramethyl-1,3,2-dioxaborolan-2-yl)-1*H*-pyrrole (5b).

Prepared according to the general procedure using 1,2,5-trimethyl-1*H*-pyrrole (55.1 mg, 0.5 mmol, 99%). Reaction conducted at 80 ºC. Column eluted with 5% EtOAc/hexanes. Yield: 67% (78.2 mg). Off-white solid.

^{1}H NMR (600 MHz, $CDCl_3$) δ 6.13 (s, 1H), 3.38 (s, 3H), 2.41 (s, 3H), 2.20 (s, 3H), 1.30 (s, 12H).

^{13}C NMR (151 MHz, $CDCl_3$) δ 138.61, 128.72, 110.93, 82.33, 30.23, 24.94, 12.55, 12.47.

^{11}B NMR (128 MHz, $CDCl_3$) δ 30.19.

HRMS (ESI) calculated for $C_{13}H_{23}BO_2N$ $[M+H]^+$ 236.1819 found 236.1822.

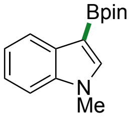

1-Methyl-3-(4,4,5,5-tetramethyl-1,3,2-dioxaborolan-2-yl)-1*H*-indole (5c).

Prepared according to the general procedure using 1-methyl-1*H*-indole (65.6 mg, 0.5 mmol). Reaction conducted at 80 ºC. Column eluted with 10% EtOAc/hexanes. Yield: 73% (94.3 mg). Off-white solid.

^{1}H NMR (600 MHz, $CDCl_3$) δ 8.03 (d, J = 7.8 Hz, 1H), 7.52 (s, 1H), 7.32 (d, J = 8.2 Hz, 1H), 7.23 (t, J = 7.6 Hz, 1H), 7.18 (t, J = 8.0 Hz, 1H), 3.80 (s, 3H), 1.36 (s, 12H).

^{13}C NMR (151 MHz, $CDCl_3$) δ 138.57, 137.96, 132.64, 122.79, 121.89, 120.35, 109.30, 82.86, 33.12, 25.07.

^{11}B NMR (128 MHz, $CDCl_3$) δ 30.30.

HRMS (ESI) calculated for $C_{15}H_{21}BO_2N$ $[M+H]^+$ 258.1663 found 258.1666.

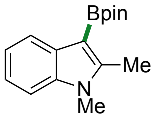

1,2-Dimethyl-3-(4,4,5,5-tetramethyl-1,3,2-dioxaborolan-2-yl)-1*H*-indole (5d).

Prepared according to the general procedure using 1,2-dimethyl-1*H*-indole (74.1 mg, 0.5 mmol, 98%). Reaction conducted at 60 ºC. Column eluted with 5% Et_2O/hexanes. Yield: 86% (117.0 mg). White solid.

^{1}H NMR (600 MHz, $CDCl_3$) δ 8.00 (d, J = 6.9 Hz, 1H), 7.24 (d, J = 7.9 Hz, 1H), 7.16 (td, J = 7.5, 1.5 Hz, 1H), 7.12 (td, J = 7.4, 1.3 Hz, 1H), 3.67 (s, 3H), 2.64 (s, 3H), 1.36 (s, 12H).

^{13}C NMR (151 MHz, $CDCl_3$) δ 147.64, 138.09, 132.62, 121.95, 120.94, 120.23, 108.61, 82.42, 29.61, 25.15, 12.77.

^{11}B NMR (128 MHz, $CDCl_3$) δ 30.71.

HRMS (ESI) calculated for $C_{16}H_{23}BO_2N$ $[M+H]^+$ 272.1819, found 272.1819.

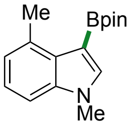

1,4-Dimethyl-3-(4,4,5,5-tetramethyl-1,3,2-dioxaborolan-2-yl)-1*H*-indole (5e).

Prepared according to the general procedure using 1,4-dimethyl-1*H*-indole[1] (72.6 mg, 0.5 mmol). Reaction conducted at 80 ºC. Column eluted with 8% EtOAc/hexanes. Yield: 74% (100.4 mg). Off-white solid.

^{1}H NMR (600 MHz, $CDCl_3$) δ 7.56 (s, 1H), 7.19 – 7.08 (m, 2H), 6.95 (d, J = 6.4 Hz, 1H), 3.77 (s, 3H), 2.77 (s, 3H), 1.35 (s, 12H).

^{13}C NMR (151 MHz, $CDCl_3$) δ 139.83, 138.55, 133.22, 130.84, 122.05, 121.77, 106.98, 82.98, 33.18, 24.93, 21.33.

^{11}B NMR (128 MHz, $CDCl_3$) δ 30.47.

HRMS (ESI) calculated for $C_{16}H_{23}BO_2N$ $[M+H]^+$ 272.1819, found 272.1824.

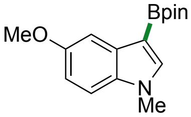

5-Methoxy-1-methyl-3-(4,4,5,5-tetramethyl-1,3,2-dioxaborolan-2-yl)-1*H*-indole (5f).

Prepared according to the general procedure using 5-methoxy-1-methyl-1*H*-indole[1] (82.3 mg, 0.5 mmol, 98%). Reaction conducted at 80 ºC. Column eluted with 20% EtOAc/hexanes. Yield: 87% (124.4 mg). Off-white solid.

^{1}H NMR (600 MHz, $CDCl_3$) δ 7.51 (t, J = 2.6 Hz, 1H), 7.48 (s, 1H), 7.20 (dd, J = 8.8, 2.5 Hz, 1H), 6.89 (dd, J = 8.8, 2.7 Hz, 1H), 3.90 (s, 3zH), 3.76 (s, 3H), 1.36 (s, 12H).

^{13}C NMR (151 MHz, $CDCl_3$) δ 154.87, 138.97, 133.37, 133.31, 111.84, 109.93, 104.95, 82.82, 56.14, 33.29, 25.07.

^{11}B NMR (128 MHz, $CDCl_3$) δ 30.14.

HRMS (ESI) calculated for $C_{16}H_{23}BO_3N$ $[M+H]^+$ 288.1768, found 288.1765.

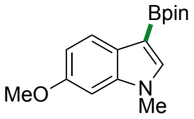

6-Methoxy-1-methyl-3-(4,4,5,5-tetramethyl-1,3,2-dioxaborolan-2-yl)-1*H*-indole (5g).

Prepared according to the general procedure using 6-methoxy-1-methyl-1*H*-indole[2] (80.6 mg, 0.5 mmol). Reaction conducted at 80 ºC. Column eluted with 10% EtOAc/hexanes. Yield: 83% (119.6 mg). Pale tan solid.

^{1}H NMR (600 MHz, $CDCl_3$) δ 7.88 (d, J = 8.6 Hz, 1H), 7.42 (s, 1H), 6.84 (dd, J = 8.6, 2.3 Hz, 1H), 6.78 (d, J = 2.3 Hz, 1H), 3.88 (s, 3H), 3.74 (s, 3H), 1.35 (s, 12H).

^{13}C NMR (151 MHz, $CDCl_3$) δ 156.53, 138.63, 137.66, 126.75, 123.36, 110.01, 93.09, 82.85, 55.90, 33.11, 25.06.

^{11}B NMR (128 MHz, $CDCl_3$) δ 29.97.

HRMS (ESI) calculated for $C_{16}H_{23}BO_3N$ $[M+H]^+$ 288.1768, found 288.1773.

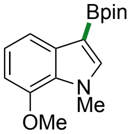

7-Methoxy-1-methyl-3-(4,4,5,5-tetramethyl-1,3,2-dioxaborolan-2-yl)-1*H*-indole (5h).

Prepared according to the general procedure using 7-methoxy-1-methyl-1*H*-indole[1] (80.6 mg, 0.5 mmol). Reaction conducted at 100 ºC. Column eluted with 10% EtOAc/hexanes. Yield: 85% (122.7 mg). Off-white solid.

^{1}H NMR (600 MHz, $CDCl_3$) δ 7.62 (d, J = 8.0 Hz, 1H), 7.38 (s, 1H), 7.04 (t, J = 7.8 Hz, 1H), 6.62 (d, J = 7.7 Hz, 1H), 4.05 (s, 3H), 3.92 (s, 3H), 1.36 (s, 12H).

^{13}C NMR (151 MHz, $CDCl_3$) δ 147.90, 139.46, 135.10, 127.57, 120.80, 115.58, 115.56, 102.78, 102.76, 82.78, 82.77, 55.52, 36.86, 25.04.

^{11}B NMR (128 MHz, $CDCl_3$) δ 30.25.

HRMS (ESI) calculated for $C_{16}H_{23}BO_3N$ $[M+H]^+$ 288.1768, found 288.1769.

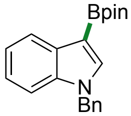

1-Benzyl-3-(4,4,5,5-tetramethyl-1,3,2-dioxaborolan-2-yl)-1*H*-indole (5i).

Prepared according to the general procedure using 1-benzyl-1*H*-indole (106.8 mg, 0.5 mmol, 97%). Reaction conducted at 100 ºC. Column eluted with 8% EtOAc/hexanes. Yield: 45% (74.2 mg). Off-white solid. Recovered starting material: 55% (57.3 mg). 100% yield based on recovered starting material.

^{1}H NMR (600 MHz, $CDCl_3$) δ 8.07 (dd, J = 6.0, 3.1 Hz, 1H), 7.61 (s, 1H), 7.33 – 7.24 (m, 3H), 7.18 (dd, J = 6.0, 3.1 Hz, 2H), 7.15 (d, J = 6.8 Hz, 2H), 5.32 (s, 2H), 1.38 (s, 12H).

^{13}C NMR (151 MHz, $CDCl_3$) δ 137.95, 137.50, 137.08, 132.86, 128.91, 128.89, 127.85, 127.84, 127.17, 127.15, 122.88, 122.06, 120.53, 109.86, 82.92, 50.51, 25.08.

^{11}B NMR (128 MHz, $CDCl_3$) δ 30.22.

HRMS (ESI) calculated for $C_{21}H_{25}BO_2N$ $[M+H]^+$ 334.1977, found 334.1972.

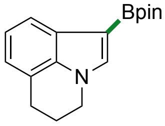

1-(4,4,5,5-Tetramethyl-1,3,2-dioxaborolan-2-yl)-5,6-dihydro-4H-pyrrolo[3,2,1-*ij*]quinoline (5j).

Prepared according to the general procedure using lilolidine (80.2 mg, 0.5 mmol, 98%). Reaction conducted at 80 ºC. Column eluted with 10% EtOAc/hexanes. Yield: 98% (138.8 mg). Off-white solid.

^{1}H NMR (600 MHz, $CDCl_3$) δ 7.79 (d, J = 7.9 Hz, 1H), 7.53 (s, 1H), 7.08 (d, J = 15.0 Hz, 1H), 6.92 (d, J = 7.5 Hz, 1H), 4.20 – 4.09 (m, 2H), 2.99 (t, J = 6.1 Hz, 2H), 2.22 (dt, J = 11.4, 5.9 Hz, 2H), 1.35 (s, 12H).

^{13}C NMR (151 MHz, $CDCl_3$) δ 135.51, 135.38, 130.33, 121.88, 120.76, 120.15, 118.92, 82.80, 44.59, 25.06, 24.89, 23.13.

^{11}B NMR (128 MHz, $CDCl_3$) δ 30.23.

HRMS (ESI) calculated for $C_{17}H_{23}BO_2N$ $[M+H]^+$ 284.1819, found 284.1815.

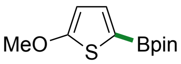

2-(5-Methoxythiophen-2-yl)-4,4,5,5-tetramethyl-1,3,2-dioxaborolane (5k).

In the glovebox, a 2x scale stock solution was prepared in a 2 mL volumetric flask. The 2 mL volumetric flask was charged with catalyst **1** (12.8 mg, 0.05 mmol, 0.05 equiv), 2-methoxythiophene (233.0 mg, 2.0 mmol, 2 equiv, 98%), *N*,*N*-diisiopropylethylamine (129.3 mg, 1.0 mmol, 1 equiv), and α,α-dichlorotoluene (169.5 mg, 1.0 mmol, 1 equiv, 95%). CH_3CN was added to reach 2 mL volume. 1 mL of this stock solution was added to a 4 mL vial previously charged with a magnetic stir bar. To the reaction vessel was then added pinacolborane (72.6 µL, 0.5 mmol, 1 equiv). The reaction was capped, removed from the glovebox, and heated in a pie block to 100 ºC for 16 hours with 300 rpm stirring. After 16 hours, the reaction was allowed to cool to ambient temperature and transferred to a 40 mL vial with washing with EtOAc. The crude reaction was concentrated, dissolved in acetone, and adsorbed onto silica gel under vacuum. The crude residue was then purified by flash column chromatography on silica gel (dry loading) by eluting with 5% EtOAc/hexanes to provide the product as a pale yellow oil. Yield: 37% (44.6 mg). Yield with respect to limiting HBpin.

^{1}H NMR (600 MHz, $CDCl_3$) δ 7.33 (d, J = 3.8 Hz, 1H), 6.30 (d, J = 3.8 Hz, 1H), 3.92 (s, 3H), 1.32 (s, 12H).

^{13}C NMR (151 MHz, $CDCl_3$) δ 172.97, 136.63, 106.28, 83.95, 60.54, 24.87.

^{11}B NMR (128 MHz, $CDCl_3$) δ 28.59.

HRMS (DART) calculated for $C_{11}H_{17}BO_3S$ $[M+H]^+$ 241.1070, found 241.1071.

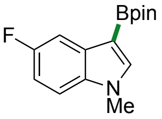

5-Fluoro-1-methyl-3-(4,4,5,5-tetramethyl-1,3,2-dioxaborolan-2-yl)-1*H*-indole (5m).

Prepared according to the general procedure using 5-fluoro-1-methyl-1*H*-indole[1] (74.6 mg, 0.5 mmol). Reaction conducted at 100 ºC for 24 hours. Column eluted with 8% EtOAc/hexanes. Yield: 60% (82.1 mg). Off-white solid.

^{1}H NMR (600 MHz, $CDCl_3$) δ 7.69 (dd, J = 9.8, 2.6 Hz, 1H), 7.53 (s, 1H), 7.21 (dd, J = 8.8, 4.3 Hz, 1H), 6.97 (td, J = 9.0, 2.6 Hz, 1H), 3.78 (s, 3H), 1.36 (s, 12H).

^{13}C NMR (151 MHz, $CDCl_3$) δ 159.46, 157.90, 139.77, 134.57, 133.34, 133.27, 110.26, 110.09, 109.88, 109.81, 107.80, 107.65, 82.99, 82.97, 33.39, 25.05, 25.03.

^{11}B NMR (128 MHz, $CDCl_3$) δ 30.03.

^{19}F NMR (565 MHz, $CDCl_3$) δ -124.58 (td, J = 10.0, 4.6 Hz).

HRMS (DART) calculated for $C_{16}H_{20}FBO_2N$ $[M+H]^+$ 276.1571, found 276.1567.

IV. Mechanistic Investigations

A. Time study of catalytic borylation of **4d** by **1**

In a glovebox, a stock solution was prepared in a 5 mL volumetric flask. The 5 mL volumetric flask was charged with **1** (63.8 mg, 0.25 mmol, 0.20 equiv), 1,2-dimethylindole (**4d**, 185.2 mg, 1.25 mmol, 1 equiv, 98%), *N*,*N*-diisiopropylethylamine (323.1 mg, 2.5 mmol, 2 equiv), and $CHCl_3$ (298.4 mg, 2.5 mmol, 2 equiv). CH_3CN was added to reach 5 mL volume. 300 µL of this stock solution was added to 16x4 mL vials previously charged with a magnetic stir bar each. To each reaction vessel was then added pinacolborane (21.8 µL, 0.15 mmol, 2 equiv). The vials were capped, removed from the glovebox, and heated in a pie block to 60 ºC for 1-16 h with 300 rpm stirring. At the indicated time, each reaction was allowed to cool to ambient temperature and 1,1,2,2-tetrachloroethane (12 µL, 0.112 mmol, 1.49 equiv, 98%) was added as internal standard. An aliquot was then diluted in $CDCl_3$ and analyzed by ^1H NMR. The yield was determined by relative integration between $C_2H_2Cl_4$ (5.95 ppm, s, 2 H, 1.49 equiv, integrate to 298.18), starting material **4d** (6.22 ppm, s, 1H), and product **5d** (7.96 ppm, d, 1H). Number of scans = 8 and relaxation delay = 4 seconds.

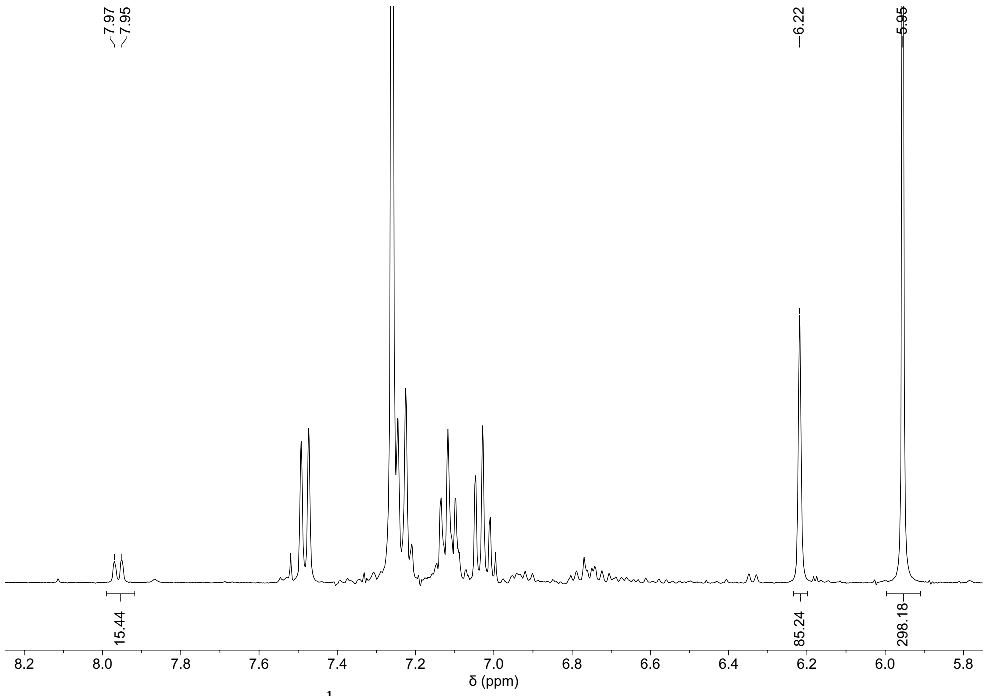

Figure S2. Representative ^1H NMR spectrum for yield determination at t = 3 h.

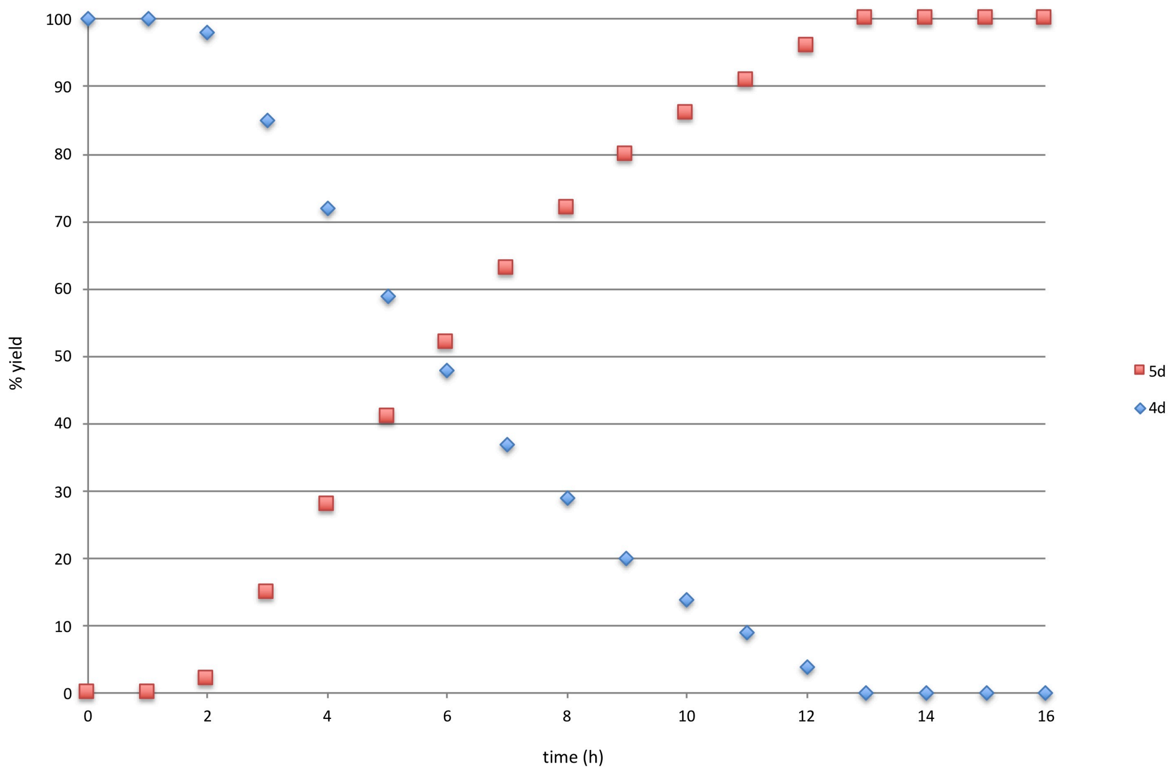

Figure S3. Reaction progress of borylation of **4d** by **1**.

B. In situ NMR time study of catalytic borylation of **4d** by **1**.

In the glovebox, to a 2 mL volumetric flask was added **1** (25.6 mg, 0.1 mmol, 0.2 equiv), 1,2-dimethylindole (**4d**, 74.1 mg, 0.5 mmol, 1 equiv, 98%), *N*,*N*-diisiopropylethylamine (129.3 mg, 1.0 mmol, 2 equiv), and $CHCl_3$ (119.4 mg, 1.0 mmol, 2 equiv). The volume was increased to 2 mL with CD_3CN. To a 4 mL vial was added 0.5 mL of the stock solution (0.125 mmol **4d**), and HBpin (36.3 µL, 0.25 mmol, 2 equiv) was added. The solution was shaken, and transferred to a quartz J-Young NMR tube, which was sealed and removed from the glovebox. The sample was inserted into a 60 °C thermostatted NMR probe and an initial timepoint ($^{31}P\{H\}$, ^{11}B, ^{1}H) was immediately taken. Timepoints were then taken at the following times: 15 minutes, 30 minutes, 1 h, 2 h, 3 h, 4 h, 5 h, 6 h. Yield was assessed by ^{1}H NMR as conversion (no internal standard) by relative integration (total 100) between starting material **4d** (6.22 ppm, s, 1H) and product **5d** (7.89 ppm, d, 1H). $^{31}P\{H\}$ NMR shifts: **1** (159.70 ppm), **2** (85.0 ppm), **3** (146.94 ppm). ^{11}B NMR shifts: HBpin (28.04 ppm, d), **5d** (30.31 ppm), **2** (24.17), **3** (23.35 ppm and 20.97 ppm).

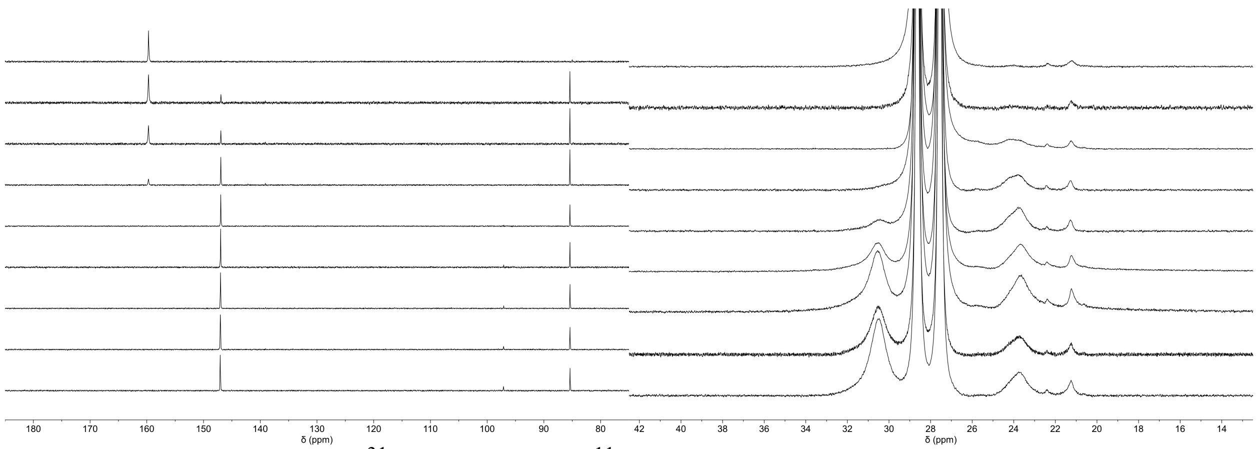

Figure S4. Time-stacked $^{31}P\{H\}$ (left) and ^{11}B (right) NMR spectra of borylation of **4d** by **1**.

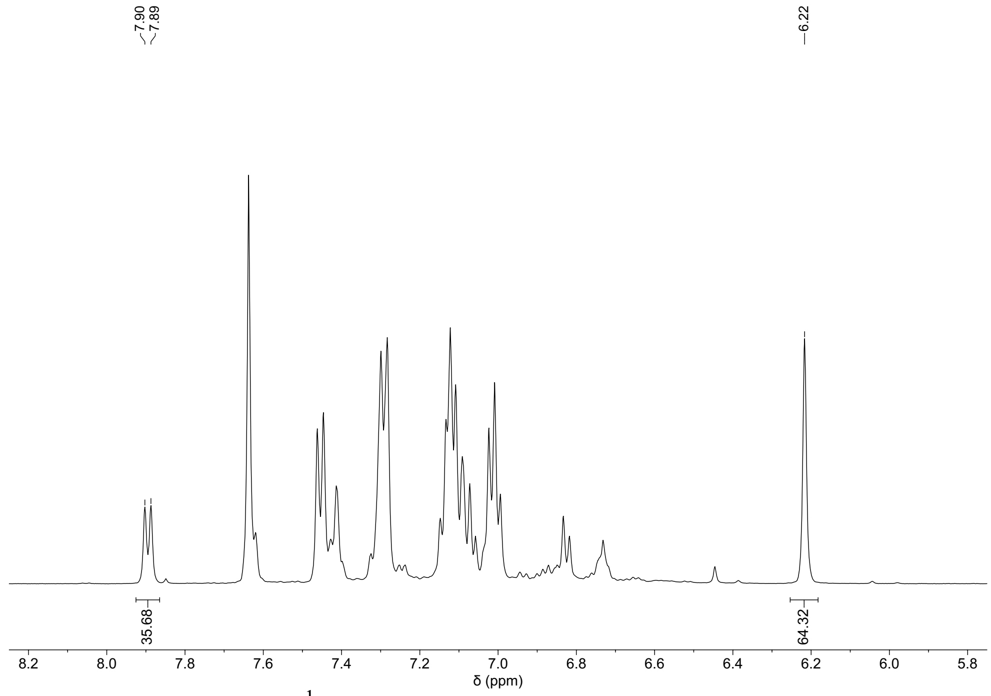

Figure S5. Representative ^{1}H NMR spectrum for yield determination at t = 4 h.

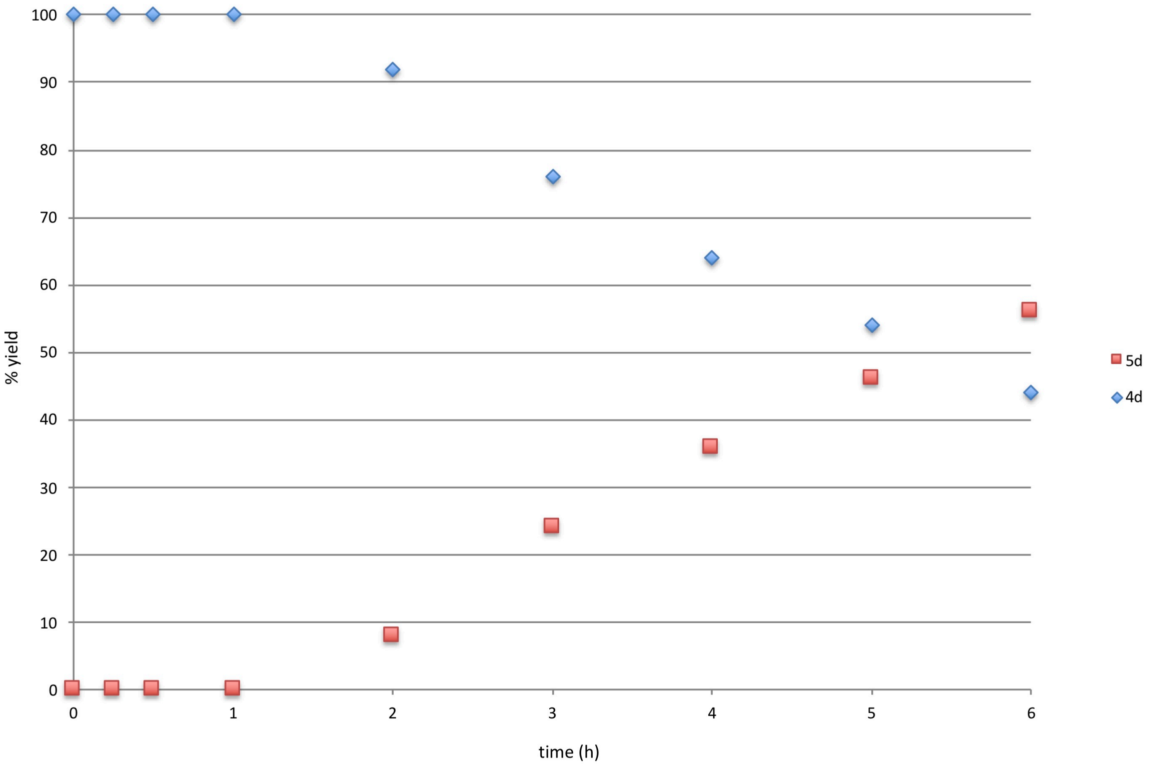

Figure S6. In situ NMR reaction progress of borylation of **4d** by **1**.

C. In situ NMR time study of catalytic borylation of **4d** by **3**.

In the glovebox, to a 4 mL vial was added **3** (10.4 mg, 25 µmol, 0.2 equiv), 1,2-dimethylindole (**4d**, 18.5 mg, 125 µmol, 1 equiv, 98%), and $CHCl_3$ (19.9 µL, 250 µmol, 2 equiv). CD_3CN was added (0.5 mL, 0.25 M), followed by HBpin (36.3 µL, 250 µmol, 2 equiv), and the vial was capped and removed from the glovebox. Under N_2, *N*,*N*-diisiopropylethylamine (43.6 µL, 250 µmol, 2 equiv) was added to the reaction solution, the solution was shaken, and then quickly transferred to a N_2-filled screw cap NMR tube. The sample was inserted into a 60 °C thermostatted NMR probe and an initial timepoint ($^{31}P\{H\}$) was immediately taken. Timepoints ($^{31}P\{H\}$, ^{1}H) were then taken at the following times: 15 minutes, 30 minutes, 1 h, 2 h, 3 h, 4 h, 5 h. Yield was assessed by ^{1}H NMR as conversion (no internal standard) by relative integration (total 100) between starting material **4d** (6.22 ppm, s, 1H) and product **5d** (7.89 ppm, d, 1H). $^{31}P\{H\}$ NMR shifts: **2** (85.0 ppm), **3** (146.94 ppm).

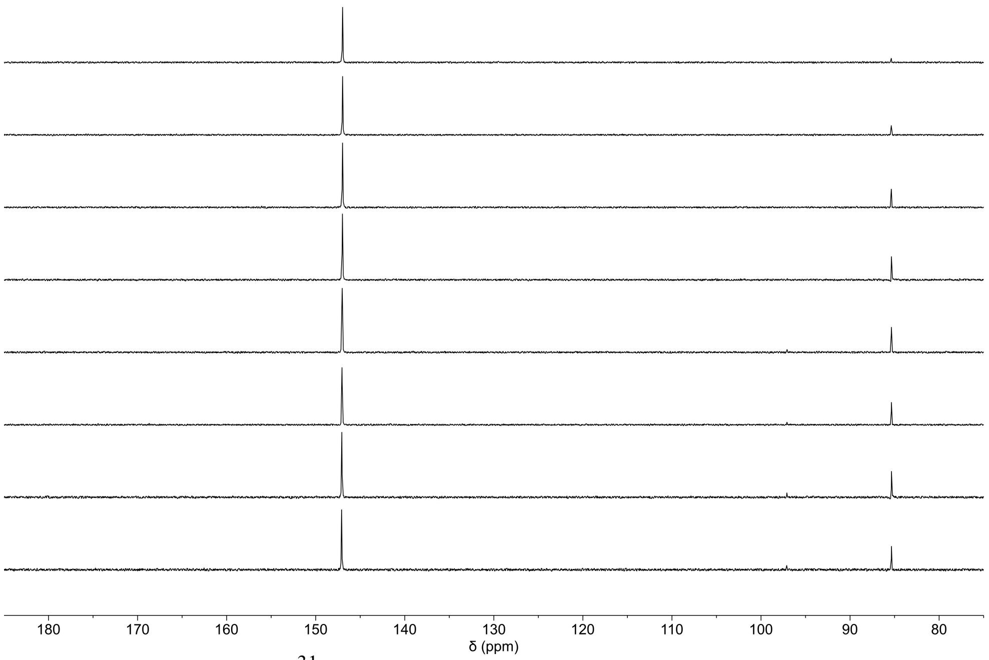

Figure S7. Time-stacked $^{31}P\{H\}$ NMR spectra of catalytic borylation of **4d** by **3**.

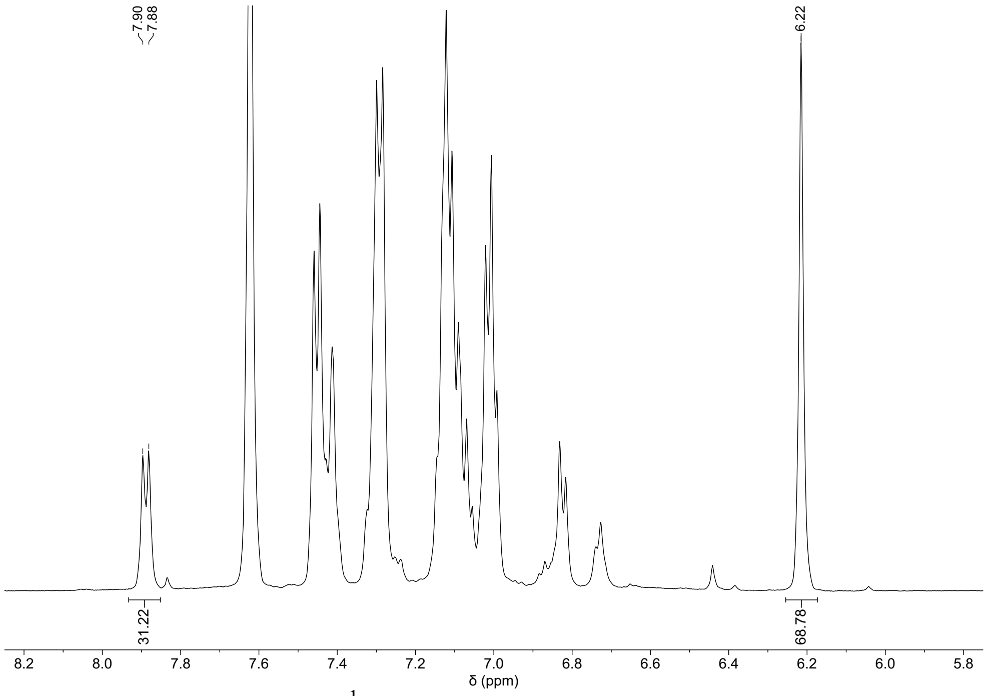

Figure S8. Representative ^{1}H NMR spectrum for yield determination at t = 2 h.

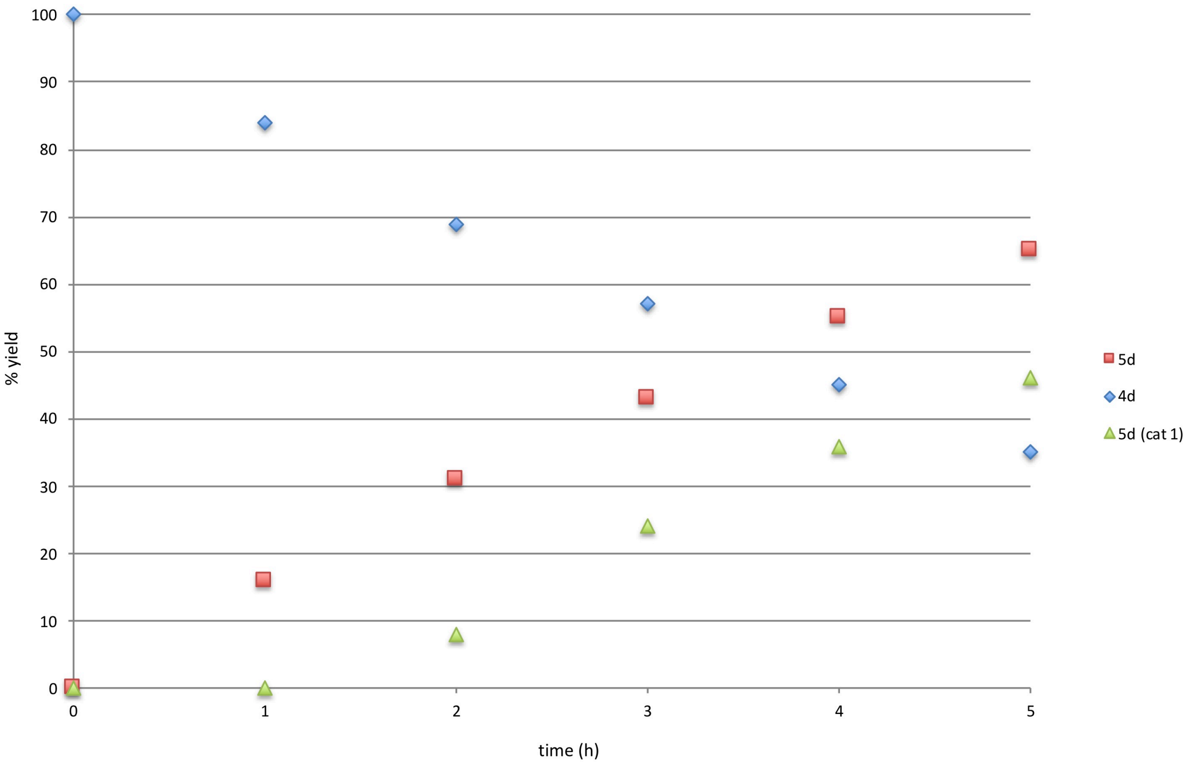

Figure S9. In situ NMR reaction progress of borylation of **4d** by **3** compared to **1**.

D. Stoichiometric borylation of **4d** by **3**.

In a glovebox, two 4 mLs vial equipped with magnetic stir bar were each charged with **3** (10.4 mg, 25 µmol, 1 equiv) and 1,2-dimethylindole (**4d**, 19.5 mg, 125 µmol, 5 equiv, 98%). The solids were dissolved in CH_3CN (0.5 mL, 0.05 M). To one vial (reaction **A**), HBpin (36.3 µL, 250 µmol, 10 equiv) was added, while no HBpin was added to the other (reaction **B**). To each reaction was then added *N*,*N*-diisiopropylethylamine (4.3 µL, 25 µmol, 1 equiv), the reactions were quickly capped and heated to 60 ºC for 3 h. After 3 h, the reactions were cooled to ambient temperature and 50 µL of a stock solution of 1,3,5-trimethoxybenzene (0.25 M in CH_3CN, prepared in a 2 mL volumetric flask with 85 mg 1,3,5-trimethoxybenzene, 500 µmol, 20 equiv, 99%) was added as internal standard to each. An aliquot from each reaction was then diluted in $CDCl_3$ and analyzed by ^{1}H NMR showing 87% yield **5d** (reaction **A**) and 0% yield **5d** (reaction **B**), and a second aliquot from each reaction was diluted in CD_3CN and analyzed by ^{31}P{H} NMR, which showed exclusively **2** (reaction **A**, not shown) and a 59:41 mixture of **1**:**3** (reaction **B**, below). The yield was determined by relative integration between 1,3,5-trimethoxybenzene (6.07 ppm, s, 3 H, 0.5 equiv, integrate to 150), starting material **4d** (6.22 ppm, s, 1H), and product **5d** (7.96 ppm, d, 1H). Number of scans = 8 and relaxation delay = 4 seconds.

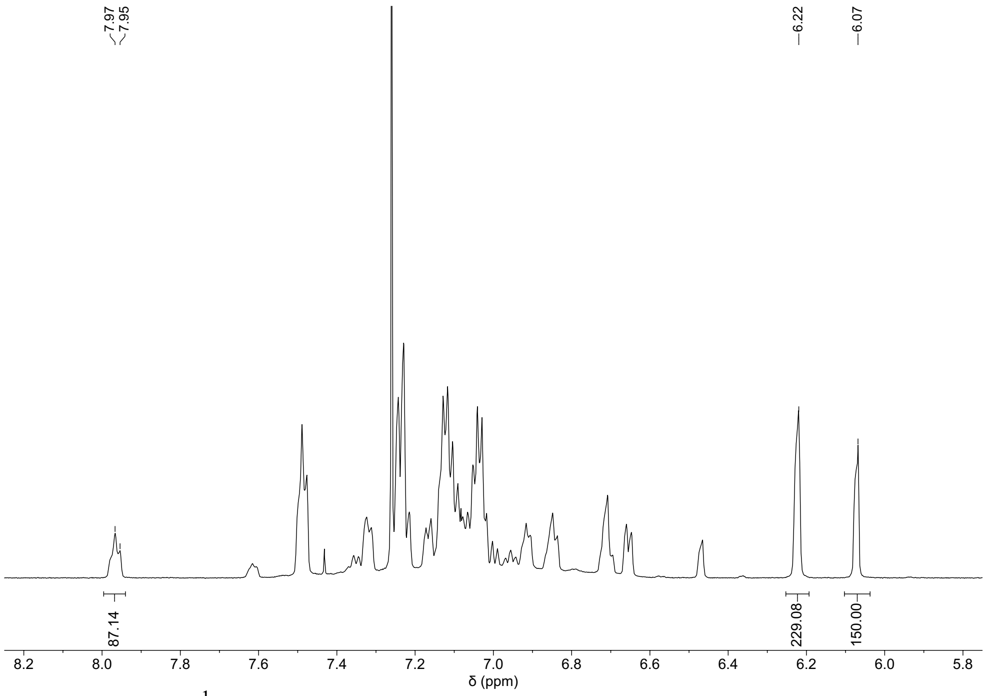

Figure S10. ^{1}H NMR spectrum for yield determination of reaction **A**.

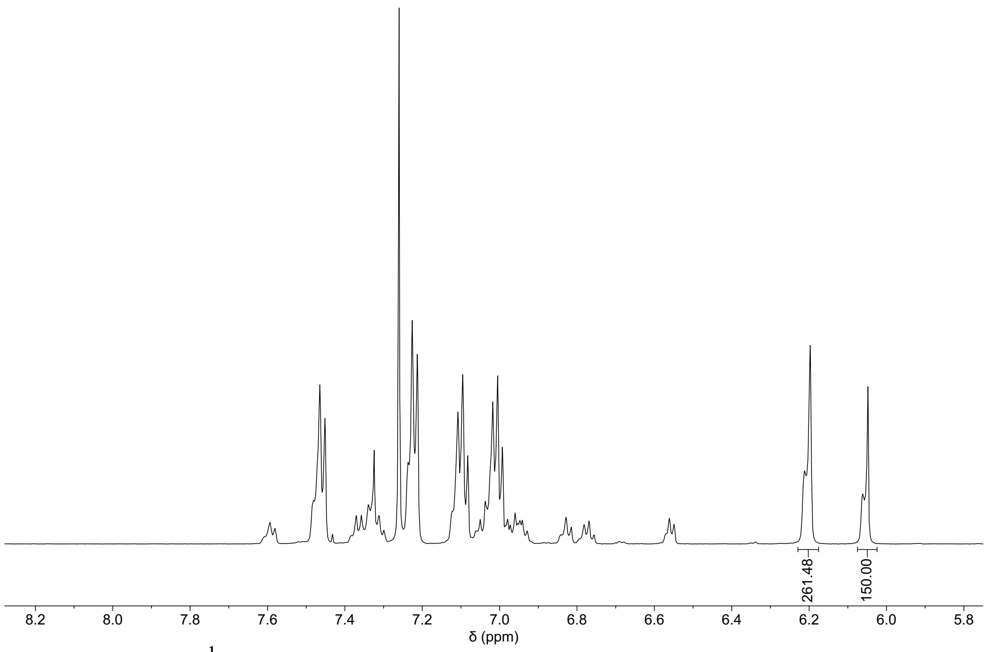

Figure S11. ^{1}H NMR spectrum for yield determination of reaction **B**.

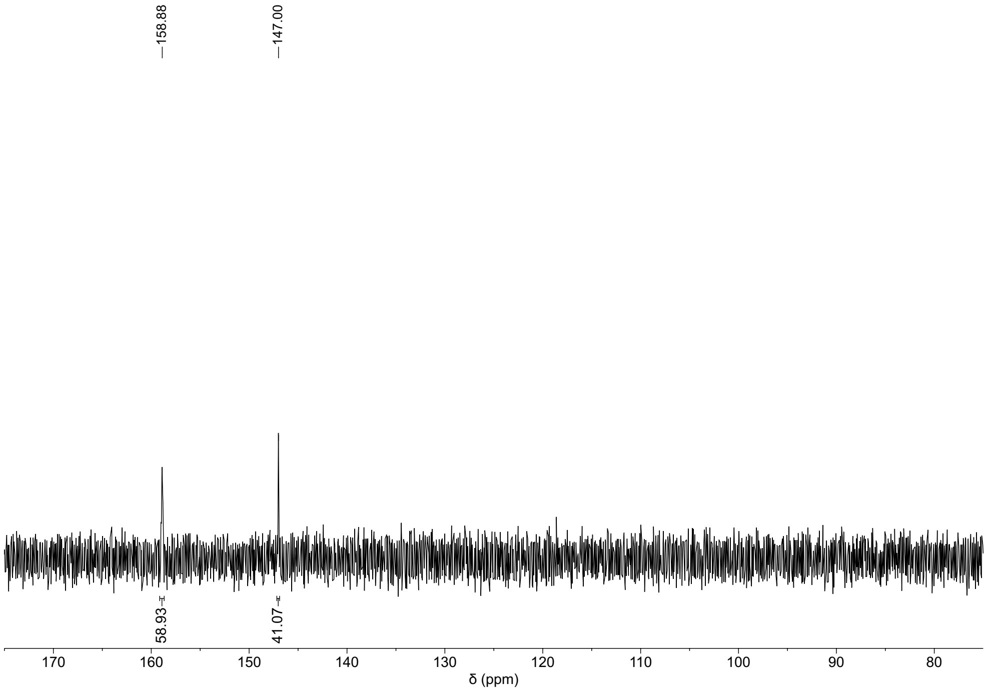

Figure S12. ^{31}P NMR spectrum of reaction **B**.

E. In situ NMR time study of stoichiometric borylation of **4d** with HBpin by **3**.

In the glovebox, to a 4 mL vial was added **3** (10.4 mg, 25 µmol, 1 equiv) and 1,2-dimethylindole (**4d**, 18.5 mg, 125 µmol, 5 equiv, 98%). CD_3CN was added (0.5 mL, 0.05 M) followed by HBpin (36.3 µL, 250 µmol, 10 equiv), and the vial was capped and removed from the glovebox. Under N_2, *N*,*N*-diisiopropylethylamine (4.3 µL, 25 µmol, 1 equiv) was added to the reaction solution, the solution was shaken, and quickly transferred to a N_2-filled screw cap NMR tube. The sample was inserted into a 60 °C thermostatted NMR probe and an initial timepoint ($^{31}P\{H\}$, ^{1}H) was immediately taken. Timepoints were then taken at the following times: 15 minutes, 30 minutes, 45 minutes, 60 minutes, 90 minutes, 120 minutes, 150 minutes, 180 minutes. Yield was assessed by ^{1}H NMR as conversion (no internal standard) by relative integration (total 500) between starting material **4d** (6.22 ppm, s, 1H) and product **5d** (7.89 ppm, d, 1H). $^{31}P\{H\}$ NMR shifts: **2** (85.0 ppm), **3** (146.94 ppm).

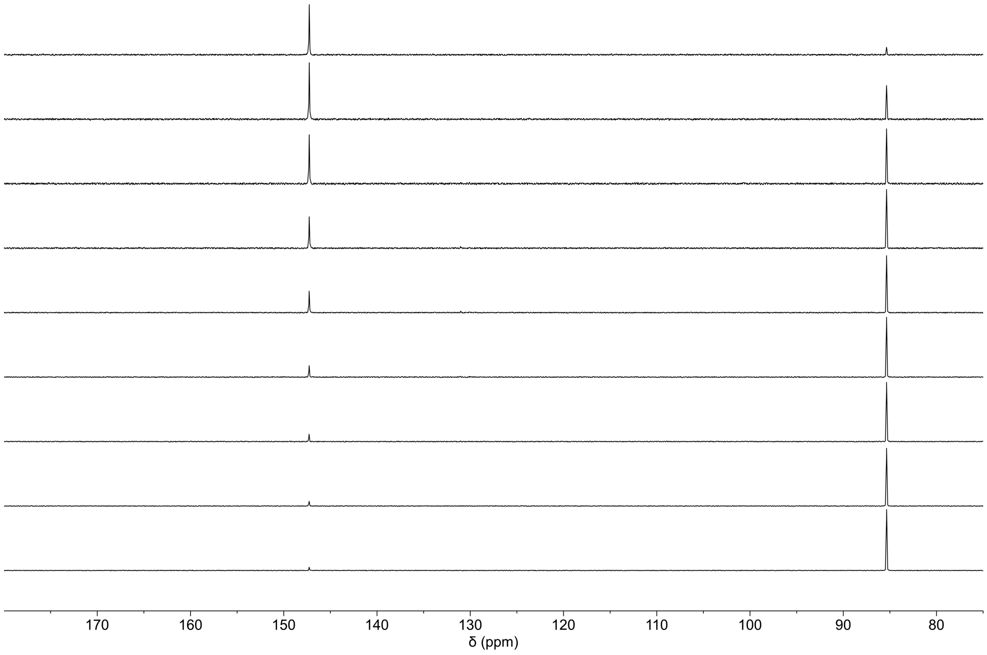

Figure S13. Time-stacked $^{31}P\{H\}$ NMR spectra of stoichiometric borylation of **4d** by **3**.

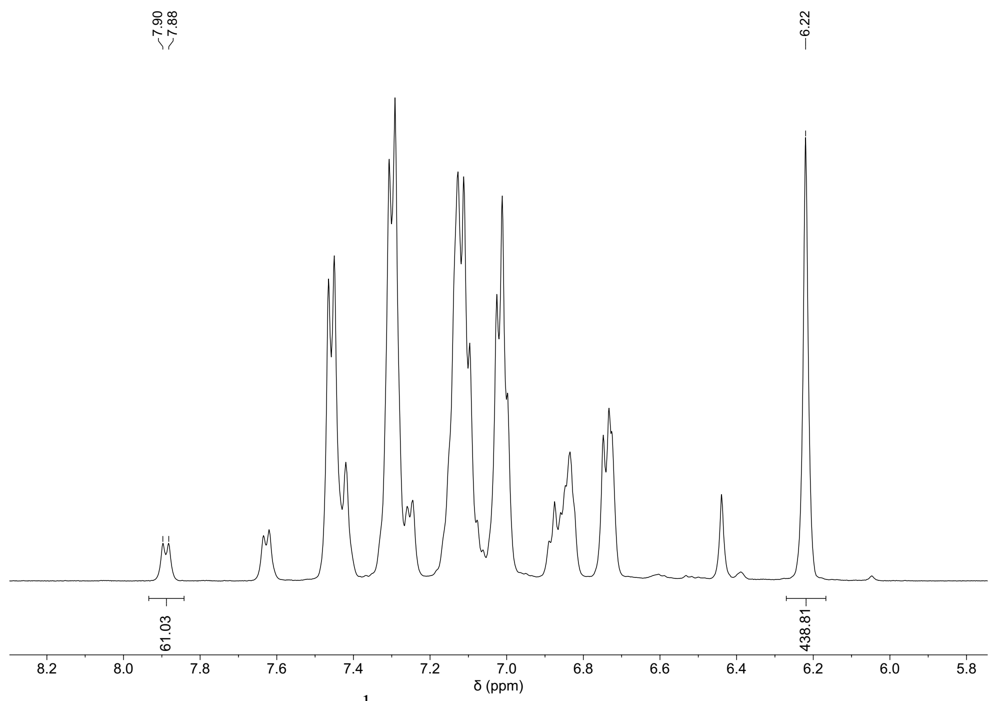

Figure S14. Representative ^{1}H NMR spectrum for yield determination at t = 2 h.

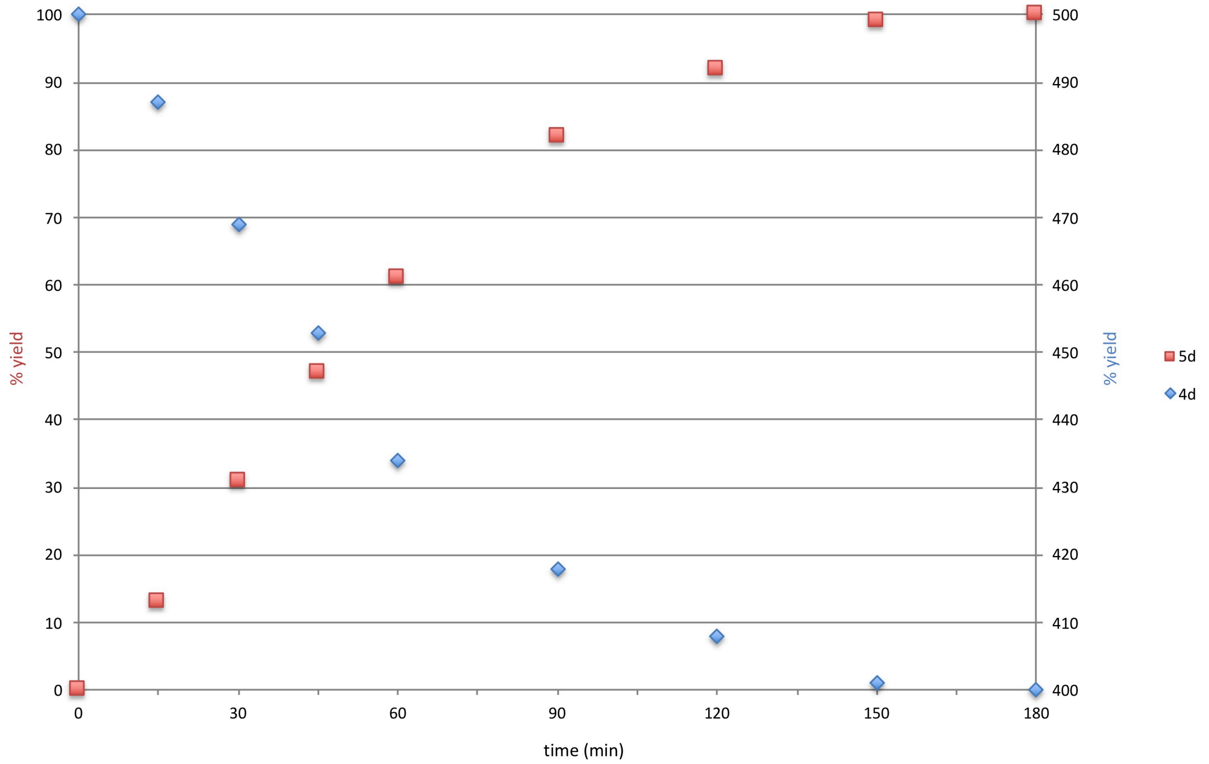

Figure S15. In situ NMR reaction progress of borylation of **4d** by **3** compared to **1**.

F. Stoichiometric borylation of **4d** with HB(pin-d_6) by **3**.

In a glovebox, a 4 mL vial equipped with magnetic stir bar was charged with **3** (8.4 mg, 20 µmol, 1 equiv) and 1,2-dimethylindole (**4d**, 14.8 mg, 100 µmol, 5 equiv, 98%). The solids were dissolved in CH_3CN (0.4 mL, 0.05 M). HB(pin-d_6) (26.8 mg, 200 µmol, 10 equiv) and then *N*,*N*-diisiopropylethylamine (3.5 µL, 20 µmol, 1 equiv) were added to the reaction solution, which was quickly capped and heated to 60 ºC for 3 h. After 3 h, the reaction was cooled to ambient temperature and 60 µL of a stock solution of 1,3,5-trimethoxybenzene (0.244 M in CH_3CN, prepared in a 2 mL volumetric flask with 82 mg 1,3,5-trimethoxybenzene, 487.5 µmol, 24.4 equiv, 99%) was added as internal standard. An aliquot was then diluted in $CDCl_3$ and analyzed by ^1H NMR showing 89% yield **5d**. The yield was determined by relative integration between 1,3,5-trimethoxybenzene (6.06 ppm, s, 3 H, 0.73 equiv, integrate to 219), starting material **4d** (6.21 ppm, s, 1H), and product **5d** (7.96 ppm, d, 1H). Number of scans = 8 and relaxation delay = 4 seconds. GCMS analysis of an aliquot diluted in CH_3CN showed a 14:86 mass count ratio of 271.30 (**5d**) to 277.30 (**5d**-d_6) averaged over 11.45-11.65 minutes.

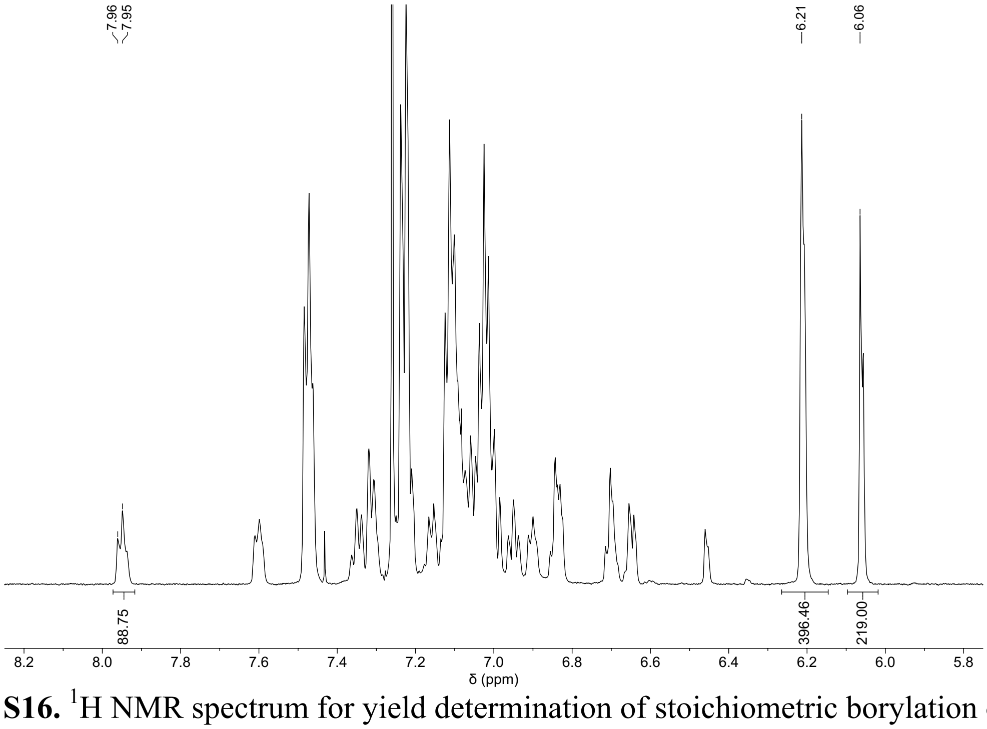

Figure S16. ^1H NMR spectrum for yield determination of stoichiometric borylation of **4d** with HB(pin-d_6) by **3**.

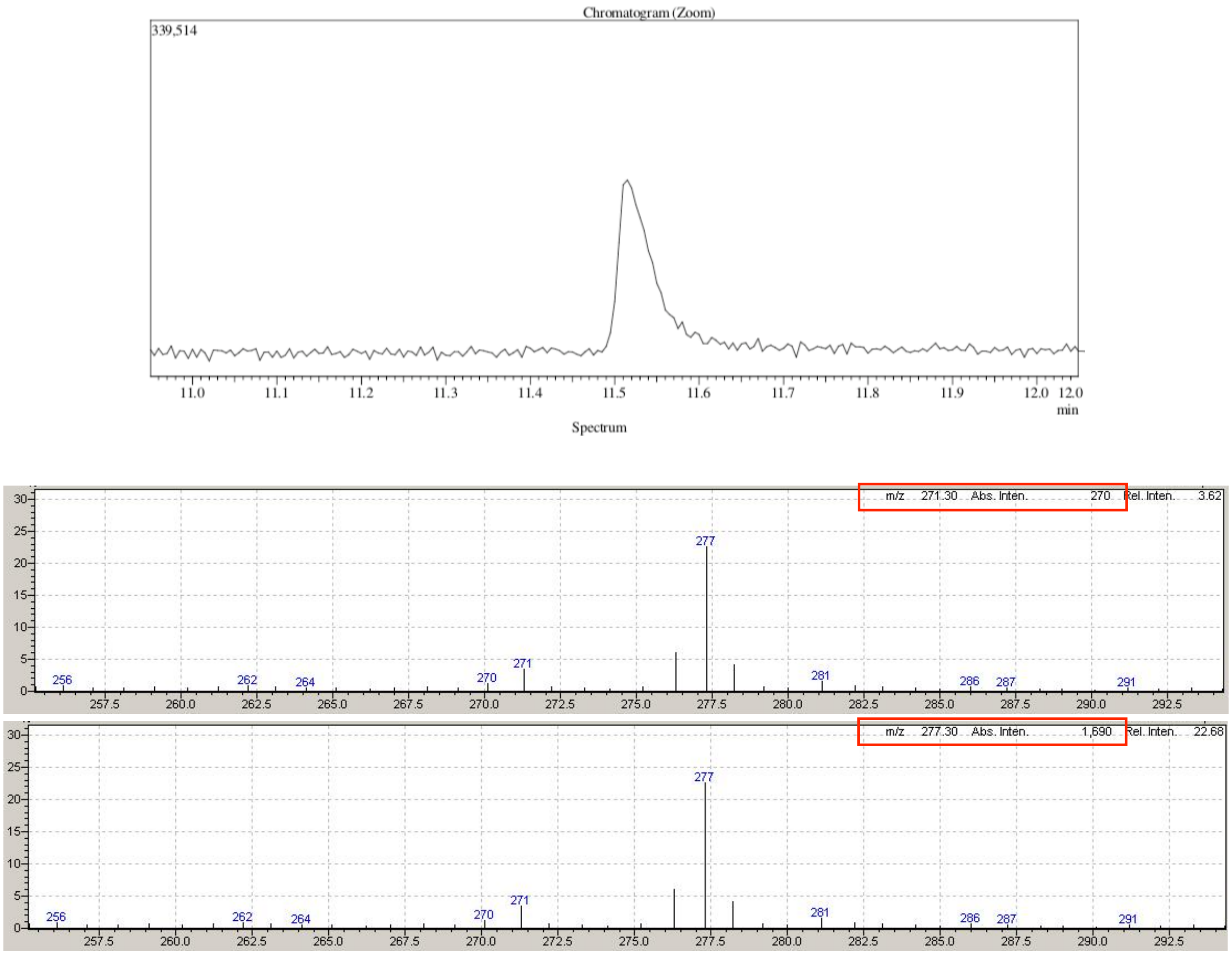

Figure S17. Zoomed GC and MS traces of stoichiometric borylation of **4d** with HB(pin-d_6) by **3**.

G. Stoichiometric borylative exchange of **5d** with HB(pin-d_6) by **3**.

In a glovebox, a 4 mL vial equipped with magnetic stir bar was charged with **3** (8.4 mg, 20 μmol, 1 equiv) and **5d** (27.1 mg, 100 μmol, 5 equiv). The solids were dissolved in CH_3CN (0.4 mL, 0.05 M). HB(pin-d_6) (26.8 mg, 200 μmol, 10 equiv) and then *N*,*N*-diisiopropylethylamine (3.5 μL, 20 μmol, 1 equiv) were added to the reaction solution, which was quickly capped and heated to 60 ºC for 3 h. After 3 h, the reaction was cooled to ambient temperature and an aliquot was diluted in CH_3CN for GCMS, which showed a 99.8:0.2 mass count ratio of 271.30 (**5d**) to 277.30 (**5d**-d_6) averaged over 11.45-11.65 minutes.

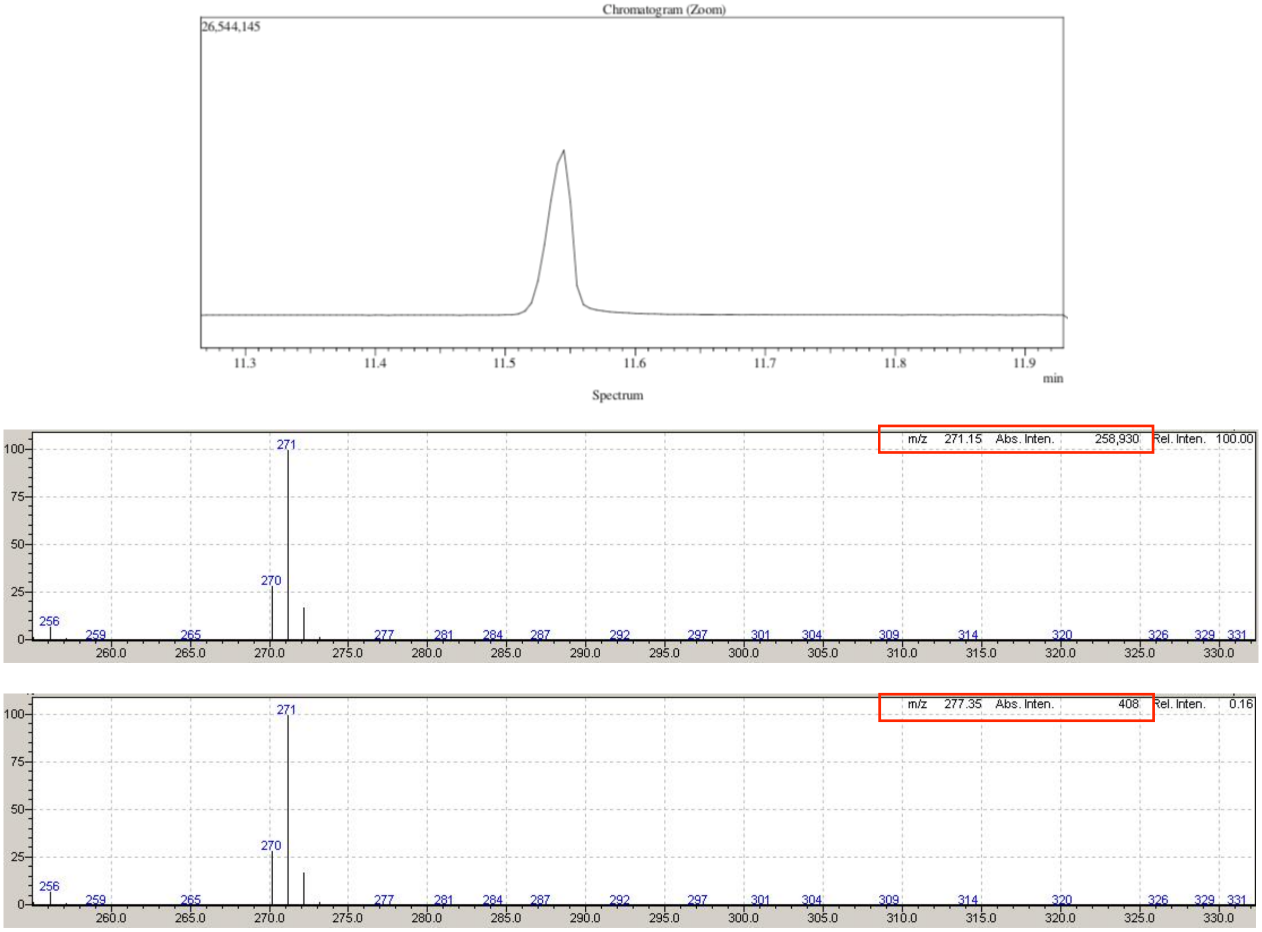

Figure S18. Zoomed GC and MS traces of stoichiometric borylation of **5d** with HB(pin-d_6) by **3**.

H. Catalytic borylative exchange of **5d** with HB(pin-d_6) by **1**.

In a glovebox, a stock solution was prepared in a 2 mL volumetric flask. The 2 mL volumetric flask was charged with **1** (25.5 mg, 100 μmol, 0.20 equiv), *N*,*N*-diisiopropylethylamine (129.3 mg, 1.0 mmol, 2 equiv), and $CHCl_3$ (119.4 mg, 1.0 mmol, 2 equiv). CH_3CN was added to reach 2 mL volume. 300 μL (0.25 M) of this stock solution was added to a 4 mL vial (reaction **A**) previously charged with **5d** (20.3 mg, 75 μmol, 1 equiv) and a magnetic stir bar. In another 4 mL vial (reaction **B**), **5d** (20.3 mg, 75 μmol, 1 equiv) was dissolved in CH_3CN (300 μL, 0.25 M). To each vial was then added HB(pin-d_6) (20.1 μL, 150 μmol, 2 equiv). The reactions were capped, removed from the glovebox, and heated in a pie block to 60 ºC for 16 h with 300 rpm stirring. After 16 h, the reactions were allowed to cool to ambient temperature, and an aliquot of each was diluted in CH_3CN for GCMS, which showed a 72:28 mass count ratio (reaction **A**) and a 99.6:0.4 mass count ratio (reaction **B**) of 271.30 (**5d**) to 277.30 (**5d**-d_6) averaged over 11.45-11.65 min.

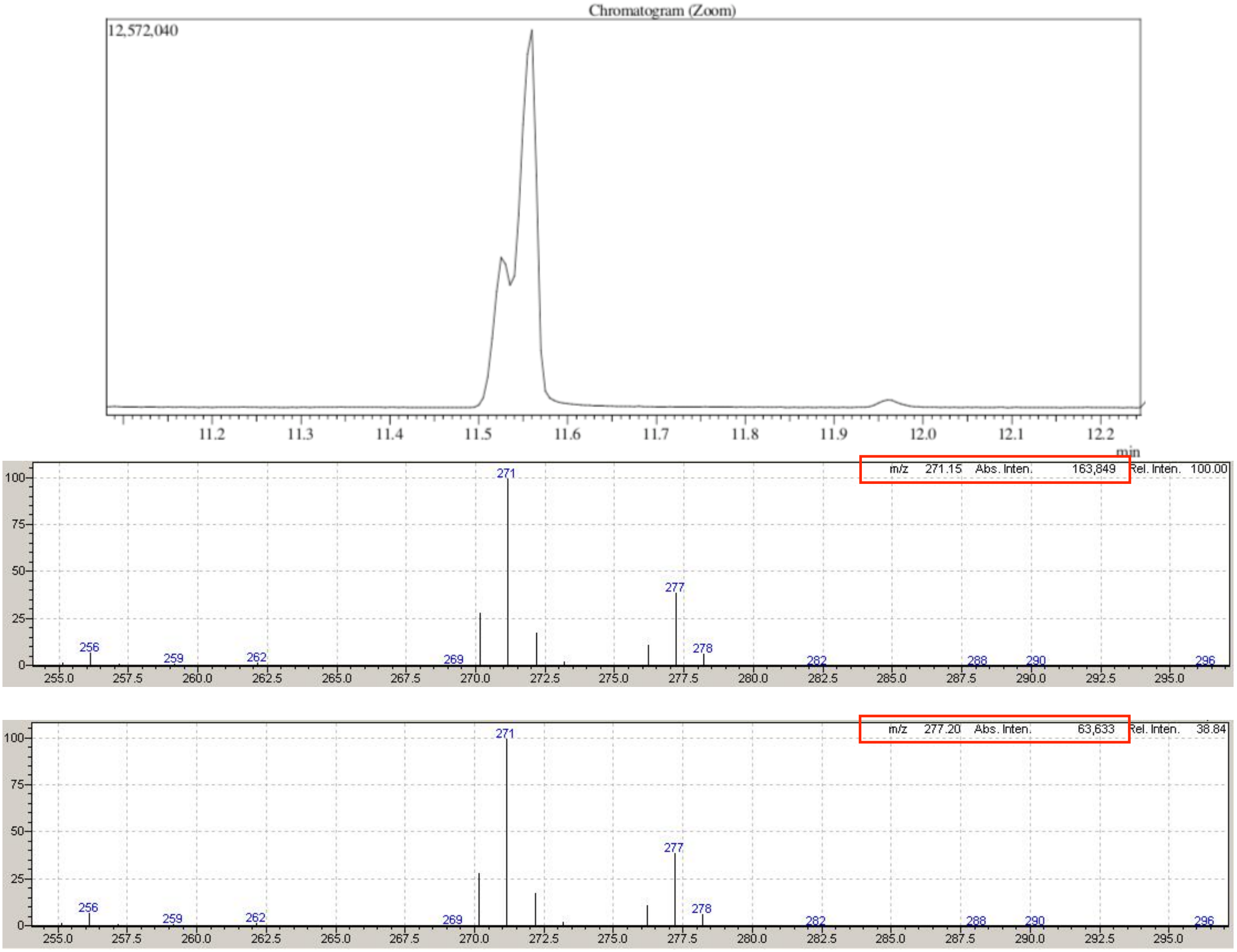

Figure S19. Zoomed GC and MS traces of borylative exchange of **5d** with HB(pin-d_6) catalyzed by **1** (reaction **A**).

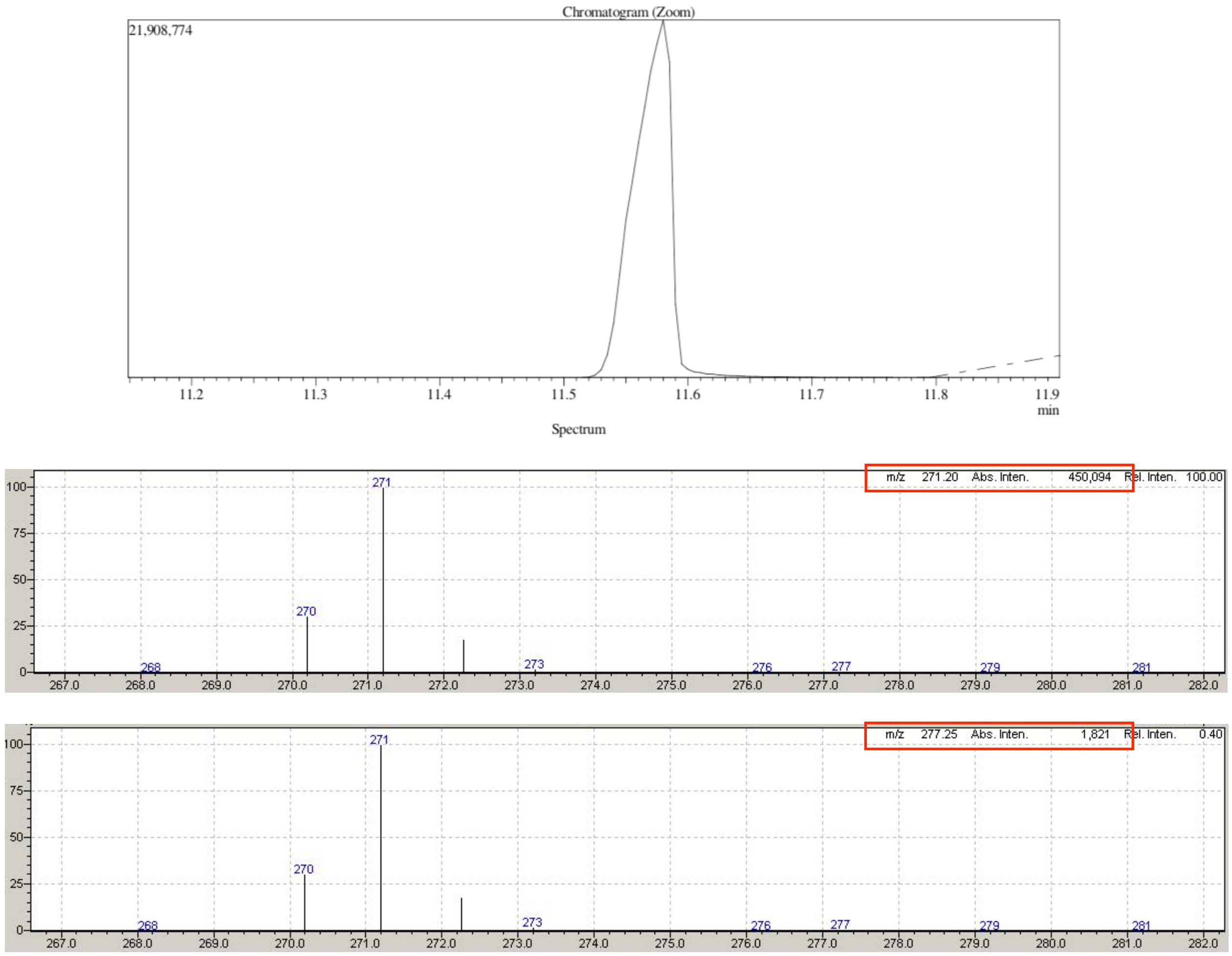

Figure S20. Zoomed GC and MS traces of borylative exchange of **5d** with HB(pin-d_6) in the absence of **1** (reaction **B**).

I. Catalytic borylation of **4d** by **7**.

In the glovebox, a stock solution was prepared in a 2 mL volumetric flask. The 2 mL volumetric flask was charged with **4d** (74.1 mg, 0.5 mmol), *N*,*N*-diisiopropylethylamine (129.3 mg, 1.0 mmol, 2 equiv), and $CHCl_3$ (119.4 mg, 1.0 mmol, 2 equiv). CH_3CN was added to reach 2 mL volume. 500 µL of this stock solution was added to three 4 mL vials previously charged with **7** (6.6 mg, 25 µmol, 0.2 equiv) and magnetic stir bar. To each vial was then added pinacolborane (36.3 µL, 0.25 mmol, 2 equiv). The reactions were capped, removed from the glovebox, and heated in a pie block to 60 ºC (reaction **A**), 80 ºC (reaction **B**), and 100 ºC (reaction **C**) for 16 h with 300 rpm stirring. After 16 h, the reactions were allowed to cool to ambient temperature, and 1,1,2,2-tetrachloroethane (20 µL, 0.186 mmol, 1.49 equiv, 98%) was added as internal standard. An aliquot was then diluted in $CDCl_3$ and analyzed by ^1H NMR. The yield was determined by relative integration between $C_2H_2Cl_4$ (5.95 ppm, s, 2 H, 1.49 equiv, integrate to 298.18), starting material **4d** (6.22 ppm, s, 1H), and product **5d** (7.96 ppm, d, 1H). Number of scans = 8 and relaxation delay = 4 seconds.

Reaction **A** – 44% **5d**, 65% **4d**. Reaction B – 65% **5d**, 43% **4d**. Reaction **C** – 81% **5d**, 25% **4d**.

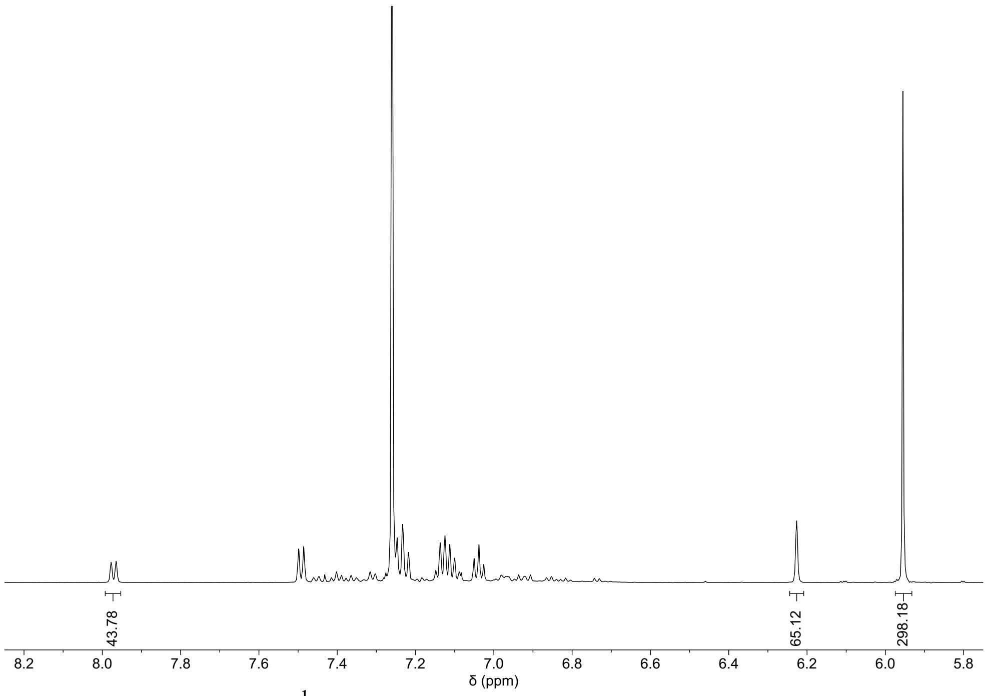

Figure S21. Representative ^1H NMR spectrum for yield determination for reaction **A**.

J. Stoichiometric borylation of **4d** with HBpin by **7**.

In the glovebox, a stock solution was prepared in a 2 mL volumetric flask. The 2 mL volumetric flask was charged with **4d** (74.1 mg, 0.5 mmol, 5 equiv). CH_3CN was added to reach 2 mL volume. 400 µL of this stock solution was added to three 4 mL vials previously charged with **7** (5.3 mg, 20 µmol, 1 equiv) and magnetic stir bar. To each vial was added pinacolborane (29.0 µL, 0.2 mmol, 2 equiv) and then *N*,*N*-diisiopropylethylamine (3.5 µL, 20 µmol, 1 equiv). The reactions were capped, removed from the glovebox, and heated in a pie block to 60 ºC (reaction **A**), 80 ºC (reaction **B**), and 100 ºC (reaction **C**) for 3 h with 300 rpm stirring. After 16 h, the reactions were allowed to cool to ambient temperature, and 50 µL of a stock solution of 1,3,5-trimethoxybenzene (0.5 M in CH_3CN, prepared in a 1 mL volumetric flask with 85 mg 1,3,5-trimethoxybenzene, 500 µmol, 20 equiv, 99%) was added as internal standard. An aliquot was then diluted in $CDCl_3$ and analyzed by ^1H NMR. The yield was determined by relative integration between 1,3,5-trimethoxybenzene (6.07 ppm, s, 3 H, 1 equiv, integrate to 300), starting material **4d** (6.22 ppm, s, 1H), and product **5d** (7.96 ppm, d, 1H). Number of scans = 8 and relaxation delay = 4 seconds.

Reaction **A** – 12% **5d**, 388% **4d**. Reaction B – 19% **5d**, 393% **4d**. Reaction **C** – 28% **5d**, 388% **4d**.

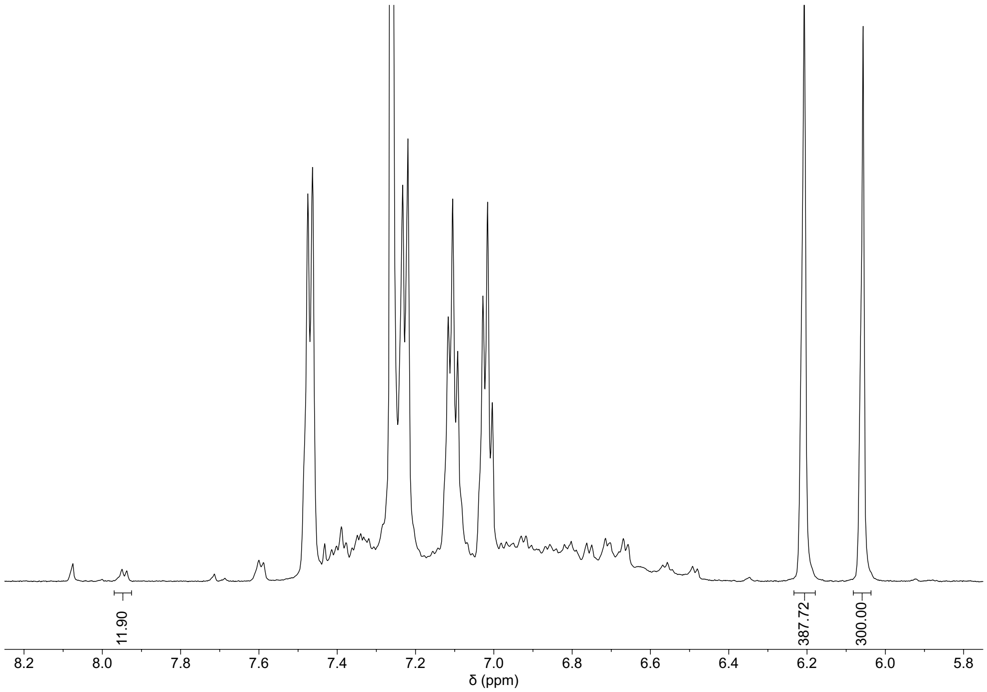

Figure S22. Representative ^1H NMR spectrum for yield determination for reaction **A**.

K. Synthesis of HB(pin-d_6).

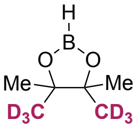

4,5-Dimethyl-4,5-bis(methyl-d_3)-1,3,2-dioxaborolane.

Prepared according to a modified literature procedure.[3] In a 50 mL pear shaped flask, 2,3-dimethylbutane-1,1,1,4,4,4-d_6-2,3-diol[4] (6.0 g, 48.3 mmol, 1 equiv) was dissolved in tetraglyme (9.6 mL, 5 M). The resultant solution was added dropwise to a water bath-cooled stirred solution of *N*,*N*-diethylaniline borane complex (12.88 mL, 72.46 mmol, 1.5 equiv) in tetraglyme (19.3 mL, 2.5 M) in a 100 mL 3-neck round bottom flask equipped with short path distillation apparatus and vent needle. The resultant solution was stirred for 30 minutes until off-gassing ceased. The vent needle was removed, and the flask was then placed in a 50 ºC oil bath. HB(pin-d_6) was then distilled under vacuum (25 torr) into an ice bath-cooled Schlenk flask. The distilled HB(pin-d_6) was thoroughly degassed by 3 cycles of freeze-pump-thaw and then brought into the glovebox and stored indefinitely in a -35 ºC freezer (1.32 g, 20 % yield).

^{1}H NMR (500 MHz, $CDCl_3$) δ 3.87 (dd, J = 344.6, 171.8 Hz, 1H), 1.26 (s, 6H).

^{2}H NMR (61 MHz, $CHCl_3$ with $CDCl_3$) δ 1.24 (s, 6H).

^{13}C NMR (126 MHz, $CDCl_3$) δ 83.26, 24.93, 24.75 – 23.83 (m).

^{11}B NMR (128 MHz, $CDCl_3$) δ 28.20 (d, J = 188.8 Hz).

HRMS (DART) calculated for $C_6H_7D_6O_2B$ $[M+H]^+$ 135.1463, found 135.1466.

V. Computational Results and Discussion

A. General computational details

All DFT calculations were carried out using the Gaussian 16 program[5] on Pitt CRC, TACC Frontera, and XSEDE[6] supercomputers. Geometries of intermediates and transition states were optimized using the M06-2X functional[7] with the 6-31G(d) basis set in acetonitrile solvent with the SMD continuum solvation model.[8] Vibrational frequency calculations were performed for all stationary points to confirm if each optimized structure is a local minimum or a transition state structure. All optimized transition state structures have only one imaginary (negative) frequency, and all minima (reactants, products, and intermediates) have no imaginary frequencies. The M06-2X functional with the 6-311+G(d,p) basis set was used for single-point energy calculations in solution. Solvation energy corrections were calculated in acetonitrile solvent with the SMD continuum solvation model based on the optimized geometries. The reported Gibbs free energies were calculated at the standard conditions (298 K, 1 atm). Truhlar's quasi-harmonic corrections[9] were applied for entropy calculations with a frequency cut-off of 100 cm^{-1} using GoodVibes v3.[10] The 3D images of computed structures were prepared using CYLView[11] and POV-Ray.

B. Computed Reaction Energy Profiles

The computed energy profiles of *P*-chloro diazaphospholene **3**-catalyzed electrophilic C–H borylation of indole **4d** with HBpin is shown in Figure S23. The most favorable reaction pathway involves a stepwise, formal σ-bond metathesis mechanism. In this pathway, complexation between **3** and HBpin first forms a Lewis acid/base pair **8**, which is 6.9 kcal/mol higher in Gibbs free energy due to disfavored entropic effects during the complexation. Subsequent chloride dissociation (**TS2**) to **6** and hydride abstraction (**TS3**) to 2 and ClBpin require activation free energies of 19.2 and 21.9 kcal/mol, respectively. Therefore, the hydride abstraction is the rate-limiting step. A similar hydride abstraction mechanism has been reported[12] where HBpin is activated by *N*-heterocyclic phosphenium triflates to form [$(Py)_2$•Bpin]OTf. The highly reactive ClBpin readily undergoes S_EAr with indole **4d** (**TS4**) to produce zwitterionic intermediate **10** with an activation energy barrier of 16.7 kcal/mol followed by exergonic elimination of HCl with *N*,*N*-diisopropylethylamine as the base (ΔG = -13.9 kcal/mol).

We also considered several less favorable alternative pathways. Direct S_EAr borylation of **4d** (**TS1**) with the pendant Bpin moiety of **3** has an activation energy barrier of 57.6 kcal/mol. The three-component, concerted B–H activation/borylation with exogenous HBpin, **3**, and **4d** (**TS5**) requires an activation energy of 37.7 kcal/mol. The formal C–H phosphorylation of **4d** (**TS6**) with phosphonium cation and chloride base pathway requires an activation energy of 29.1 kcal/mol. Replacing the chloride with $EtN^{i}Pr_2$ increases the activation energy (**TS7**) to 32.5 kcal/mol. Direct elimination from **3** to form **1** and ClBpin is thermodynamically disfavored by ΔG = 15.9 kcal/mol (c.f. ΔG = 3.4 kcal/mol for bimolecular reaction of **3** and HBpin to form **2** and ClBpin).

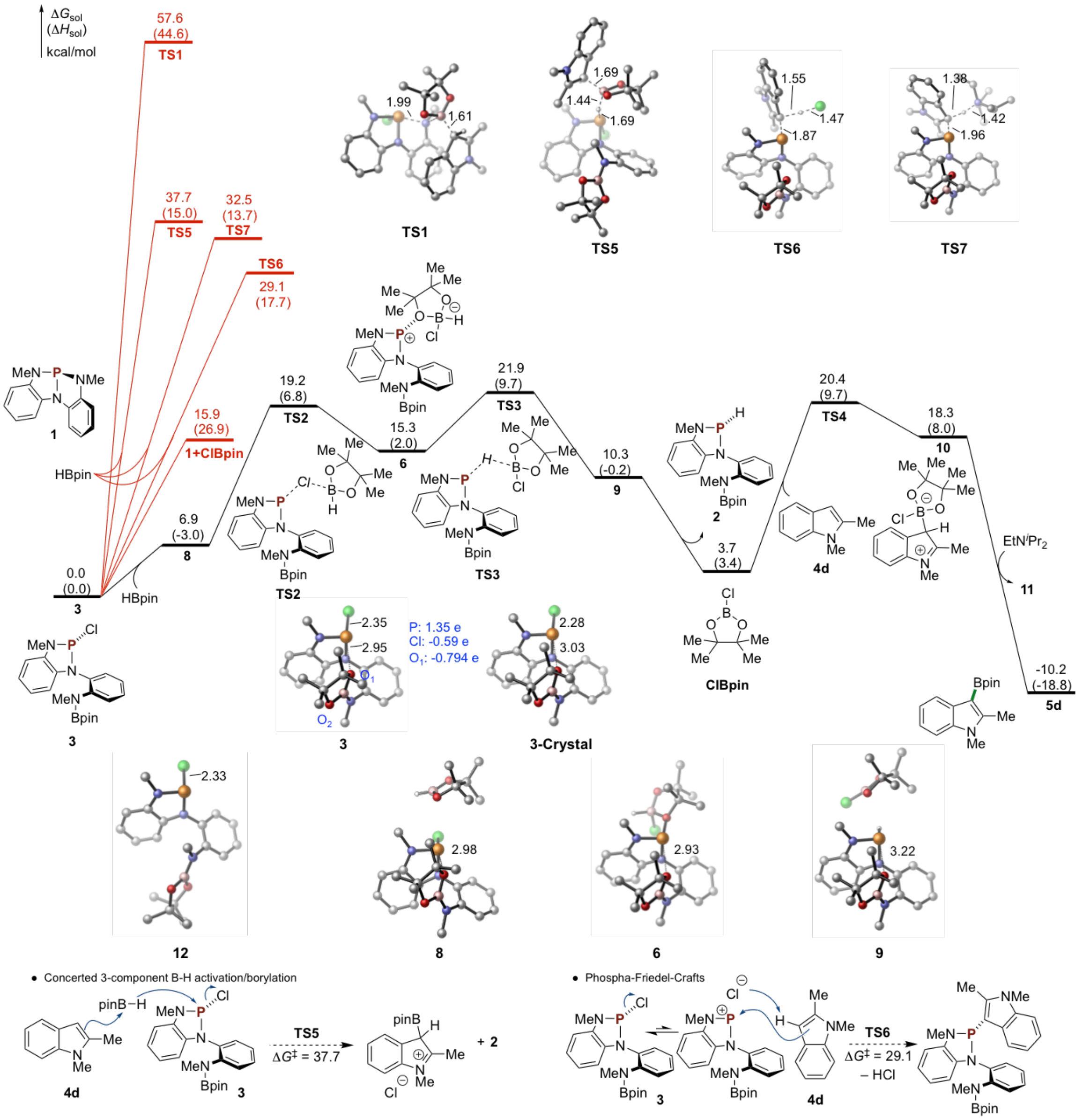

Figure S23. Energy profile of *P*-chloro diazaphospholene **3**-catalyzed electrophilic C–H borylation of **4d** with HBpin. NPA charges are indicated in blue.

C. Origin of the reactivity difference between **3** and **7**

To shed more light on the origin of the higher reactivity in the C–H borylation with **3** in comparison to that with **7**, we computed the stepwise formal σ-bond metathesis transition states between **7** and HBpin (Figure S24). The activation energies for P–Cl bond cleavage and P–H bond formation are both slightly higher in the reaction with **7** than the corresponding transition states in the reaction with **3**. These results suggest that the weak P–O coordination in **3**[13] does not block the approach of HBpin due to steric effects. On the other hand, the P–O coordination slightly stabilizes both transition states, potentially due to the stabilization of the positive charge building up on the P compared to the ground state **3**. In the course of the reaction of **3** and HBpin to form **2** and ClBpin, the distance between an *O*-atom of the Bpin moiety of **3** and the electropositive *P*-atom becomes shorter in the rate-limiting TS (d(P-O) = 2.95 Å and 2.90 Å in **3** and **TS3**, respectively), indicating the formation of ClBpin is promoted due to the P–O interaction that stabilizes the transition state.

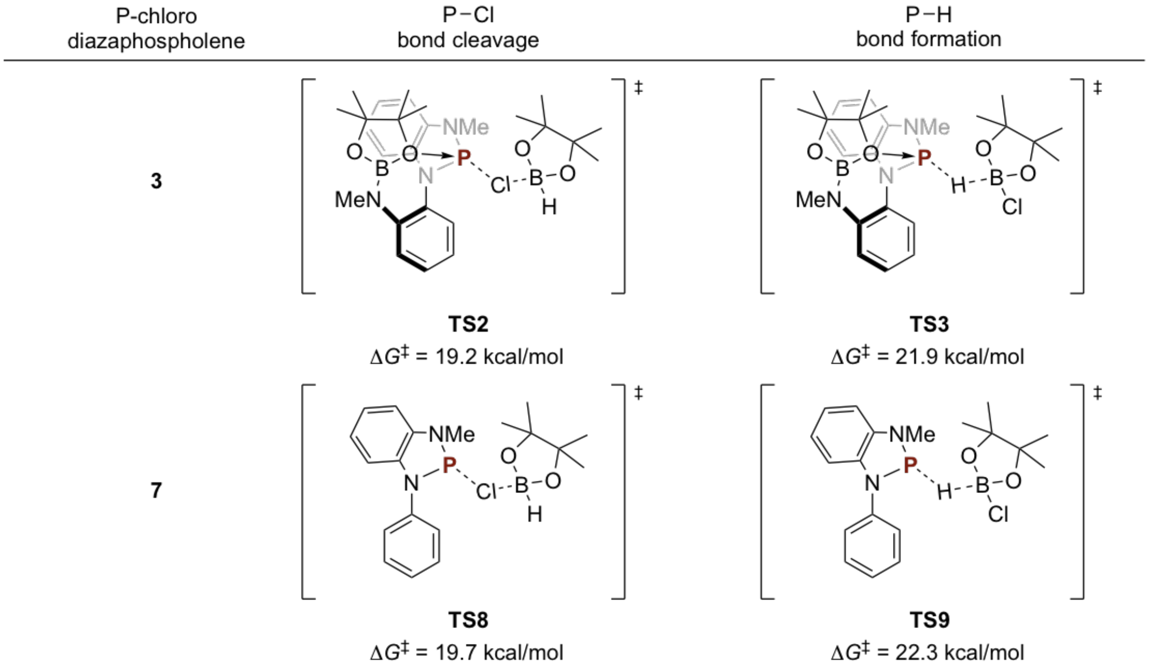

Figure S24. Transition state structures of stepwise σ-bond metathesis with **3** and **7**

To investigate the magnitude of the stabilizing interaction between the oxygen on pendant borylamine and the P center, we located conformer **12** where the oxygen is pointing away from the P center. Indeed, without weak stabilizing interaction between P and the oxygen, **12** is 3.3 kcal/mol higher in energy than **3**. In addition to the relatively moderate stabilization of transition

states **TS2** and **TS3**, the weak P–O coordination with the *N*-boryl substituent in **3** may also prevent the P center from coordinating to Lewis basic species (e.g. *N*,*N*-diisopropylethylamine) and forming stable off-cycle complexes that diminish its reactivity.

We also performed geometry optimizations and single point energy calculations at different levels of theory to evaluate the effects of density functional and basis set on reactivity difference between **3** and **7**. The computed activation energy barriers of the rate-limiting P-H bond formation step are shown in Table S1. All but one computational methods tested suggest that the activation energy of reaction with **3** (**TS3**) is slightly lower than that with **7** (**TS9**).

- Geometry optimization at M062x/6-31g(d) level of theory

DFT methods for SP	$\Delta G_{TS3}^{‡}$	$\Delta G_{TS9}^{‡}$	$\Delta\Delta G^{‡}$
M062X/6-311+G(d,p)	21.9	22.3	-0.4
M062X-D3/6-311+G(d,p)	20.4	21.1	-0.7
wB97xD/6-311++G(d,p)	22.1	22.9	-0.8
wB97xD/6-311+G(d,p)	22.1	22.9	-0.8

- Geometry optimization at M062x/6-31+g(d) level of theory

DFT methods for SP	$\Delta G_{TS3}^{‡}$	$\Delta G_{TS9}^{‡}$	$\Delta\Delta G^{‡}$
M062X/6-311+G(d,p)	20.7	20.8	-0.1
M062X-D3/6-311+G(d,p)	22.2	22.0	0.2
wB97xD/6-311++G(d,p)	22.1	22.3	-0.2
wB97xD/6-311+G(d,p)	22.1	22.3	-0.2

Table S1. Comparison of activation free energies with different functionals and basis sets.[a]

[a]All energies are in kcal/mol.

VI. Synthesis of Phosphorus Compounds

A. Synthesis of **1**

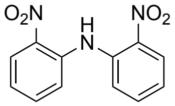

Bis(2-nitrophenyl)amine (S1).

Synthesized according to literature procedure.[14] 1-Fluoro-2-nitrobenzene (10.55 mL, 100 mmol, 1 equiv) was added to a solution of 2-nitroaniline (13.81 g, 100 mmol, 1 equiv) and potassium carbonate (16.58 g, 120 mmol, 1.2 equiv) in DMSO (285 mL, 0.35 M) in a 500 mL round bottom flask with magnetic stir bar. The reaction mixture was heated with stirring at 120 ºC for 72 h. The reaction mixture was cooled and H_2O (300 mL) was added to generate an orange precipitate. The solid was collected by vacuum filtration over filter paper, washed with H_2O (800 mL), and dried under vacuum with heating in a 50 ºC oil bath overnight to give the product as an orange solid (25.22 g, 97%). The product was used in the next step without further purification. The product was identical to literature reports.

^{1}H NMR (400 MHz, $CDCl_3$) δ 11.01 (bs, 1H), 8.21 (dd, J = 8.4, 1.6 Hz, 2H), 7.59 (dd, J = 8.4, 1.4 Hz, 2H), 7.53 (ddd, J = 8.4, 6.9, 1.5 Hz, 2H), 7.10 (ddd, J = 8.5, 7.0, 1.4 Hz, 2H).

^{13}C NMR (151 MHz, $CDCl_3$) δ 138.78, 137.40, 134.95, 126.94, 122.03, 119.94.

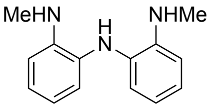

Bis(2-methylaminophenyl)amine (S2).

Synthesized according to modified literature procedure.[14,15] A 1 L 2-neck round bottom flask was charged with magnetic stir bar, **S1** (25.15 g, 97 mmol, 1 equiv), zinc powder (88.8 g, 1.36 mol, 14 equiv), and NH_4Cl (67.5 g, 1.26 mol, 13 equiv). The flask was equipped with a reflux condenser and evacuated and backfilled with N_2 3x. The reaction components were suspended in THF (485 mL, 0.2 M) and heated to reflux for 14 hours. The resultant gray suspension was cooled to ambient temperature and filtered quickly through celite, washing with Et_2O (400 mL). The filtrate was concentrated under reduced pressure in a 1 L flask, and to the resultant dark amorphous gel was added a magnetic stir bar, MeOH (485 mL, 0.2 M), and paraformaldehyde (28.69 g, 956 mmol, 9.85 equiv). The resultant suspension was then cooled to 0 ºC, and then sodium methoxide (25% in MeOH, 7.80 mL) was added slowly. The reaction mixture was then heated to reflux for 1 h. The solution was then cooled to ambient temperature and then 0 ºC. Sodium borohydride (36.7 g, 970 mmol, 10 equiv) was then added slowly in six portions. The reaction mixture was subsequently heated to reflux and stirred for 90 minutes. The mixture was cooled to ambient temperature and transferred to a 2 L flask. Aqueous sodium hydroxide (550 mL, 1 M) was added to precipitate a solid. The flask was stored in a –20 ºC freezer for 3 hours, and then the solid was collected by vacuum filtration over filter paper. The solid was washed with H_2O (500 mL), collected in a 100 mL flask, and dried under vacuum overnight at 40 ºC to afford the product as a purple solid (19.98 g, 91% over two steps). The product was identical to literature reports.

^{1}H NMR (600 MHz, $CDCl_3$) δ 7.03 (ddd, J = 8.4, 6.7, 2.2 Hz, 2H), 6.73 (d, J = 7.9 Hz, 2H), 6.73 – 6.66 (m, 4H), 4.86 (s, 1H), 3.75 (d, J = 5.6 Hz, 2H), 2.87 (d, J = 5.4 Hz, 6H).

^{13}C NMR (151 MHz, $CDCl_3$) δ 141.92, 130.86, 123.81, 119.84, 118.03, 110.82, 30.90.

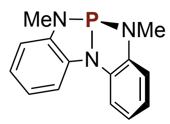

5,7-Dimethyl-5*H*,7*H*-benzo[*d*]benzo[4,5][1,3,2]diazaphospholo[1,2-*a*][1,3,2]diazaphosphole (1).

Synthesized according to modified literature procedure.[14] Phosphorus trichloride (1.92 mL, 22 mmol, 1 equiv) was added to Et_2O (67 mL, 0.33 M) in an oven-dried, N_2 filled 500 mL Schlenk flask and cooled to -78 ºC. In an oven-dried 50 mL pear-shaped flask, **S2** (5 g, 22 mmol, 1 equiv) was dissolved in THF (17 mL, 1.3 M) and then added dropwise to the reaction solution via syringe. In an oven-dried 100 mL pear-shaped flask, NEt_3 (15.5 mL, 111 mmol, 5.05 equiv) in Et_2O (50 mL) was added dropwise to the reaction solution via cannula transfer. The reaction was stirred at -78 ºC for 1 h, and then warmed to ambient temperature and stirred for 4 h. The volatiles were removed under vacuum, and the resulting solid residue was brought into a nitrogen filled glovebox. The solid was suspended in pentane and filtered through celite. The filtrate was evaporated to give a slightly pink solid, which was dissolved in minimal Et_2O and cooled to -35 ºC to induce crystallization of **1** as colorless crystals (4 cycles, 3.52 g, 63%). The product was identical to literature reports.

^{1}H NMR (500 MHz, $CDCl_3$). δ 7.41 (d, J = 7.7 Hz, 2H), 7.03 – 6.97 (m, 2H), 6.85 – 6.78 (m, 2H), 6.59 (d, J = 7.6 Hz, 2H), 3.06 (d, J = 8.4 Hz, 6H).

^{13}C{H} NMR (101 MHz, $CDCl_3$). δ 141.27 (d, J = 7.2 Hz), 139.63, 124.40, 118.97, 117.13 (d, J = 6.1 Hz), 108.68, 29.86 (d, J = 30.7 Hz).

^{31}P NMR (162 MHz, $CDCl_3$) δ 159.82.

B. Synthesis of **3**

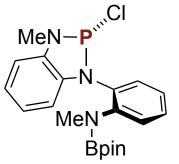

***N*-(2-(2-Chloro-3-methyl-2,3-dihydro-1*H*-benzo[*d*][1,3,2]diazaphosphol-1-yl)phenyl)-*N*,4,4,5,5-pentamethyl-1,3,2-dioxaborolan-2-amine (3)**.

Synthesized according to a modified literature procedure.[13] In a glovebox, a stirred solution of **1** (250 mg, 0.98 mmol, 1 equiv) in benzene (3.92 mL, 0.25 M) was treated with pinacolborane (142 μL, 0.98 mmol, 1 equiv). The reaction was heated to 50 ºC for 16 h until complete consumption of **1** was evident by ^{31}P NMR. The reaction was cooled to ambient temperature and $CHCl_3$ (156 μL, 1.96 mmol, 2 equiv) was added. The reaction was stirred at ambient temperature for 24 h until complete consumption of **2** was evident by ^{31}P NMR. The volatiles were removed under vacuum, pentane (1 mL) was added to the sticky residue, and the volatiles were again removed under vacuum. The remaining solid was taken up in minimal pentane (~ 35 mL), filtered thru celite, and cooled to -35 ºC to induce crystallization of **3** as colorless crystals (242 mg, 59% yield). The product was identical to literature reports.

^{1}H NMR (400 MHz, $CDCl_3$). δ 7.64 (d, J = 7.9 Hz, 1H), 7.42 – 7.30 (m, 2H), 7.26 (td, J = 7.4, 2.1 Hz, 1H), 7.12 – 7.05 (m, 1H), 7.04 – 6.94 (m, 2H), 6.87 (dd, J = 7.9, 1.2 Hz, 1H), 3.39 (d, J = 14.3 Hz, 3H), 2.85 (s, 3H), 0.94 (bs, 12H).

^{13}C{H} NMR (101 MHz, $CDCl_3$). δ 145.13 (d, J = 4.5 Hz), 137.05 (d, J = 9.1 Hz), 132.12 (d, J = 10.0 Hz), 129.61 (d, J = 6.2 Hz), 128.93 (d, J = 1.9 Hz), 128.44, 125.84 (d, J = 1.7 Hz), 121.65, 121.18, 112.07, 110.13, 82.87, 37.35 (d, J = 3.2 Hz), 29.58 (d, J = 18.0 Hz), 24.28.

^{31}P NMR (162 MHz, $CDCl_3$) δ 146.39 (q, J = 14.4 Hz).

C. Synthesis of **7**

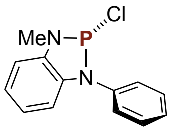

2-Chloro-1-methyl-3-phenyl-2,3-dihydro-1*H*-benzo[*d*][1,3,2]diazaphosphole (6).

Synthesized according to modified literature procedure.[12] To an oven-dried 100 mL Schlenk flask was added *N*[1]-methyl-*N*[2]-phenylbenzene-1,2-diamine (740 mg, 3.73 mmol, 1 equiv) and a magnetic stir bar. The flask was evacuated and backfilled with N_2 3x. The solid was dissolved in toluene (18.7 mL, 0.2 M), NEt_3 (1.56 mL, 11.2 mmol, 3 equiv) was added, and the solution was cooled to 0 ºC. Phosphorus trichloride (326 µL, 3.73 mmol, 1 equiv) was added to the solution dropwise. After five minutes, the reaction was allowed to warm to ambient temperature and stirred for 6 h. The volatiles were removed under vacuum, and the resulting solid residue was brought into a nitrogen filled glovebox. The solid was taken up in toluene (25 mL) and filtered through celite, washing with toluene (20 mL). The filtrate was concentrated under vacuum to approximately 10 mL volume, and pentane was added (20 mL). The resultant suspension was filtered through celite, and the filtrate was concentrated under vacuum, yielding **7** as a yellow solid (940.2 mg, 96% yield). Analytically pure material was obtained by taking up the solid in minimal Et_2O, filtering through celite, and cooling to -35 ºC to induce precipitation of **7** as a yellow amorphous crystalline solid (500 mg, 51% yield).

^{1}H NMR (400 MHz, $CDCl_3$) δ 7.57 (d, J = 7.4 Hz, 1H), 7.51 (t, J = 7.7 Hz, 1H), 7.41 (t, J = 6.9 Hz, 1H), 7.13 (ddd, J = 8.2, 6.6, 1.9 Hz, 1H), 7.08 – 6.97 (m, 2H), 3.41 (d, J = 14.6 Hz, 3H).

^{13}C{H} NMR (101 MHz, $CDCl_3$) δ 137.64 (d, J = 11.1 Hz), 137.06 (d, J = 13.1 Hz), 136.75 (d, J = 8.2 Hz), 130.01, 127.86 (d, J = 1.6 Hz), 126.78 (d, J = 6.9 Hz), 122.08, 121.30, 111.67, 110.57 (d, J = 1.5 Hz), 29.53 (d, J = 18.1 Hz).

^{31}P NMR (203 MHz, $CDCl_3$) δ 143.06 (q, J = 14.6 Hz).

HRMS (ESI) calculated for $C_{13}H_{12}N_2P$ $[M-Cl]^+$ 227.0733, found 227.0731.

VII. References

[1] X. H. Xu, G. K. Liu, A. Azuma, E. Tokunaga and N. Shibata, *Org. Lett.* **2011**, *13*, 4854–4857.

[2] B. S. Lane, M. A. Brown and D. Sames, *J. Am. Chem. Soc.* **2005**, *127*, 8050–8057.

[3] T. Kikuchi, Y. Nobuta, J. Umeda, Y. Yamamoto, T. Ishiyama and N. Miyaura, *Tetrahedron* **2008**, *64*, 4967–4971.

[4] P. J. Unsworth, D. Leonori and V. K. Aggarwal, *Angew. Chem. Int. Ed.* **2014**, *53*, 9846–9850.

[5] M. J. Frisch, G. W. Trucks, H. B. Schlegel, G. E. Scuseria, M. A. Robb, J. R. Cheeseman, G. Scalmani, V. Barone, G. A. Petersson, H. Nakatsuji, X. Li, M. Caricato, A. V. Marenich, J. Bloino, B. G. Janesko, R. Gomperts, B. Mennucci, H. P. Hratchian, J. V. Ortiz, A. F. Izmaylov, J. L. Sonnenberg, D. Williams-Young, F. Ding, F. Lipparini, F. Egidi, J. Goings, B. Peng, A. Petrone, T. Henderson, D. Ranasinghe, V. G. Zakrzewski, J. Gao, N. Rega, G. Zheng, W. Liang, M. Hada, M. Ehara, K. Toyota, R. Fukuda, J. Hasegawa, M. Ishida, T. Nakajima, Y. Honda, O. Kitao, H. Nakai, T. Vreven, K. Throssell, J. A. Montgomery, Jr., J. E. Peralta, F. Ogliaro, M. J. Bearpark, J. J. Heyd, E. N. Brothers, K. N. Kudin, V. N. Staroverov, T. A. Keith, R. Kobayashi, J. Normand, K. Raghavachari, A. P. Rendell, J. C. Burant, S. S. Iyengar, J. Tomasi, M. Cossi, J. M. Millam, M. Klene, C. Adamo, R. Cammi, J. W. Ochterski, R. L. Martin, K. Morokuma, O. Farkas, J. B. Foresman and D. J. Fox, *Gaussian 16 Rev. B.01*, Gaussian, Inc., Wallingford, CT, 2016.

[6] J. Towns, T. Cockerill, M. Dahan, I. Foster, K. Gaither, A. Grimshaw, V. Hazlewood, S. Lathrop, D. Lifka, G. D. Peterson, R. Roskies, J. R. Scott and N. Wilkins-Diehr, *Comput. Sci. Eng.* **2014**, *16*, 62−74.

[7] Y. Zhao and D. G. Truhlar, *Theor. Chem. Acc.* **2007**, *120*, 215−241.

[8] A. V. Marenich, C. J. Cramer and D. G. Truhlar, *J. Phys. Chem. B* **2009**, *113*, 6378-6396.

[9] R. F. Ribeiro, A. V. Marenich, C. J. Cramer and D. G. Truhlar, D. G. *J. Phys. Chem. B* **2011**, *115*, 14556-14562.

[10] G. Luchini, J. V. Alegre-Requena, I. Fuenes-Ardoiz and R. S. Paton, F1000 Research, **2020**, *9*, 291 DOI: 10.12688/f1000research.22758.1.

[11] CYLview, 1.0b; C. Y. Legault, Université de Sherbrooke, **2009** (http://www.cylview.org).

[12] B. Rao, C. C. Chong and R. Kinjo, *J. Am. Chem. Soc.* **2018**, *140*, 652–656.

[13] Y. C. Lin, E. Hatzakis, S. M. McCarthy, K. D. Reichl, T. Y. Lai, H. P. Yennawar and A. T. Radosevich, *J. Am. Chem. Soc.* **2017**, *139*, 6008–6016.

[14] G. T. Cleveland and A. T. Radosevich, *Angew. Chem. Int. Ed.* **2019**, *58*, 15005–15009.

[15] R. F. Munhá, R. A. Zarkesh and A. F. Heyduk, *Inorg. Chem.* **2013**, *52*, 11244–11255.

VIII. Cartesian Coordinates and Energies of Optimized Structures

Cartesian Coordinates (Å) and Energies of Optimized Structures

ClBpin

M06-2X/6-31G(d) SCF energy in solution:	-871.31222677 a.u.
M06-2X/6-31G(d) enthalpy in solution:	-871.115642 a.u.
M06-2X/6-31G(d) free energy in solution:	-871.162361 a.u.
M06-2X/6-311+G(d,p) SCF energy in solution:	-871.47262692 a.u.
M06-2X/6-311+G(d,p) enthalpy in solution:	-871.276042 a.u.
M06-2X/6-311+G(d,p) free energy in solution:	-871.322761 a.u.

Cartesian coordinates

ATOM	X	Y	Z
O	-0.499816	1.063722	-0.432680
O	-0.500008	-1.063385	0.433035
C	0.882740	0.780398	-0.053678
C	1.805658	1.342800	-1.118086
H	1.541192	0.977998	-2.112953
H	2.841387	1.061275	-0.901869
H	1.740487	2.434873	-1.119241
C	0.882402	-0.780528	0.053883
C	1.115634	1.470073	1.285448
H	0.905059	2.537673	1.175683
H	2.153195	1.352140	1.610680
H	0.456829	1.065138	2.060761
C	1.115133	-1.469751	-1.285528
H	0.904221	-2.537328	-1.176161
H	2.152737	-1.352002	-1.610700

H	0.456463	-1.064280	-2.060680
C	1.805694	-1.343433	1.117750
H	1.541352	-0.979253	2.112866
H	2.841350	-1.061700	0.901475
H	1.740656	-2.435520	1.118327
B	-1.217686	0.000321	0.000240
Cl	-2.990087	-0.000039	-0.000058

EtN^{i}Pr$_2$

M06-2X/6-31G(d) SCF energy in solution:	-370.84540417 a.u.
M06-2X/6-31G(d) enthalpy in solution:	-370.567302 a.u.
M06-2X/6-31G(d) free energy in solution:	-370.615557 a.u.
M06-2X/6-311+G(d,p) SCF energy in solution:	-370.95748060 a.u.
M06-2X/6-311+G(d,p) enthalpy in solution:	-370.679378 a.u.
M06-2X/6-311+G(d,p) free energy in solution:	-370.727633 a.u.

Cartesian coordinates

ATOM	X	Y	Z
N	-0.042818	0.138844	-0.148941
C	1.116514	-0.763253	-0.266197
H	0.822742	-1.549761	-0.967454
C	1.529763	-1.439402	1.046891
H	0.696276	-1.996547	1.488574
H	2.348933	-2.144901	0.870576
H	1.875925	-0.705753	1.783810
C	2.300073	-0.042338	-0.910842
H	3.098893	-0.759950	-1.124863
H	1.996535	0.426798	-1.852338
H	2.720091	0.731730	-0.259401
C	-1.303781	-0.517398	0.238722
H	-1.297556	-0.754026	1.319848
C	-2.478343	0.426807	-0.027384
H	-3.417404	-0.046150	0.277844
H	-2.389837	1.370657	0.517887
H	-2.536019	0.654829	-1.098232
C	-1.543743	-1.818596	-0.525712

H	-1.458319	-1.647782	-1.605452
H	-0.848733	-2.614111	-0.243581
H	-2.556660	-2.176759	-0.317502
C	0.230883	1.327393	0.675872
H	-0.475263	1.372747	1.515270
H	1.222146	1.251236	1.135699
C	0.159812	2.621267	-0.129059
H	-0.826136	2.743328	-0.588905
H	0.352570	3.491050	0.509077
H	0.904472	2.614577	-0.932014

HBpin

M06-2X/6-31G(d) SCF energy in solution:	-411.69061261 a.u.
M06-2X/6-31G(d) enthalpy in solution:	-411.487389 a.u.
M06-2X/6-31G(d) free energy in solution:	-411.530497 a.u.
M06-2X/6-311+G(d,p) SCF energy in solution:	-411.81570938 a.u.
M06-2X/6-311+G(d,p) enthalpy in solution:	-411.612486 a.u.
M06-2X/6-311+G(d,p) free energy in solution:	-411.655594 a.u.

Cartesian coordinates

ATOM	X	Y	Z
O	1.064715	1.189874	-0.419430
O	-1.064408	1.190302	0.419097
C	0.779493	-0.187277	-0.054957
C	1.341578	-1.108167	-1.123687
H	0.978124	-0.838613	-2.117850
H	1.061147	-2.145896	-0.914786
H	2.433957	-1.043161	-1.125701
C	-0.779555	-0.186992	0.055010
C	1.470340	-0.438211	1.282240
H	2.538483	-0.228303	1.173901
H	1.351599	-1.478272	1.600007
H	1.068386	0.215417	2.063351
C	-1.470448	-0.438251	-1.282116
H	-2.538513	-0.227892	-1.173865
H	-1.352063	-1.478506	-1.599407
H	-1.068283	0.214874	-2.063534
C	-1.341934	-1.107507	1.123941
H	-0.978566	-0.837728	2.118076

H	-1.061638	-2.145348	0.915385
H	-2.434297	-1.042335	1.125809
B	0.000333	1.933966	-0.000214
H	0.000700	3.122956	-0.000246

1

M06-2X/6-31G(d) SCF energy in solution: -1047.22500692 a.u.

M06-2X/6-31G(d) enthalpy in solution: -1046.950114 a.u.

M06-2X/6-31G(d) free energy in solution: -1047.008407 a.u.

M06-2X/6-311+G(d,p) SCF energy in solution: -1047.44672368 a.u.

M06-2X/6-311+G(d,p) enthalpy in solution: -1047.171831 a.u.

M06-2X/6-311+G(d,p) free energy in solution: -1047.230124 a.u.

Cartesian coordinates

ATOM	X	Y	Z
C	1.137990	-0.829784	-0.406549
C	1.415015	-2.182767	-0.294092
H	0.727427	-2.923001	-0.687273
C	2.602090	-2.581588	0.334877
H	2.822640	-3.639998	0.428930
C	3.495278	-1.636145	0.827571
H	4.415942	-1.956187	1.305530
C	3.222371	-0.268231	0.713866
H	3.912697	0.473174	1.103626
C	2.041923	0.124149	0.093732
C	-1.287531	-0.672766	-0.500142
C	-1.942881	0.330673	0.241735
C	-3.177811	0.071663	0.827877
H	-3.687790	0.838010	1.402872
C	-3.747938	-1.197011	0.661123
H	-4.712917	-1.404915	1.113638
C	-3.105791	-2.180647	-0.081493
H	-3.566424	-3.154039	-0.213929

C	-1.863721	-1.916549	-0.678413
H	-1.370931	-2.667508	-1.286754
C	-1.930784	2.767115	0.605823
H	-2.904870	2.850923	0.107739
H	-1.309551	3.618190	0.312179
H	-2.084687	2.819613	1.688654
C	2.487834	2.563758	0.050376
H	2.637659	2.756193	1.118349
H	2.030635	3.449175	-0.399560
H	3.462938	2.392593	-0.418539
N	-0.018291	-0.234210	-0.988622
N	1.617315	1.423303	-0.165052
N	-1.251924	1.537648	0.237953
P	0.074752	1.540625	-0.902878

2

M06-2X/6-31G(d) SCF energy in solution: -1458.95521682 a.u.

M06-2X/6-31G(d) enthalpy in solution: -1458.475184 a.u.

M06-2X/6-31G(d) free energy in solution: -1458.555590 a.u.

M06-2X/6-311+G(d,p) SCF energy in solution: -1459.30178963 a.u.

M06-2X/6-311+G(d,p) enthalpy in solution: -1458.821757 a.u.

M06-2X/6-311+G(d,p) free energy in solution: -1458.902163 a.u.

Cartesian coordinates

ATOM	X	Y	Z
P	0.431072	1.253936	-2.175525
N	1.246294	0.895365	-0.690657
O	-0.641914	-1.272575	-0.662523
O	-1.466573	-1.487883	1.475619
N	0.955110	-1.208493	1.236146
C	-0.384145	2.394889	-0.004545
C	0.796107	1.710942	0.357918
N	-0.833453	2.019097	-1.273197
C	2.104056	-1.122277	0.423470
C	2.227329	-0.108413	-0.553200
C	3.338707	-0.097008	-1.401118
H	3.416098	0.691837	-2.145636
C	-2.006695	-1.732215	-0.794507
C	1.357974	1.885326	1.612732
H	2.264183	1.351224	1.884166
C	3.156473	-2.031107	0.577721
H	3.077349	-2.798091	1.342602
C	-0.991167	3.270146	0.887110

H	-1.894853	3.802965	0.607714
B	-0.353057	-1.296855	0.684828
C	0.737500	2.762413	2.514355
H	1.173725	2.908492	3.497574
C	4.350746	-1.043102	-1.270206
H	5.207519	-1.015715	-1.935792
C	-1.880856	2.772479	-1.932149
H	-1.587939	3.816560	-2.113246
H	-2.789118	2.764134	-1.319367
H	-2.113521	2.302058	-2.891405
C	-2.656531	-1.015073	-1.965051
H	-2.562711	0.068560	-1.860719
H	-3.717632	-1.278224	-2.033252
H	-2.167993	-1.317045	-2.897236
C	1.154488	-1.448661	2.662188
H	1.387222	-2.497622	2.882237
H	0.241157	-1.181915	3.196966
H	1.973935	-0.826804	3.036375
C	-2.615625	-1.381933	0.603163
C	-1.950005	-3.235749	-1.053238
H	-1.340075	-3.418450	-1.942967
H	-2.948372	-3.648677	-1.226199
H	-1.494290	-3.764352	-0.209067
C	-3.112977	0.058468	0.682014
H	-3.315335	0.307603	1.728281
H	-4.035748	0.191652	0.108280
H	-2.356613	0.749527	0.296997
C	4.268815	-1.997068	-0.258531

H	5.061390	-2.727237	-0.127393
C	-3.689509	-2.342177	1.085758
H	-3.301885	-3.358346	1.189089
H	-4.531104	-2.355083	0.385080
H	-4.062664	-2.014679	2.061239
C	-0.417161	3.448235	2.154742
H	-0.883632	4.132351	2.856781
H	1.167222	2.443704	-2.544918

3

M06-2X/6-31G(d) SCF energy in solution: -1918.58514824 a.u.

M06-2X/6-31G(d) enthalpy in solution: -1918.110184 a.u.

M06-2X/6-31G(d) free energy in solution: -1918.194369 a.u.

M06-2X/6-311+G(d,p) SCF energy in solution: -1918.96574089 a.u.

M06-2X/6-311+G(d,p) enthalpy in solution: -1918.490777 a.u.

M06-2X/6-311+G(d,p) free energy in solution: -1918.574962 a.u.

Cartesian coordinates

ATOM	X	Y	Z
P	-1.045129	-1.101154	-1.443476
Cl	-3.152236	-2.080502	-1.778363
N	-1.350532	-0.110569	-0.109950
O	1.246703	0.691435	-0.973302
O	2.562353	1.044684	0.881121
N	0.299269	1.978310	0.930288
C	-0.586549	-2.021231	0.921598
C	-1.139032	-0.747784	1.121169
N	-0.378084	-2.267843	-0.436590
C	-0.929442	2.273685	0.307021
C	-1.741097	1.253767	-0.231210
C	-2.938341	1.565246	-0.875701
H	-3.540756	0.754440	-1.275196
C	2.599940	0.336736	-1.353083
C	-1.398309	-0.272228	2.400547
H	-1.827336	0.714595	2.545931
C	-1.400715	3.589985	0.242877
H	-0.796798	4.383960	0.671131

C	-0.285922	-2.848410	1.999353
H	0.137672	-3.834916	1.840878
B	1.332804	1.238055	0.291860
C	-1.091779	-1.099139	3.482103
H	-1.285553	-0.746779	4.490031
C	-3.366968	2.885499	-0.968980
H	-4.301062	3.117582	-1.469691
C	0.082079	-3.563944	-0.910719
H	-0.645897	-4.345500	-0.668591
H	1.043226	-3.805632	-0.446529
H	0.216105	-3.523718	-1.993939
C	2.559457	-0.893714	-2.241891
H	2.031596	-1.719450	-1.758315
H	3.576708	-1.220525	-2.482455
H	2.046025	-0.654267	-3.178736
C	0.590587	2.665211	2.187853
H	0.927707	3.696590	2.032148
H	1.378801	2.121843	2.711402
H	-0.302851	2.683054	2.819337
C	3.283451	0.117911	0.034832
C	3.176589	1.527007	-2.114352
H	2.529503	1.748190	-2.968273
H	4.181809	1.310159	-2.487559
H	3.223880	2.416888	-1.477584
C	3.043046	-1.282179	0.592157
H	3.347875	-1.303902	1.642774
H	3.621949	-2.033203	0.045685
H	1.981597	-1.546540	0.534758

C	-2.602220	3.894920	-0.389089
H	-2.933472	4.927582	-0.436714
C	4.762107	0.461902	0.073731
H	4.934987	1.513432	-0.166613
H	5.314998	-0.158891	-0.639066
H	5.158132	0.267278	1.075249
C	-0.544759	-2.369548	3.284452
H	-0.317686	-2.997112	4.140156

4d

M06-2X/6-31G(d) SCF energy in solution: -442.26201163 a.u.

M06-2X/6-31G(d) enthalpy in solution: -442.064739 a.u.

M06-2X/6-31G(d) free energy in solution: -442.107777 a.u.

M06-2X/6-311+G(d,p) SCF energy in solution: -442.38259002 a.u.

M06-2X/6-311+G(d,p) enthalpy in solution: -442.185317 a.u.

M06-2X/6-311+G(d,p) free energy in solution: -442.228355 a.u.

Imaginary frequency: -43.3018 cm-1

Cartesian coordinates

ATOM	X	Y	Z
C	-0.302982	0.434472	-0.003702
C	-0.550424	-0.962844	-0.000509
C	0.733713	-1.600589	0.001213
C	1.683743	-0.609508	-0.001314
N	1.059062	0.624368	-0.007192
H	0.938975	-2.662635	0.003218
C	1.695111	1.926381	0.005209
H	2.774784	1.804047	-0.079712
H	1.342579	2.526131	-0.839058
H	1.470578	2.456579	0.936088
C	3.171192	-0.733478	0.000776
H	3.614496	-0.254300	0.880672
H	3.446297	-1.790193	0.011472
H	3.615979	-0.272497	-0.888121
C	-1.881568	-1.412825	0.001230
C	-2.908361	-0.480565	0.001576
C	-2.637397	0.902925	0.000361

C	-1.334484	1.380119	-0.001736
H	-2.101447	-2.477038	0.002893
H	-3.940409	-0.818350	0.003468
H	-3.463511	1.607669	0.001824
H	-1.123003	2.445483	-0.001017

5d

M06-2X/6-31G(d) SCF energy in solution: -852.79523914 a.u.

M06-2X/6-31G(d) enthalpy in solution: -852.412623 a.u.

M06-2X/6-31G(d) free energy in solution: -852.480487 a.u.

M06-2X/6-311+G(d,p) SCF energy in solution: -853.03488760 a.u.

M06-2X/6-311+G(d,p) enthalpy in solution: -852.652271 a.u.

M06-2X/6-311+G(d,p) free energy in solution: -852.720135 a.u.

Cartesian coordinates

ATOM	X	Y	Z
O	1.763817	1.161368	-0.142019
O	1.397519	-1.082966	0.123862
C	3.051213	0.574249	0.162450
C	4.115236	1.242325	-0.691279
H	3.857048	1.208514	-1.752161
H	5.082443	0.749096	-0.547744
H	4.219313	2.290788	-0.394925
C	2.808533	-0.934463	-0.157352
C	3.319180	0.829769	1.643396
H	3.274944	1.906921	1.830174
H	4.308906	0.467495	1.937124
H	2.566373	0.340238	2.270188
C	3.007088	-1.265182	-1.634428
H	2.628500	-2.273898	-1.824762
H	4.065358	-1.234011	-1.910428
H	2.457411	-0.565091	-2.272396
C	3.593301	-1.902205	0.711691
H	3.342418	-1.785291	1.768449

H	4.669055	-1.741021	0.584433
H	3.364411	-2.930462	0.414755
B	0.823127	0.162418	-0.012306
C	-2.965222	-0.024569	0.004618
C	-1.704939	-0.665485	0.023438
C	-0.696999	0.370934	-0.017041
C	-1.390403	1.571993	-0.055999
N	-2.743157	1.336942	-0.045287
C	-3.808041	2.320939	-0.072369
H	-4.460267	2.143740	-0.932402
H	-4.402947	2.261744	0.843926
H	-3.381285	3.319923	-0.153520
C	-0.855574	2.965485	-0.102117
H	-1.207892	3.553038	0.752908
H	0.234208	2.937602	-0.081942
H	-1.175527	3.483063	-1.013459
C	-1.664554	-2.068244	0.073152
C	-2.858961	-2.775064	0.102232
C	-4.101278	-2.111642	0.083600
C	-4.173846	-0.726000	0.034073
H	-0.709198	-2.584303	0.088301
H	-2.839216	-3.860267	0.140828
H	-5.017705	-2.693487	0.108128
H	-5.128973	-0.209264	0.019917

6

M06-2X/6-31G(d) SCF energy in solution: -2330.27552196 a.u.

M06-2X/6-31G(d) enthalpy in solution: -2329.595858 a.u.

M06-2X/6-31G(d) free energy in solution: -2329.699499 a.u.

M06-2X/6-311+G(d,p) SCF energy in solution: -2330.77969784 a.u.

M06-2X/6-311+G(d,p) enthalpy in solution: -2330.100034 a.u.

M06-2X/6-311+G(d,p) free energy in solution: -2330.203675 a.u.

Cartesian coordinates

ATOM	X	Y	Z
P	-0.555493	-0.719483	-0.517412
N	0.034086	0.666605	0.248581
O	2.249582	-0.311500	-1.266868
O	4.157935	-0.071779	-0.003725
N	2.628492	1.839117	-0.081285
C	0.277703	-0.893546	1.924232
C	0.409165	0.460122	1.584850
N	-0.142132	-1.646699	0.822379
C	1.475994	2.481438	-0.576953
C	0.197657	1.903337	-0.434999
C	-0.929022	2.539747	-0.957232
H	-1.898262	2.073052	-0.806665
C	3.121594	-1.413729	-1.621287
C	0.855512	1.389077	2.513623
H	0.950580	2.436568	2.243478
C	1.565403	3.737336	-1.188777
H	2.540597	4.204263	-1.287460
C	0.577462	-1.343483	3.204499

H	0.466415	-2.390981	3.465935
B	2.985256	0.508901	-0.433708
C	1.166485	0.937726	3.798771
H	1.517373	1.649172	4.539437
C	-0.813490	3.766235	-1.603788
H	-1.696429	4.253803	-2.003828
C	-0.353318	-3.081540	0.926237
H	-1.192542	-3.304476	1.594850
H	0.552939	-3.559861	1.312498
H	-0.567973	-3.489040	-0.063903
C	2.284594	-2.665994	-1.812462
H	1.679909	-2.880953	-0.927983
H	2.931377	-3.526903	-2.012550
H	1.614497	-2.534511	-2.668246
C	3.589850	2.664389	0.648225
H	4.225298	3.255774	-0.021317
H	4.231685	2.013999	1.244707
H	3.061662	3.348154	1.319690
C	4.099864	-1.461402	-0.404061
C	3.818041	-1.032836	-2.924537
H	3.058725	-0.820177	-3.682863
H	4.452447	-1.846829	-3.287515
H	4.436512	-0.138285	-2.794955
C	3.529722	-2.246139	0.773985
H	4.145292	-2.056905	1.658577
H	3.528989	-3.322074	0.573323
H	2.504343	-1.928383	0.992521
C	0.437534	4.371238	-1.701103

H	0.541879	5.337176	-2.185467
C	5.502564	-1.938465	-0.737159
H	5.984600	-1.283379	-1.466496
H	5.471504	-2.955562	-1.141810
H	6.111987	-1.950812	0.171803
C	1.027382	-0.408173	4.140589
H	1.269846	-0.737758	5.145728
Cl	-2.938643	1.309844	1.884077
O	-2.401132	-0.475504	-0.218520
O	-4.460450	-0.799695	0.801618
C	-3.346225	-0.980317	-1.242285
C	-3.069057	-0.311755	-2.577653
H	-2.983437	0.772683	-2.476781
H	-3.895790	-0.533987	-3.261171
H	-2.149969	-0.698033	-3.027431
C	-4.694237	-0.569190	-0.583701
C	-3.175413	-2.491375	-1.335127
H	-2.158708	-2.736802	-1.659687
H	-3.864228	-2.903831	-2.077815
H	-3.367122	-2.970109	-0.369788
C	-5.032764	0.903501	-0.829201
H	-5.848398	1.186188	-0.156782
H	-5.362327	1.064652	-1.860483
H	-4.180892	1.557080	-0.622769
C	-5.862490	-1.441069	-1.023252
H	-5.722733	-2.477187	-0.706637
H	-5.984165	-1.412963	-2.111204
H	-6.782790	-1.063911	-0.566572

B	-3.127169	-0.484584	1.149799
H	-2.597476	-1.221684	1.940652

7

M06-2X/6-31G(d) SCF energy in solution: -1413.40396324 a.u.

M06-2X/6-31G(d) enthalpy in solution: -1413.161186 a.u.

M06-2X/6-31G(d) free energy in solution: -1413.220133 a.u.

M06-2X/6-311+G(d,p) SCF energy in solution: -1413.63654636 a.u.

M06-2X/6-311+G(d,p) enthalpy in solution: -1413.393769 a.u.

M06-2X/6-311+G(d,p) free energy in solution: -1413.452716 a.u.

Cartesian coordinates

ATOM	X	Y	Z
P	0.378572	-1.596521	-0.604209
Cl	0.260361	-2.290382	1.604175
N	-0.255516	-0.032200	-0.449548
C	2.004933	0.375267	-0.283930
C	0.722143	0.933259	-0.161513
N	1.933079	-0.966250	-0.664005
C	-1.659845	0.179952	-0.300694
C	-2.511801	-0.168279	-1.348676
H	-2.091570	-0.555224	-2.272610
C	0.557367	2.278560	0.148930
H	-0.432839	2.714367	0.226308
C	-3.547704	0.864424	1.027171
H	-3.948659	1.263331	1.953569
C	3.144952	1.142912	-0.069785
H	4.134008	0.705285	-0.156262
C	1.701834	3.048998	0.358317
H	1.592877	4.099615	0.606969
C	-3.887039	-0.005275	-1.199017

H	-4.550331	-0.277552	-2.013819
C	3.126162	-1.797884	-0.748943
H	3.589621	-1.909887	0.236757
H	3.841282	-1.341928	-1.438235
H	2.850090	-2.782802	-1.130547
C	-4.406008	0.517173	-0.015944
H	-5.477261	0.652434	0.094754
C	2.977097	2.489132	0.255576
H	3.852446	3.106285	0.429928
C	-2.173430	0.686727	0.895066
H	-1.499300	0.929733	1.710872

8

M06-2X/6-31G(d) SCF energy in solution: -2330.28329486 a.u.

M06-2X/6-31G(d) enthalpy in solution: -2329.603065 a.u.

M06-2X/6-31G(d) free energy in solution: -2329.713641 a.u.

M06-2X/6-311+G(d,p) SCF energy in solution: -2330.78823726 a.u.

M06-2X/6-311+G(d,p) enthalpy in solution: -2330.108007 a.u.

M06-2X/6-311+G(d,p) free energy in solution: -2330.218583 a.u.

Cartesian coordinates

ATOM	X	Y	Z
P	-0.551294	0.532641	0.433507
N	0.940078	1.320855	0.321739
O	1.399132	-1.079075	-1.132132
O	3.516748	-1.845491	-0.660307
N	3.281359	0.545340	-1.126751
C	1.144356	0.266615	2.355981
C	1.739393	1.168864	1.462274
N	-0.047150	-0.241945	1.835245
C	2.518788	1.658790	-1.530726
C	1.349853	2.037781	-0.838081
C	0.587693	3.122082	-1.274261
H	-0.304762	3.389404	-0.715403
C	1.339677	-2.525280	-1.203494
C	2.956652	1.771278	1.757147
H	3.409663	2.469000	1.059275
C	2.927296	2.449628	-2.611047
H	3.836536	2.183589	-3.141364
C	1.753679	-0.047703	3.567373

H	1.289142	-0.742764	4.259206
B	2.730346	-0.755837	-0.961771
C	3.570021	1.452404	2.968801
H	4.520808	1.912072	3.218549
C	0.986841	3.867396	-2.379034
H	0.388765	4.710813	-2.707728
C	-0.899555	-1.124124	2.619535
H	-1.285703	-0.602579	3.502502
H	-0.322554	-1.997712	2.939000
H	-1.735542	-1.457287	2.001504
C	0.027448	-2.993861	-0.600792
H	-0.101086	-2.620324	0.418048
H	-0.014004	-4.088206	-0.583154
H	-0.804390	-2.628188	-1.210937
C	4.734451	0.707067	-1.099262
H	5.183493	0.619292	-2.095446
H	5.163180	-0.066602	-0.460211
H	4.992875	1.686872	-0.686350
C	2.614393	-2.947649	-0.404390
C	1.414359	-2.903310	-2.680083
H	0.597802	-2.407777	-3.213650
H	1.314248	-3.983813	-2.818887
H	2.362325	-2.579772	-3.123086
C	2.372914	-2.993308	1.101537
H	3.336352	-3.071201	1.614378
H	1.758996	-3.855440	1.380225
H	1.872436	-2.080905	1.443134
C	2.171450	3.538506	-3.033794

H	2.506173	4.122861	-3.885207
C	3.262560	-4.235725	-0.881078
H	3.589403	-4.157572	-1.920561
H	2.558534	-5.069956	-0.793850
H	4.135769	-4.458972	-0.260249
C	2.976333	0.556386	3.862095
H	3.469428	0.325777	4.800883
Cl	-1.836631	2.246164	1.436919
O	-3.578048	-0.901606	0.668466
O	-5.231957	0.668642	0.862691
C	-4.403940	-1.036453	-0.521368
C	-3.518040	-1.286869	-1.728268
H	-2.726692	-0.537791	-1.810486
H	-4.118221	-1.267912	-2.644346
H	-3.056049	-2.275282	-1.646388
C	-5.170101	0.323376	-0.546374
C	-5.321851	-2.231617	-0.279834
H	-4.708018	-3.112021	-0.068068
H	-5.937212	-2.441218	-1.159769
H	-5.981963	-2.057402	0.576229
C	-4.387019	1.428576	-1.247703
H	-4.869085	2.389245	-1.042257
H	-4.371325	1.271781	-2.330491
H	-3.357108	1.477524	-0.879575
C	-6.584207	0.243643	-1.095071
H	-7.209343	-0.425331	-0.499255
H	-6.569440	-0.115014	-2.129643
H	-7.037198	1.239795	-1.085130

B	-4.209721	0.008654	1.478136
H	-3.934887	0.156077	2.624654

9

M06-2X/6-31G(d) SCF energy in solution: -2330.27652706 a.u.

M06-2X/6-31G(d) enthalpy in solution: -2329.597617 a.u.

M06-2X/6-31G(d) free energy in solution: -2329.707220 a.u.

M06-2X/6-311+G(d,p) SCF energy in solution: -2330.78256073 a.u.

M06-2X/6-311+G(d,p) enthalpy in solution: -2330.103651 a.u.

M06-2X/6-311+G(d,p) free energy in solution: -2330.213254 a.u.

Cartesian coordinates

ATOM	X	Y	Z
P	-0.668303	0.241344	-0.189127
Cl	-3.543460	0.187059	2.647833
N	0.722352	1.133867	0.353860
O	2.200941	-0.597853	-1.386136
O	4.096316	-1.226794	-0.241556
N	3.523314	1.155508	-0.228627
C	0.429653	-0.398502	2.066418
C	1.093320	0.780110	1.656391
N	-0.373734	-0.914877	1.051079
C	2.692849	2.218057	-0.638597
C	1.303435	2.186981	-0.381943
C	0.488480	3.212945	-0.869706
H	-0.578567	3.174412	-0.662678
C	2.481233	-1.962261	-1.772665
C	1.991494	1.411807	2.502654
H	2.498138	2.318108	2.183745
C	3.227089	3.334026	-1.291061
H	4.297599	3.373614	-1.469369

C	0.669552	-0.944604	3.320183
H	0.159473	-1.850964	3.631739
B	3.257391	-0.191631	-0.599258
C	2.230468	0.858685	3.770049
H	2.929614	1.350747	4.439077
C	1.029704	4.290243	-1.565102
H	0.381065	5.079320	-1.931788
C	-1.032475	-2.196234	1.192413
H	-1.845685	-2.165048	1.928387
H	-0.300192	-2.951042	1.505285
H	-1.456673	-2.492306	0.231461
C	1.169098	-2.718895	-1.882505
H	0.586297	-2.627117	-0.962765
H	1.353225	-3.779810	-2.083257
H	0.576724	-2.310617	-2.708042
C	4.796914	1.495574	0.397930
H	5.552964	1.807964	-0.332605
H	5.175005	0.619693	0.928124
H	4.657161	2.307827	1.118377
C	3.418899	-2.447676	-0.619929
C	3.187077	-1.913367	-3.125270
H	2.550432	-1.380102	-3.837546
H	3.374817	-2.919076	-3.513255
H	4.142300	-1.382432	-3.052280
C	2.639186	-2.923998	0.602298
H	3.328581	-3.031488	1.445387
H	2.159920	-3.890822	0.418282
H	1.868652	-2.194414	0.873081

C	2.407926	4.361057	-1.752208
H	2.848559	5.206546	-2.271341
C	4.452878	-3.480406	-1.032812
H	5.124443	-3.089626	-1.800817
H	3.959728	-4.378713	-1.419087
H	5.052806	-3.767431	-0.163493
C	1.580359	-0.301898	4.172826
H	1.771236	-0.718577	5.156916
O	-5.052859	0.808368	0.444811
O	-3.514829	-0.861652	0.107243
C	-5.067219	0.527152	-0.989573
C	-6.502035	0.574381	-1.480251
H	-7.149004	-0.080678	-0.892832
H	-6.547699	0.268102	-2.530332
H	-6.881500	1.597614	-1.404444
C	-4.402195	-0.886958	-1.055806
C	-4.230895	1.612411	-1.656884
H	-4.645435	2.590173	-1.395560
H	-4.250339	1.505277	-2.745184
H	-3.189085	1.576803	-1.319647
C	-5.389687	-2.023319	-0.816885
H	-4.831545	-2.953648	-0.677868
H	-6.059904	-2.144246	-1.672547
H	-5.993815	-1.844577	0.078760
C	-3.577293	-1.145204	-2.301623
H	-2.774980	-0.413402	-2.416257
H	-4.220684	-1.106890	-3.186878
H	-3.131565	-2.143429	-2.245220

B	-4.062792	0.043529	0.959808
H	-1.668671	1.061938	0.458331

10

M06-2X/6-31G(d) SCF energy in solution:	-1313.57065249 a.u.
M06-2X/6-31G(d) enthalpy in solution:	-1313.174605 a.u.
M06-2X/6-31G(d) free energy in solution:	-1313.245422 a.u.
M06-2X/6-311+G(d,p) SCF energy in solution:	-1313.85019584 a.u.
M06-2X/6-311+G(d,p) enthalpy in solution:	-1313.454148 a.u.
M06-2X/6-311+G(d,p) free energy in solution:	-1313.524965 a.u.

Cartesian coordinates

ATOM	X	Y	Z
O	-1.678249	-1.113074	-0.137417
O	-1.261115	1.129345	0.270156
C	-2.946481	-0.475783	0.063288
C	-3.958523	-1.082368	-0.897472
H	-3.593290	-1.060109	-1.927182
H	-4.910111	-0.541437	-0.849355
H	-4.147029	-2.125871	-0.624073
C	-2.606874	1.023321	-0.210807
C	-3.395444	-0.715645	1.505971
H	-3.398189	-1.793237	1.699452
H	-4.405954	-0.331202	1.679683
H	-2.709952	-0.243003	2.215091
C	-2.616844	1.358375	-1.703842
H	-2.142579	2.334280	-1.850092
H	-3.637176	1.409092	-2.098128
H	-2.062608	0.614259	-2.284383
C	-3.479102	2.018093	0.541711
H	-3.371407	1.901742	1.622759

H	-4.534045	1.887238	0.276042
H	-3.186360	3.039658	0.277028
B	-0.677932	-0.163889	0.206162
C	2.770186	0.065871	-0.186817
C	1.653375	0.782766	-0.626771
C	0.607600	-0.203218	-0.966306
C	1.278057	-1.480896	-0.836022
N	2.487517	-1.310431	-0.333657
H	0.026452	-0.070346	-1.879409
C	3.411513	-2.345568	0.104708
H	4.392594	-2.153684	-0.333962
H	3.484240	-2.319825	1.195118
H	3.050919	-3.320157	-0.218954
C	0.692330	-2.816100	-1.117262
H	0.601527	-3.389675	-0.187902
H	-0.298903	-2.693050	-1.551106
H	1.332227	-3.379713	-1.803656
C	1.682248	2.174117	-0.587241
C	2.837429	2.799798	-0.121432
C	3.940868	2.056821	0.320010
C	3.925245	0.663936	0.300636
H	0.820450	2.752379	-0.903827
H	2.882851	3.884064	-0.091615
H	4.822341	2.573516	0.685866
H	4.771301	0.080755	0.649456
Cl	0.172421	-0.596148	1.944231

11

M06-2X/6-31G(d) SCF energy in solution: -831.66462955 a.u.

M06-2X/6-31G(d) enthalpy in solution: -831.368570 a.u.

M06-2X/6-31G(d) free energy in solution: -831.423069 a.u.

M06-2X/6-311+G(d,p) SCF energy in solution: -831.81996344 a.u.

M06-2X/6-311+G(d,p) enthalpy in solution: -831.523904 a.u.

M06-2X/6-311+G(d,p) free energy in solution: -831.578403 a.u.

Cartesian coordinates

ATOM	X	Y	Z
N	0.330369	-0.114987	0.258870
C	1.327796	0.395512	-0.764112
H	0.921529	0.069078	-1.723289
C	2.708958	-0.204589	-0.555439
H	2.706029	-1.294728	-0.638149
H	3.373403	0.188917	-1.329883
H	3.128941	0.073102	0.416388
C	1.336973	1.919262	-0.761483
H	1.918244	2.257273	-1.624003
H	0.320731	2.313684	-0.857612
H	1.803040	2.334728	0.136724
C	0.111948	-1.618810	0.228239
H	1.006080	-2.056007	0.682357
C	-1.121012	-1.952401	1.062849
H	-1.262117	-3.036393	1.055959
H	-1.030920	-1.635668	2.104828
H	-2.009027	-1.483960	0.625063
C	-0.066176	-2.128030	-1.196632

H	-0.868116	-1.578930	-1.701619
H	0.846885	-2.061526	-1.792276
H	-0.352215	-3.182151	-1.145298
C	0.623835	0.373054	1.658175
H	0.768139	-0.503364	2.291077
H	1.569744	0.912514	1.635941
C	-0.486296	1.265060	2.184400
H	-1.445295	0.739403	2.208335
H	-0.237338	1.580037	3.201492
H	-0.600347	2.158212	1.563168
Cl	-2.288282	0.965697	-1.058941
H	-0.585327	0.299482	-0.055279

12

M06-2X/6-31G(d) SCF energy in solution:	-1918.58064577 a.u.
M06-2X/6-31G(d) enthalpy in solution:	-1918.105682 a.u.
M06-2X/6-31G(d) free energy in solution:	-1918.190618 a.u.
M06-2X/6-311+G(d,p) SCF energy in solution:	-1918.96078521 a.u.
M06-2X/6-311+G(d,p) enthalpy in solution:	-1918.485821 a.u.
M06-2X/6-311+G(d,p) free energy in solution:	-1918.570757 a.u.

Cartesian coordinates

ATOM	X	Y	Z
P	3.307589	0.250514	0.979476
Cl	4.915837	0.659151	-0.657095
N	1.865072	0.419970	0.114988
O	-2.766339	0.783160	-0.479920
O	-2.774588	-0.740326	1.248788
N	-0.819645	0.725626	1.044484
C	2.240567	-1.842553	-0.079022
C	1.439420	-0.768800	-0.497342
N	3.201965	-1.420449	0.841025
C	-0.153998	1.779677	0.385142
C	1.181955	1.661895	-0.041327
C	1.841730	2.741971	-0.629526
H	2.871592	2.615910	-0.950402
C	-3.882706	-0.107712	-0.717060
C	0.389218	-0.960335	-1.387946
H	-0.235014	-0.127592	-1.699763
C	-0.815817	2.989710	0.145514
H	-1.848903	3.082739	0.464858

C	2.022897	-3.129266	-0.561846
H	2.651049	-3.955350	-0.245020
B	-2.093727	0.271309	0.608202
C	0.172504	-2.252147	-1.871223
H	-0.633114	-2.423563	-2.577989
C	1.174837	3.945390	-0.830570
H	1.692993	4.780576	-1.289996
C	4.248183	-2.317668	1.312304
H	4.889964	-2.631713	0.482477
H	3.794892	-3.197174	1.776210
H	4.853565	-1.800605	2.059593
C	-5.052321	0.698372	-1.253884
H	-5.286072	1.544729	-0.603795
H	-5.941356	0.065156	-1.342694
H	-4.806999	1.084394	-2.248189
C	-0.180942	0.089976	2.197025
H	0.389319	-0.806901	1.925750
H	-0.954822	-0.202007	2.910440
H	0.492663	0.798960	2.686642
C	-4.113006	-0.733733	0.696346
C	-3.425540	-1.140101	-1.743392
H	-3.067607	-0.618946	-2.636979
H	-4.246136	-1.803401	-2.033360
H	-2.607094	-1.748917	-1.345473
C	-4.967920	0.151900	1.598898
H	-4.918241	-0.232403	2.622014
H	-6.014250	0.152875	1.279327
H	-4.601433	1.184082	1.600116

C	-0.163865	4.058748	-0.458775
H	-0.698872	4.989177	-0.621442
C	-4.651327	-2.153683	0.681582
H	-3.964736	-2.836844	0.176461
H	-5.620432	-2.186799	0.172630
H	-4.792715	-2.503959	1.708760
C	0.977941	-3.319576	-1.466032
H	0.792146	-4.313332	-1.860343

TS1

M06-2X/6-31G(d) SCF energy in solution: -2330.20382382 a.u.

M06-2X/6-31G(d) enthalpy in solution: -2329.527168 a.u.

M06-2X/6-31G(d) free energy in solution: -2329.633426 a.u.

M06-2X/6-311+G(d,p) SCF energy in solution: -2330.70823326 a.u.

M06-2X/6-311+G(d,p) enthalpy in solution: -2330.031577 a.u.

M06-2X/6-311+G(d,p) free energy in solution: -2330.137835 a.u.

Imaginary frequency: -627.2287 cm-1

Cartesian coordinates

ATOM	X	Y	Z
P	2.031014	1.367682	0.295963
Cl	2.983923	2.258859	1.952460
N	0.435640	1.053532	0.902955
O	-3.715620	-1.098805	0.711538
O	-3.432141	-0.755446	-1.549751
N	-1.414063	-0.861012	-0.162484
C	0.070457	2.863298	-0.455160
C	-0.501092	1.994449	0.490994
N	1.408663	2.538071	-0.738863
C	-0.810145	-1.050249	1.098519
C	0.126863	-0.132314	1.617393
C	0.772961	-0.402246	2.827165
H	1.489125	0.316540	3.211822
C	-5.029072	-0.838458	0.162535
C	-1.826640	2.140375	0.881726
H	-2.266947	1.452246	1.598154
C	-1.122982	-2.182909	1.858167

H	-1.853686	-2.880709	1.461142
C	-0.667969	3.905259	-1.002155
H	-0.227378	4.580826	-1.727274
B	-2.823886	-0.903507	-0.321499
C	-2.569013	3.186705	0.326791
H	-3.603710	3.319323	0.627186
C	0.474623	-1.552519	3.550195
H	0.983143	-1.746726	4.488942
C	2.174358	3.445346	-1.586133
H	2.190752	4.449344	-1.149206
H	1.710801	3.487533	-2.575193
H	3.192772	3.074833	-1.691523
C	-6.039887	-1.740708	0.848336
H	-5.736294	-2.789095	0.801342
H	-7.023221	-1.636254	0.377761
H	-6.132840	-1.455472	1.900940
C	-0.556411	-0.634547	-1.325522
H	-0.445656	0.429682	-1.569690
H	-0.995275	-1.135280	-2.192198
H	0.437147	-1.055158	-1.147428
C	-4.816456	-1.131623	-1.357433
C	-5.348773	0.628869	0.433093
H	-5.261002	0.819776	1.507337
H	-6.365951	0.880226	0.117403
H	-4.646501	1.282942	-0.093741
C	-4.929668	-2.617291	-1.689561
H	-4.569917	-2.780360	-2.709829
H	-5.966539	-2.960977	-1.627924

H	-4.319918	-3.220810	-1.008443
C	-0.493315	-2.433484	3.072571
H	-0.745331	-3.327869	3.633844
C	-5.688955	-0.310942	-2.290945
H	-5.501093	0.759097	-2.176883
H	-6.747776	-0.506885	-2.091405
H	-5.479770	-0.588883	-3.328678
C	-1.997175	4.059432	-0.597754
H	-2.586969	4.869328	-1.014474
O	2.854644	-0.650649	-1.489160
O	4.106148	-0.604268	0.472003
C	4.018016	-1.496345	-1.701911
C	3.593485	-2.699960	-2.522681
H	2.733711	-3.203421	-2.074189
H	4.419457	-3.414527	-2.602950
H	3.318696	-2.379271	-3.532283
C	4.448290	-1.823495	-0.237479
C	5.057649	-0.667324	-2.449427
H	4.613756	-0.296314	-3.378067
H	5.936639	-1.268169	-2.700820
H	5.382452	0.192597	-1.853390
C	3.615505	-2.943130	0.381989
H	3.819206	-2.980818	1.456274
H	3.864793	-3.915343	-0.053621
H	2.543886	-2.759165	0.242561
C	5.931717	-2.094924	-0.061839
H	6.532236	-1.230065	-0.352915
H	6.234100	-2.955725	-0.667681

H	6.142111	-2.324208	0.987344
B	3.103170	-0.023411	-0.282514
H	3.508514	1.547576	-0.500586

TS2

M06-2X/6-31G(d) SCF energy in solution: -2330.26533312 a.u.

M06-2X/6-31G(d) enthalpy in solution: -2329.586764 a.u.

M06-2X/6-31G(d) free energy in solution: -2329.692745 a.u.

M06-2X/6-311+G(d,p) SCF energy in solution: -2330.77102885 a.u.

M06-2X/6-311+G(d,p) enthalpy in solution: -2330.092460 a.u.

M06-2X/6-311+G(d,p) free energy in solution: -2330.198441 a.u.

Imaginary frequency: -42.4682 cm-1

Cartesian coordinates

ATOM	X	Y	Z
P	-0.484723	-0.534700	-0.366522
N	0.286763	0.785686	0.304282
O	2.248236	-0.474128	-1.303359
O	4.270972	-0.369067	-0.212469
N	2.937208	1.683944	-0.277884
C	0.376888	-0.694087	2.046114
C	0.694793	0.602926	1.624240
N	-0.258079	-1.401427	1.032145
C	1.817322	2.422258	-0.706543
C	0.505435	1.987433	-0.437116
C	-0.601987	2.710805	-0.876310
H	-1.593190	2.343595	-0.621621
C	2.967868	-1.686400	-1.647934
C	1.321200	1.508563	2.479279
H	1.552469	2.515512	2.146398
C	1.970112	3.643513	-1.373535
H	2.971913	4.009114	-1.575823

C	0.693847	-1.132155	3.333435
H	0.448670	-2.138301	3.656677
B	3.132466	0.307789	-0.584106
C	1.635964	1.071790	3.759183
H	2.129708	1.751817	4.445482
C	-0.425928	3.901700	-1.572741
H	-1.289108	4.465740	-1.909500
C	-0.715093	-2.774830	1.222787
H	-1.424131	-2.812183	2.053444
H	0.142642	-3.418916	1.436574
H	-1.210519	-3.116616	0.313269
C	1.990534	-2.847913	-1.688969
H	1.445408	-2.951381	-0.746897
H	2.524543	-3.783533	-1.885776
H	1.266233	-2.691191	-2.494938
C	4.042404	2.439107	0.313833
H	4.678487	2.909358	-0.444748
H	4.657099	1.757800	0.904146
H	3.649255	3.219309	0.972289
C	4.033571	-1.766672	-0.508744
C	3.588320	-1.462438	-3.023539
H	2.794698	-1.213758	-3.734171
H	4.101448	-2.361705	-3.376539
H	4.305842	-0.635338	-3.003088
C	3.485307	-2.409169	0.762140
H	4.192086	-2.240010	1.579929
H	3.348927	-3.487584	0.636418
H	2.523469	-1.964470	1.041692

C	0.865213	4.371569	-1.804024
H	1.017896	5.308200	-2.331058
C	5.345162	-2.415161	-0.914142
H	5.830393	-1.863251	-1.722385
H	5.173069	-3.445319	-1.243045
H	6.023612	-2.438922	-0.055775
C	1.327795	-0.232208	4.180067
H	1.586971	-0.542914	5.186806
Cl	-2.732013	1.127480	1.895480
O	-2.866484	-0.910935	-0.066632
O	-4.943452	-0.402732	0.809985
C	-3.823994	-1.039196	-1.131719
C	-3.223782	-0.535236	-2.436670
H	-2.805756	0.469641	-2.325827
H	-3.991238	-0.510008	-3.218299
H	-2.424572	-1.205035	-2.771441
C	-5.019032	-0.189934	-0.599423
C	-4.169023	-2.523236	-1.255378
H	-3.248264	-3.087302	-1.437721
H	-4.855991	-2.710862	-2.086753
H	-4.624941	-2.894088	-0.331622
C	-4.850330	1.301406	-0.899148
H	-5.582440	1.861964	-0.308805
H	-5.021793	1.518052	-1.958879
H	-3.852086	1.649120	-0.619625
C	-6.381419	-0.658687	-1.092198
H	-6.596763	-1.675477	-0.754685
H	-6.430418	-0.631844	-2.186465

H	-7.160974	0.003282	-0.700812
B	-3.588348	-0.614743	1.164325
H	-3.400490	-1.359400	2.099052

TS3

M06-2X/6-31G(d) SCF energy in solution: -2330.26032053 a.u.

M06-2X/6-31G(d) enthalpy in solution: -2329.582776 a.u.

M06-2X/6-31G(d) free energy in solution: -2329.689347 a.u.

M06-2X/6-311+G(d,p) SCF energy in solution: -2330.76536713 a.u.

M06-2X/6-311+G(d,p) enthalpy in solution: -2330.087823 a.u.

M06-2X/6-311+G(d,p) free energy in solution: -2330.194394 a.u.

Imaginary frequency: -54.4032 cm-1

Cartesian coordinates

ATOM	X	Y	Z
P	-0.511788	-0.090663	-0.261542
Cl	-3.357631	0.217855	2.540858
N	0.555584	1.018535	0.399830
O	2.133491	-0.554595	-1.351859
O	4.155300	-0.960912	-0.331409
N	3.311310	1.337733	-0.250846
C	0.407034	-0.518202	2.093016
C	0.961664	0.706240	1.698246
N	-0.369964	-1.067767	1.073901
C	2.372038	2.320993	-0.616926
C	1.003454	2.166218	-0.322878
C	0.067407	3.119463	-0.721237
H	-0.979915	2.962146	-0.476592
C	2.559681	-1.872201	-1.786143
C	1.791252	1.430923	2.552243
H	2.213182	2.380951	2.239689
C	2.771534	3.504247	-1.248875

H	3.824422	3.654449	-1.466304
C	0.669720	-1.055714	3.353474
H	0.235698	-2.002375	3.657091
B	3.187757	-0.029031	-0.627820
C	2.051255	0.894620	3.807653
H	2.693460	1.437624	4.493216
C	0.481774	4.268221	-1.386917
H	-0.247809	5.010621	-1.692095
C	-1.007901	-2.372373	1.236911
H	-1.782056	-2.307465	2.006039
H	-0.245024	-3.102184	1.522963
H	-1.468855	-2.665437	0.295485
C	1.349547	-2.786648	-1.855866
H	0.820141	-2.829214	-0.900505
H	1.658195	-3.801859	-2.126796
H	0.656777	-2.423260	-2.621963
C	4.569934	1.802203	0.332682
H	5.269935	2.169363	-0.426451
H	5.040009	0.971390	0.861110
H	4.374764	2.608293	1.046222
C	3.606017	-2.251995	-0.690298
C	3.183702	-1.706769	-3.168554
H	2.448550	-1.248406	-3.836523
H	3.478468	-2.673245	-3.587600
H	4.065672	-1.058755	-3.128905
C	2.957018	-2.829067	0.564667
H	3.702074	-2.867490	1.364873
H	2.580926	-3.841600	0.389085

H	2.125007	-2.199201	0.899646
C	1.839542	4.464250	-1.630741
H	2.178230	5.364780	-2.133376
C	4.731000	-3.150061	-1.173071
H	5.308893	-2.673332	-1.968112
H	4.326856	-4.096100	-1.548167
H	5.405888	-3.372407	-0.340810
C	1.498098	-0.332470	4.203327
H	1.717551	-0.724440	5.190932
O	-4.378496	1.106132	0.081201
O	-3.186390	-0.881204	-0.064075
C	-4.777303	0.443185	-1.121019
C	-6.264567	0.670273	-1.346814
H	-6.845012	0.394599	-0.462954
H	-6.621370	0.082994	-2.200288
H	-6.449491	1.728131	-1.560976
C	-4.384853	-1.038167	-0.830354
C	-3.974808	1.033737	-2.283291
H	-4.126120	2.117784	-2.300163
H	-4.291167	0.625097	-3.248501
H	-2.902660	0.840346	-2.158236
C	-5.420469	-1.750862	0.040183
H	-4.988595	-2.688241	0.405766
H	-6.330112	-1.985702	-0.522473
H	-5.687947	-1.135722	0.904921
C	-4.074802	-1.868712	-2.066516
H	-3.221000	-1.459180	-2.613313
H	-4.939297	-1.903996	-2.738900

H	-3.831909	-2.895588	-1.772346
B	-3.254383	0.395124	0.586788
H	-2.148227	0.989698	0.460540

TS4

M06-2X/6-31G(d) SCF energy in solution:	-1313.56759462 a.u.
M06-2X/6-31G(d) enthalpy in solution:	-1313.172418 a.u.
M06-2X/6-31G(d) free energy in solution:	-1313.243057 a.u.
M06-2X/6-311+G(d,p) SCF energy in solution:	-1313.84655268 a.u.
M06-2X/6-311+G(d,p) enthalpy in solution:	-1313.451376 a.u.
M06-2X/6-311+G(d,p) free energy in solution:	-1313.522015 a.u.
Imaginary frequency:	-215.2575 cm-1

Cartesian coordinates

ATOM	X	Y	Z
O	-1.672957	-1.119424	0.019226
O	-1.247163	1.116279	0.399060
C	-2.955249	-0.456623	0.070927
C	-3.866419	-1.056622	-0.986684
H	-3.403045	-1.032838	-1.975828
H	-4.814439	-0.509574	-1.028199
H	-4.085086	-2.099040	-0.734418
C	-2.573186	1.041809	-0.168142
C	-3.536562	-0.699311	1.463357
H	-3.590842	-1.778068	1.638314
H	-4.543862	-0.281642	1.555270
H	-2.902537	-0.257809	2.239224
C	-2.505935	1.414238	-1.648327
H	-1.988319	2.373274	-1.749479
H	-3.512181	1.520726	-2.066153
H	-1.974239	0.667528	-2.242974
C	-3.458866	2.041085	0.561710

H	-3.404314	1.907085	1.644335
H	-4.501238	1.931975	0.243529
H	-3.134904	3.059520	0.324490
B	-0.733108	-0.174093	0.426847
C	2.740988	0.017019	-0.201608
C	1.685455	0.772928	-0.747661
C	0.675047	-0.178259	-1.161323
C	1.218379	-1.451129	-0.946331
N	2.424146	-1.330227	-0.345330
H	-0.115892	0.007338	-1.873127
C	3.219901	-2.400701	0.226372
H	3.126591	-2.397428	1.316840
H	2.877888	-3.358613	-0.163836
H	4.267563	-2.259325	-0.048010
C	0.597763	-2.773719	-1.230296
H	0.412225	-3.321309	-0.299319
H	-0.354987	-2.626530	-1.738838
H	1.252022	-3.385766	-1.859712
C	1.762555	2.169787	-0.721538
C	2.886600	2.763691	-0.161483
C	3.924297	1.988172	0.386897
C	3.866226	0.600036	0.381492
H	0.955900	2.773694	-1.126103
H	2.965692	3.846322	-0.138106
H	4.784714	2.483056	0.826544
H	4.659262	-0.001609	0.814613
Cl	0.293855	-0.576950	1.921974

TS5

M06-2X/6-31G(d) SCF energy in solution: -2772.51396110 a.u.

M06-2X/6-31G(d) enthalpy in solution: -2771.637238 a.u.

M06-2X/6-31G(d) free energy in solution: -2771.767267 a.u.

M06-2X/6-311+G(d,p) SCF energy in solution: -2773.14133584 a.u.

M06-2X/6-311+G(d,p) enthalpy in solution: -2772.264613 a.u.

M06-2X/6-311+G(d,p) free energy in solution: -2772.394642 a.u.

Imaginary frequency: -120.3143 cm-1

Cartesian coordinates

ATOM	X	Y	Z
P	0.713083	2.277865	0.104325
Cl	-0.083309	5.212180	0.451796
N	-0.923200	1.805468	0.206409
O	-4.714517	-0.972412	0.775376
O	-3.587264	-2.283171	-0.748683
N	-2.256479	-0.740633	0.613768
C	-0.744700	2.317339	-2.033776
C	-1.607572	1.881459	-1.012450
N	0.535511	2.576594	-1.546180
C	-2.185644	0.236125	1.628899
C	-1.533025	1.471117	1.447231
C	-1.434148	2.385020	2.500949
H	-0.933097	3.332350	2.310661
C	-5.717306	-1.580647	-0.073423
C	-2.951651	1.640590	-1.272502
H	-3.628066	1.340795	-0.477447
C	-2.766090	-0.021404	2.878652

H	-3.273084	-0.970547	3.021593
C	-1.203569	2.485978	-3.334740
H	-0.532012	2.821619	-4.118428
B	-3.495920	-1.319232	0.231918
C	-3.412830	1.820200	-2.581396
H	-4.461630	1.644797	-2.799595
C	-1.991429	2.095389	3.740082
H	-1.909357	2.810986	4.551815
C	1.503947	3.315759	-2.339756
H	1.049631	4.236402	-2.720474
H	1.858711	2.711146	-3.181707
H	2.357693	3.580115	-1.712011
C	-6.920008	-1.957945	0.772922
H	-6.629911	-2.574328	1.626981
H	-7.650087	-2.509589	0.171310
H	-7.403091	-1.051067	1.149781
C	-1.039214	-1.122819	-0.100758
H	-0.932170	-0.590423	-1.054134
H	-1.070976	-2.196059	-0.308951
H	-0.157712	-0.917820	0.509682
C	-4.941733	-2.790480	-0.686007
C	-6.102756	-0.545202	-1.126099
H	-6.429561	0.369742	-0.622143
H	-6.919614	-0.904614	-1.759311
H	-5.245825	-0.303578	-1.763794
C	-4.925941	-4.005668	0.237459
H	-4.203060	-4.732109	-0.145539
H	-5.908703	-4.484452	0.281693

H	-4.627544	-3.724616	1.253221
C	-2.672608	0.891449	3.921898
H	-3.120004	0.655060	4.882383
C	-5.387868	-3.189369	-2.081751
H	-5.251272	-2.372650	-2.794216
H	-6.444142	-3.478283	-2.074360
H	-4.801371	-4.047684	-2.424065
C	-2.552069	2.228631	-3.598749
H	-2.930450	2.359521	-4.607406
O	3.131200	0.567137	1.420400
O	2.179227	-1.353541	0.543490
C	2.753464	-0.251882	2.543097
C	3.852064	-0.173340	3.590881
H	4.827918	-0.410441	3.158978
H	3.650664	-0.867775	4.413631
H	3.895220	0.840287	4.001988
C	2.576221	-1.667104	1.894051
C	1.445641	0.302301	3.106076
H	1.583187	1.361873	3.345014
H	1.139342	-0.222917	4.016649
H	0.634978	0.215322	2.373875
C	3.883694	-2.453619	1.812921
H	3.730017	-3.324088	1.166487
H	4.197968	-2.807542	2.800188
H	4.687769	-1.845131	1.387133
C	1.497119	-2.524902	2.539422
H	0.516567	-2.043054	2.498266
H	1.743762	-2.726927	3.587470

H	1.429932	-3.483361	2.014361
C	4.851691	-1.517073	-1.536073
C	4.776049	-0.279719	-0.893974
C	3.418796	0.262689	-1.144681
C	2.851182	-0.673818	-2.109830
N	3.656009	-1.702867	-2.269040
H	3.311835	1.323096	-1.385143
C	3.399295	-2.911732	-3.040959
H	2.472009	-2.800287	-3.599404
H	4.229680	-3.068792	-3.731933
H	3.323153	-3.759672	-2.356345
C	1.517305	-0.570940	-2.749537
H	0.891345	-1.423101	-2.461420
H	1.031257	0.354863	-2.438597
H	1.617316	-0.577695	-3.840348
C	5.838677	0.142880	-0.100056
C	6.950441	-0.690182	0.010910
C	6.996642	-1.933837	-0.635453
C	5.938153	-2.375413	-1.425807
H	5.793024	1.090815	0.425672
H	7.794945	-0.376402	0.616462
H	7.871824	-2.564753	-0.519306
H	5.962750	-3.338413	-1.925040
B	2.558236	-0.024013	0.286506
H	1.355313	0.717543	-0.027096

TS6

M06-2X/6-31G(d) SCF energy in solution: -2360.81232225 a.u.

M06-2X/6-31G(d) enthalpy in solution: -2360.144111 a.u.

M06-2X/6-31G(d) free energy in solution: -2360.251494 a.u.

M06-2X/6-311+G(d,p) SCF energy in solution: -2361.31609167 a.u.

M06-2X/6-311+G(d,p) enthalpy in solution: -2360.647880 a.u.

M06-2X/6-311+G(d,p) free energy in solution: -2360.755263 a.u.

Imaginary frequency: -591.9821 cm-1

Cartesian coordinates

ATOM	X	Y	Z
P	-0.588435	-0.261199	-0.723847
N	0.329434	0.887970	0.187515
O	2.506177	-0.484833	-1.170473
O	4.202685	-0.849113	0.342759
N	3.165047	1.369889	0.337029
C	0.145070	-0.867422	1.691037
C	0.523130	0.478294	1.517500
N	-0.230296	-1.454156	0.482892
C	2.293797	2.312144	-0.247703
C	0.908118	2.056855	-0.359652
C	0.088292	2.981424	-1.011784
H	-0.973863	2.768589	-1.094571
C	3.152341	-1.716546	-1.567394
C	0.987668	1.229807	2.585148
H	1.275191	2.267412	2.439185
C	2.785739	3.535619	-0.715112
H	3.846389	3.744567	-0.610955

C	0.215024	-1.461181	2.946242
H	-0.084313	-2.495330	3.085021
B	3.265564	0.036677	-0.145643
C	1.070402	0.625608	3.847181
H	1.431396	1.205373	4.690852
C	0.604424	4.169466	-1.520117
H	-0.052599	4.872065	-2.022798
C	2.092936	-2.705660	-2.020517
H	1.328666	-2.841910	-1.251840
H	2.548468	-3.675670	-2.247273
H	1.608069	-2.333702	-2.928953
C	4.146429	1.865910	1.297366
H	4.999107	2.354845	0.810894
H	4.522993	1.025981	1.883929
H	3.676670	2.584449	1.976388
C	3.915654	-2.129669	-0.265861
C	4.093469	-1.376323	-2.720448
H	3.514405	-0.909744	-3.522918
H	4.577500	-2.273361	-3.118166
H	4.869611	-0.672073	-2.401995
C	3.036133	-2.906577	0.709400
H	3.549079	-2.974113	1.673777
H	2.841503	-3.921075	0.347165
H	2.079604	-2.395384	0.856958
C	1.955491	4.457321	-1.346709
H	2.370865	5.390002	-1.715473
C	5.223179	-2.865526	-0.504548
H	5.930443	-2.253867	-1.069463

H	5.041613	-3.794561	-1.055251
H	5.680225	-3.122086	0.456335
C	0.685171	-0.699621	4.025114
H	0.745153	-1.154485	5.008916
C	-4.467175	-0.699313	0.203671
C	-3.372927	-0.862569	-0.663710
C	-2.366804	0.133067	-0.285044
C	-2.916225	0.797977	0.837631
N	-4.153592	0.318340	1.097792
H	-2.745138	1.184310	-1.364961
C	-5.087865	0.802673	2.102017
H	-4.570456	1.460151	2.798453
H	-5.906422	1.346395	1.622654
H	-5.491941	-0.049737	2.652420
C	-2.378636	1.959636	1.603758
H	-2.216738	1.696350	2.655030
H	-1.434064	2.292760	1.176900
H	-3.089508	2.792841	1.570257
C	-3.455077	-1.817610	-1.686659
C	-4.610617	-2.580684	-1.793474
C	-5.688671	-2.402211	-0.907915
C	-5.637471	-1.452872	0.103903
H	-2.634906	-1.957459	-2.385718
H	-4.687783	-3.326374	-2.578668
H	-6.579265	-3.012106	-1.021666
H	-6.471158	-1.300641	0.782202
C	-0.822703	-2.776738	0.468353
H	-1.779125	-2.808444	1.009221

H	-0.994867	-3.085791	-0.565857
H	-0.134746	-3.494233	0.930630
Cl	-3.190952	2.053307	-2.466451

TS7

M06-2X/6-31G(d) SCF energy in solution: -2271.34383732 a.u.

M06-2X/6-31G(d) enthalpy in solution: -2270.394301 a.u.

M06-2X/6-31G(d) free energy in solution: -2270.520792 a.u.

M06-2X/6-311+G(d,p) SCF energy in solution: -2271.91180555 a.u.

M06-2X/6-311+G(d,p) enthalpy in solution: -2270.962269 a.u.

M06-2X/6-311+G(d,p) free energy in solution: -2271.088760 a.u.

Imaginary frequency: -1394.5606 cm-1

Cartesian coordinates

ATOM	X	Y	Z
P	0.004039	-0.269901	-0.697162
N	0.839333	0.581747	0.549859
O	3.093116	0.561632	-1.260549
O	5.016279	0.131544	-0.069483
N	3.460971	1.778681	0.866625
C	1.220713	-1.617282	1.153962
C	1.322217	-0.271497	1.553890
N	0.741007	-1.725505	-0.148613
C	2.303234	2.569324	0.731091
C	1.036376	1.980559	0.523799
C	-0.065980	2.795635	0.265653
H	-1.017860	2.307274	0.084049
C	3.971553	-0.170992	-2.146303
C	1.816647	0.062058	2.806630
H	1.885289	1.103002	3.109278
C	2.379233	3.965544	0.799629
H	3.345119	4.424950	0.988211

C	1.608900	-2.642444	2.011848
H	1.528547	-3.679993	1.702790
B	3.830452	0.831199	-0.127429
C	2.211453	-0.968874	3.666175
H	2.598180	-0.721183	4.649591
C	0.035081	4.181952	0.278577
H	-0.841024	4.792419	0.081350
C	3.142132	-1.149468	-2.957576
H	2.542970	-1.788827	-2.305583
H	3.789013	-1.779563	-3.577386
H	2.465405	-0.597444	-3.617932
C	4.413865	2.138736	1.912327
H	5.043543	2.990860	1.629606
H	5.062881	1.283304	2.108660
H	3.880996	2.392908	2.833883
C	4.972421	-0.835042	-1.145965
C	4.643479	0.849996	-3.060598
H	3.869746	1.417702	-3.585962
H	5.279703	0.360290	-3.803884
H	5.255532	1.552447	-2.484703
C	4.433548	-2.136475	-0.560152
H	5.059991	-2.432957	0.286742
H	4.444370	-2.942100	-1.301081
H	3.407856	-2.000701	-0.201847
C	1.262763	4.767598	0.579450
H	1.361506	5.847679	0.621641
C	6.376751	-1.032733	-1.689063
H	6.837073	-0.080763	-1.963505

H	6.355466	-1.681738	-2.570898
H	7.000969	-1.511109	-0.927820
C	2.106734	-2.302955	3.274556
H	2.412963	-3.092008	3.954072
C	-2.556268	-2.716468	0.359690
C	-2.252861	-1.789421	-0.646997
C	-1.808966	-0.529930	0.001487
C	-1.910297	-0.837606	1.405480
N	-2.357818	-2.084540	1.593598
H	-2.702909	0.481675	-0.295460
C	-2.590357	-2.753597	2.865312
H	-2.593810	-2.021323	3.670622
H	-3.559254	-3.255416	2.831426
H	-1.800764	-3.490313	3.038382
C	-1.642880	0.071612	2.555746
H	-1.035171	-0.420368	3.320727
H	-1.122296	0.965862	2.211158
H	-2.591138	0.375112	3.017142
C	-2.364391	-2.184674	-1.983138
C	-2.784806	-3.482211	-2.262759
C	-3.088799	-4.385900	-1.233093
C	-2.982058	-4.016570	0.104528
H	-2.131523	-1.493070	-2.789208
H	-2.879624	-3.802756	-3.295435
H	-3.414783	-5.390480	-1.482829
H	-3.215994	-4.710344	0.905629
C	0.674013	-3.011798	-0.817677
H	-0.090456	-3.668177	-0.382000

H	0.439996	-2.857769	-1.873156
H	1.648496	-3.509186	-0.745683
N	-3.827834	1.267966	-0.644300
C	-4.421055	1.764488	0.638336
H	-4.424121	0.874157	1.278162
C	-3.326204	2.317031	-1.584645
H	-2.585217	2.883069	-1.015592
C	-4.706940	0.279050	-1.324130
H	-5.504025	0.796398	-1.869408
H	-4.086577	-0.226793	-2.065160
C	-3.544979	2.807461	1.320750
H	-3.539120	3.755334	0.772320
H	-3.957166	3.004045	2.315670
H	-2.516454	2.464844	1.447088
C	-5.864482	2.286361	0.548920
H	-6.476971	1.751945	-0.180647
H	-6.336064	2.166590	1.529600
H	-5.893810	3.349772	0.300010
C	-2.601274	1.672411	-2.765743
H	-3.302159	1.264338	-3.501182
H	-1.998968	2.435574	-3.267111
H	-1.929216	0.871138	-2.441608
C	-4.382775	3.305853	-2.079803
H	-4.726922	3.972043	-1.286472
H	-3.934855	3.927489	-2.861939
H	-5.249664	2.799504	-2.515827
C	-5.318744	-0.764040	-0.393392
H	-4.591569	-1.162009	0.318130

H	-6.163939	-0.371546	0.177518
H	-5.681787	-1.599274	-1.000298

TS8

M06-2X/6-31G(d) SCF energy in solution: -1825.08208274 a.u.

M06-2X/6-31G(d) enthalpy in solution: -1824.635613 a.u.

M06-2X/6-31G(d) free energy in solution: -1824.716017 a.u.

M06-2X/6-311+G(d,p) SCF energy in solution: -1825.44048348 a.u.

M06-2X/6-311+G(d,p) enthalpy in solution: -1824.994014 a.u.

M06-2X/6-311+G(d,p) free energy in solution: -1825.074418 a.u.

Imaginary frequency: -21.3038 cm-1

Cartesian coordinates

ATOM	X	Y	Z
P	-0.939840	-0.548722	1.673026
Cl	-0.215195	-1.521049	-1.595203
N	-1.396272	0.689590	0.646111
C	-3.085207	-0.822028	0.302238
C	-2.563450	0.427838	-0.062709
N	-2.296374	-1.416246	1.270169
C	-0.580569	1.845688	0.418378
C	-0.165712	2.141458	-0.880972
H	-0.437898	1.475787	-1.694270
C	-3.222898	1.234280	-0.997263
H	-2.835854	2.210620	-1.265710
C	0.586677	3.765758	1.266849
H	0.878480	4.393803	2.102345
C	-4.256918	-1.321211	-0.276973
H	-4.647278	-2.293114	0.004960
C	-4.386212	0.739731	-1.562234
H	-4.916959	1.342889	-2.291314

C	0.613620	3.273508	-1.098600
H	0.938339	3.512665	-2.106375
C	-2.590628	-2.750839	1.793655
H	-2.530767	-3.477736	0.980163
H	-3.592584	-2.755041	2.227239
H	-1.859726	-3.002244	2.563153
C	0.991408	4.084984	-0.027379
H	1.602790	4.964421	-0.203013
C	-4.895314	-0.525460	-1.212180
H	-5.807274	-0.880440	-1.680328
O	1.519272	-0.822899	0.596290
O	2.595256	-2.161514	-0.948455
C	2.902888	-0.445629	0.589598
C	3.022043	1.056378	0.787407
H	2.414791	1.602299	0.062016
H	4.065565	1.376185	0.686334
H	2.681838	1.325381	1.793620
C	3.366796	-0.964520	-0.804286
C	3.595702	-1.178804	1.740268
H	3.077829	-0.939835	2.674741
H	4.643609	-0.877735	1.840631
H	3.556107	-2.263007	1.592103
C	3.000812	0.007081	-1.927765
H	3.128180	-0.500590	-2.889577
H	3.644059	0.893805	-1.916533
H	1.956359	0.320687	-1.841723
C	4.844103	-1.321099	-0.884829
H	5.095773	-2.129026	-0.193183

H	5.467362	-0.450506	-0.651769
H	5.089228	-1.652168	-1.899596
B	1.390305	-1.986229	-0.233781
H	0.894801	-2.980358	0.248834
C	-0.211905	2.646256	1.496210
H	-0.561492	2.404747	2.496093

TS9

M06-2X/6-31G(d) SCF energy in solution: -1825.07787782 a.u.

M06-2X/6-31G(d) enthalpy in solution: -1824.632560 a.u.

M06-2X/6-31G(d) free energy in solution: -1824.713497 a.u.

M06-2X/6-311+G(d,p) SCF energy in solution: -1825.43514962 a.u.

M06-2X/6-311+G(d,p) enthalpy in solution: -1824.989832 a.u.

M06-2X/6-311+G(d,p) free energy in solution: -1825.070769 a.u.

Imaginary frequency: -43.8916 cm-1

Cartesian coordinates

ATOM	X	Y	Z
P	0.395589	0.063048	-1.267543
Cl	-1.123014	-1.504271	1.958248
N	1.927465	0.195090	-0.584097
C	1.590985	-2.072362	-0.501859
C	2.446410	-1.022858	-0.137358
N	0.479540	-1.595390	-1.193489
C	2.493149	1.471524	-0.267160
C	2.691949	1.825914	1.068027
H	2.428481	1.126766	1.856326
C	3.642331	-1.269524	0.535585
H	4.306824	-0.456012	0.805782
C	3.306299	3.615237	-0.989521
H	3.542641	4.309262	-1.789588
C	1.901389	-3.395895	-0.190401
H	1.234353	-4.204988	-0.467764
C	3.948892	-2.589541	0.844908
H	4.871760	-2.808894	1.371625

C	3.217671	3.079747	1.364853
H	3.378061	3.361230	2.400610
C	-0.540227	-2.508364	-1.706111
H	-1.084696	-2.956086	-0.871032
H	-0.049747	-3.281551	-2.301953
H	-1.239454	-1.951269	-2.327207
C	3.522435	3.974933	0.339115
H	3.927372	4.953485	0.576803
C	3.089093	-3.638523	0.489539
H	3.353272	-4.658377	0.748899
O	-2.497663	0.874768	1.409082
O	-2.218135	-0.491439	-0.445674
C	-3.478730	1.251232	0.436425
C	-4.770086	1.621872	1.149814
H	-5.086815	0.829414	1.832238
H	-5.572317	1.805786	0.426540
H	-4.621647	2.536667	1.732975
C	-3.573337	-0.027036	-0.452016
C	-2.945175	2.455316	-0.342388
H	-2.693922	3.250671	0.366232
H	-3.685698	2.843084	-1.049354
H	-2.037655	2.192526	-0.898323
C	-4.451337	-1.108035	0.179286
H	-4.291176	-2.049442	-0.356305
H	-5.514194	-0.851911	0.118092
H	-4.184682	-1.260668	1.229722
C	-4.002246	0.226538	-1.888904
H	-3.285587	0.868685	-2.407909

H	-4.988804	0.702137	-1.920956
H	-4.066173	-0.723558	-2.430214
B	-1.618568	-0.037733	0.771311
H	-0.464378	0.440972	0.495742
C	2.795918	2.356659	-1.299835
H	2.639480	2.052099	-2.330741

IX. Spectral Data

^{1}H, $CDCl_3$, 400 MHz

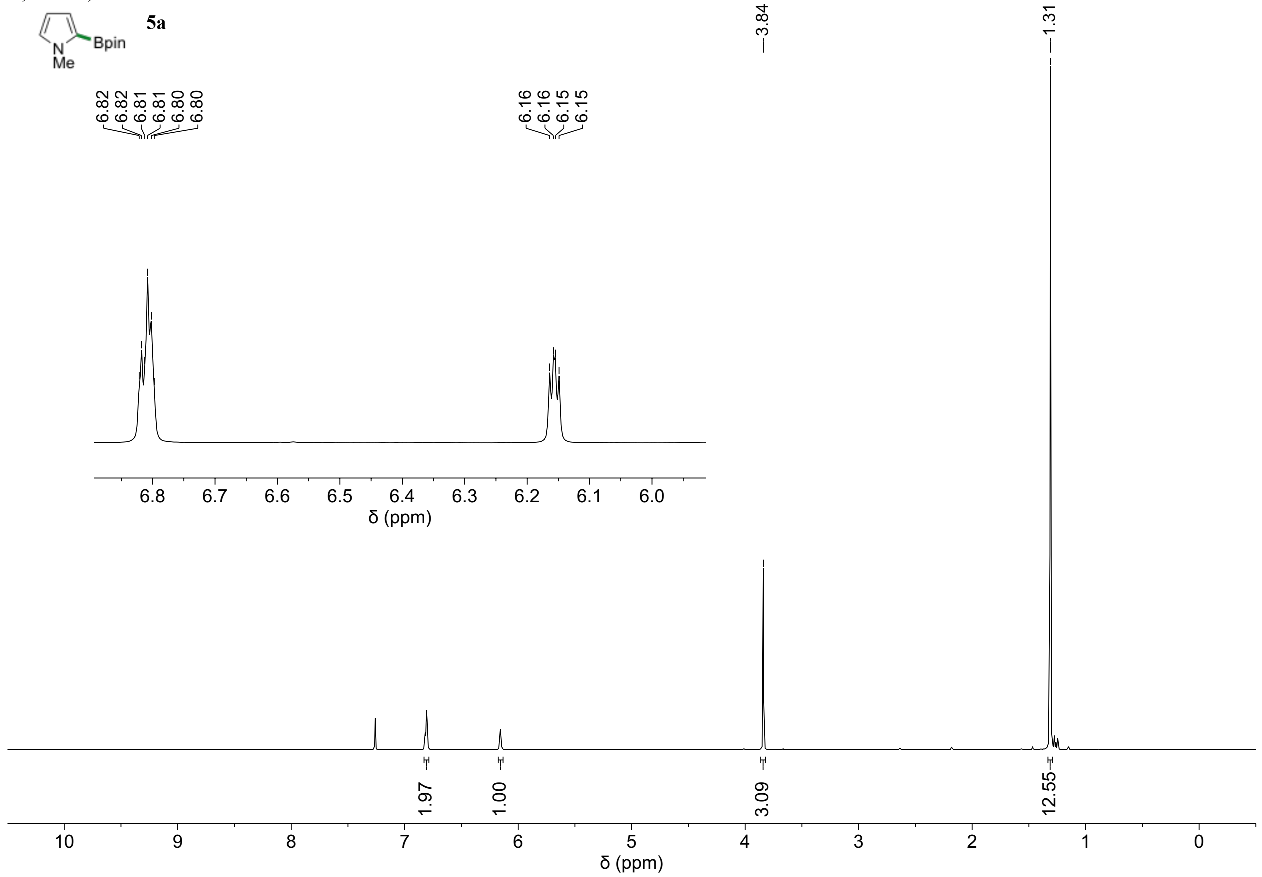

^{13}C, $CDCl_3$, 101 MHz

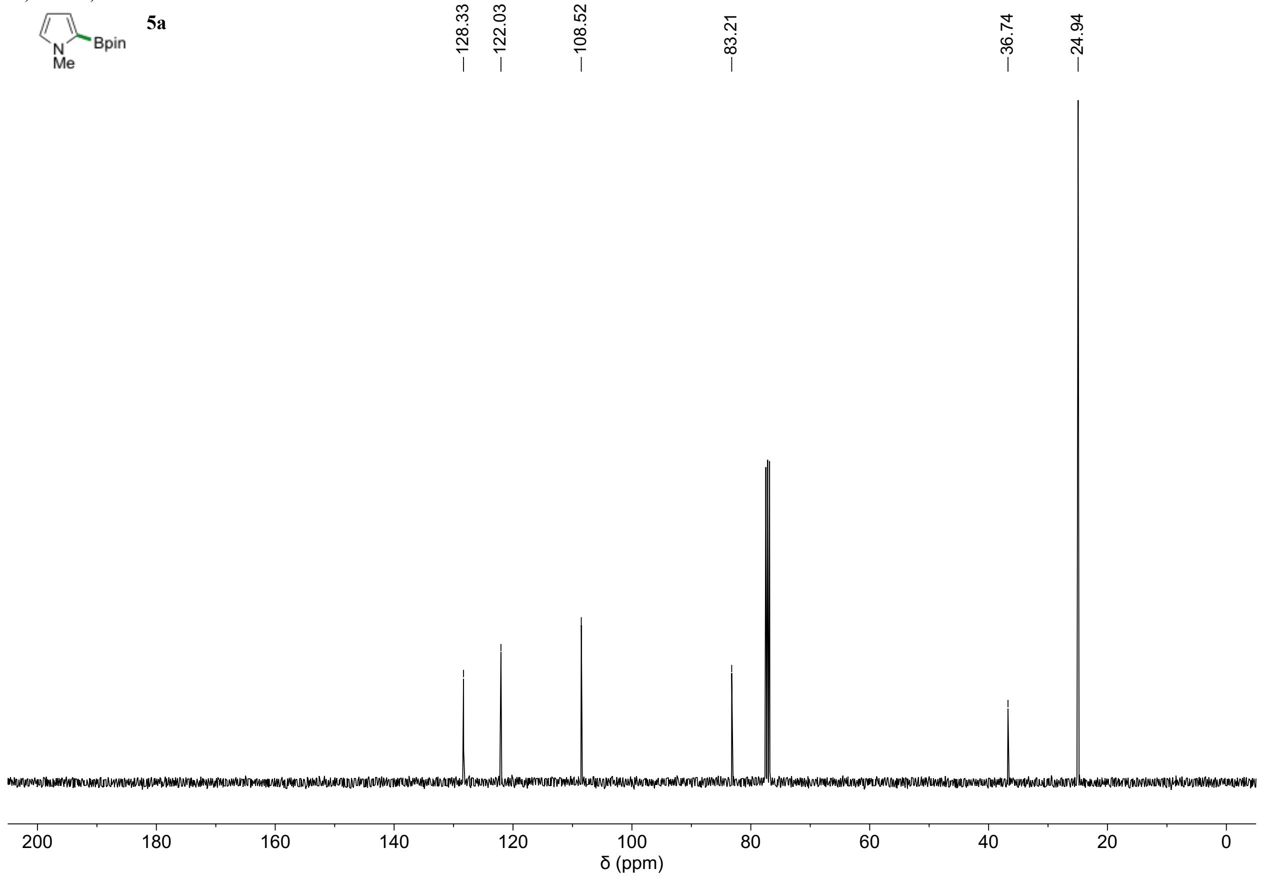

^{11}B, $CDCl_3$, 128 MHz

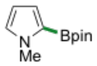

5a

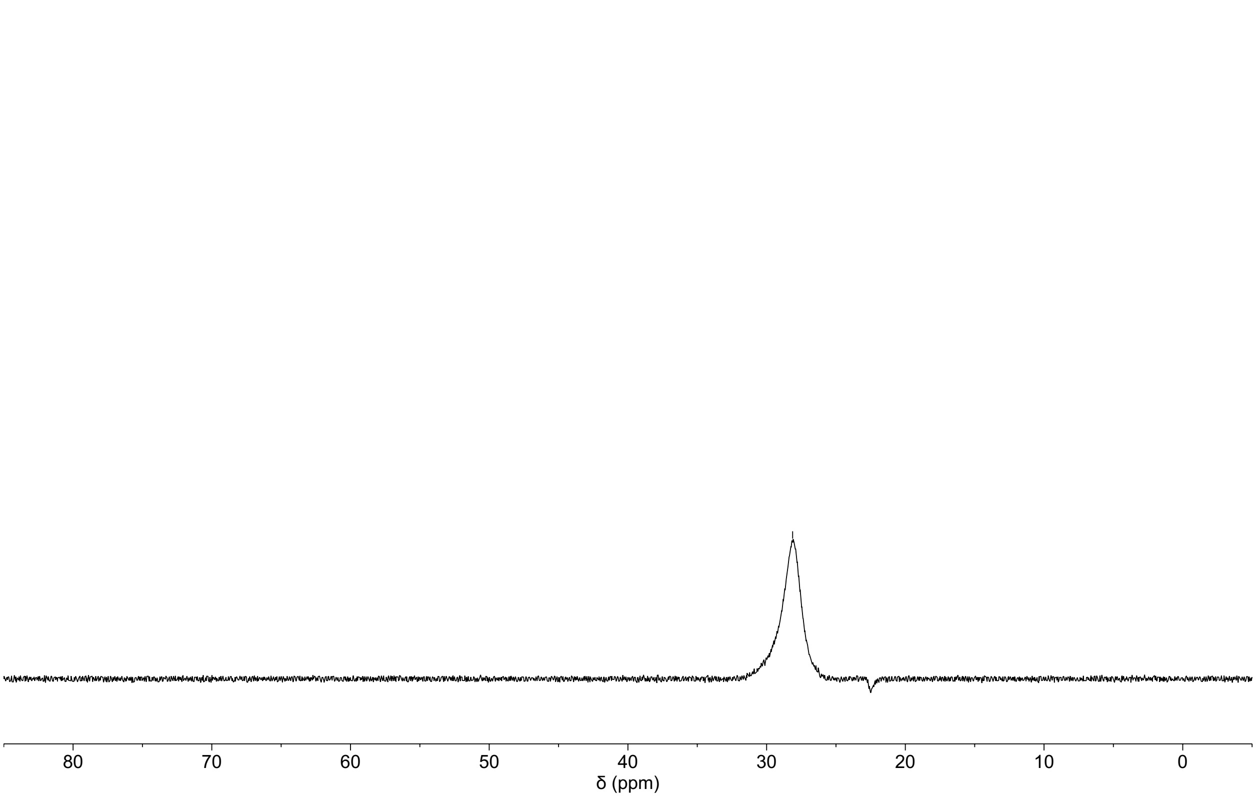

^{1}H, $CDCl_3$, 600 MHz

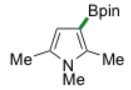

5b

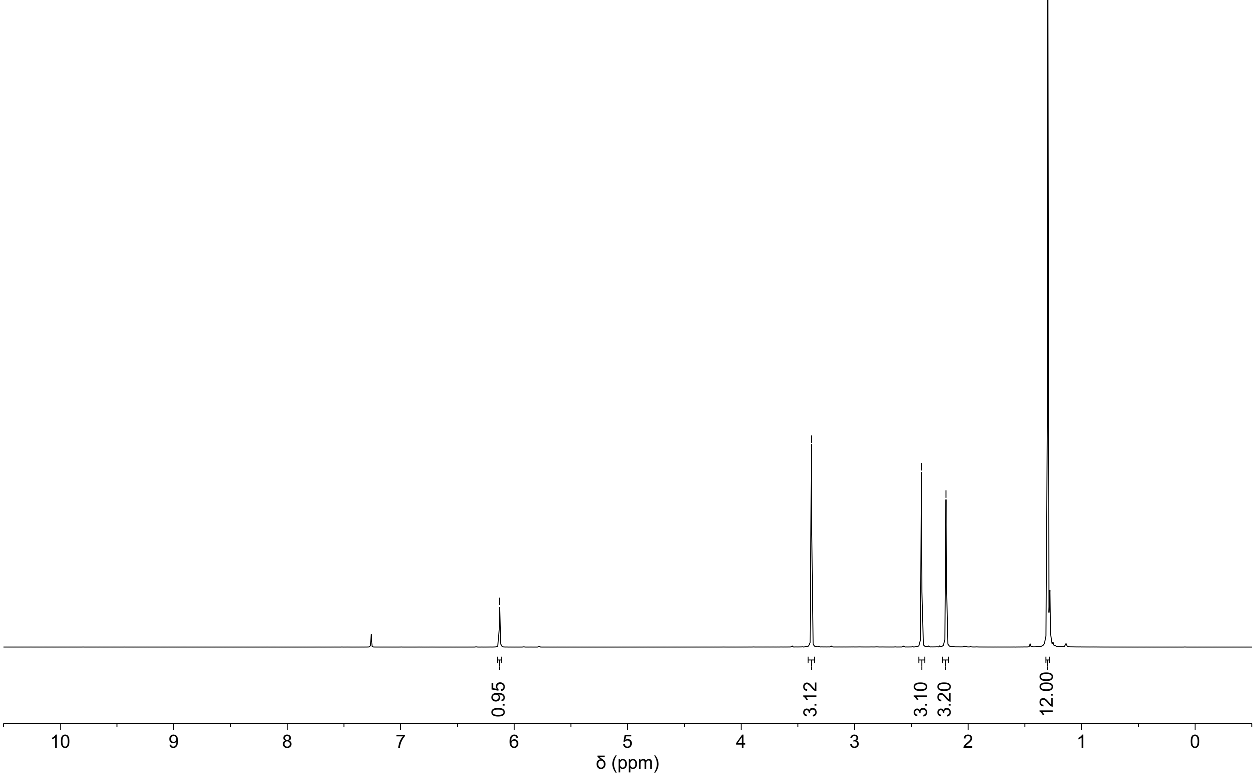

^{13}C, $CDCl_3$, 151 MHz

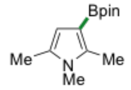

5b

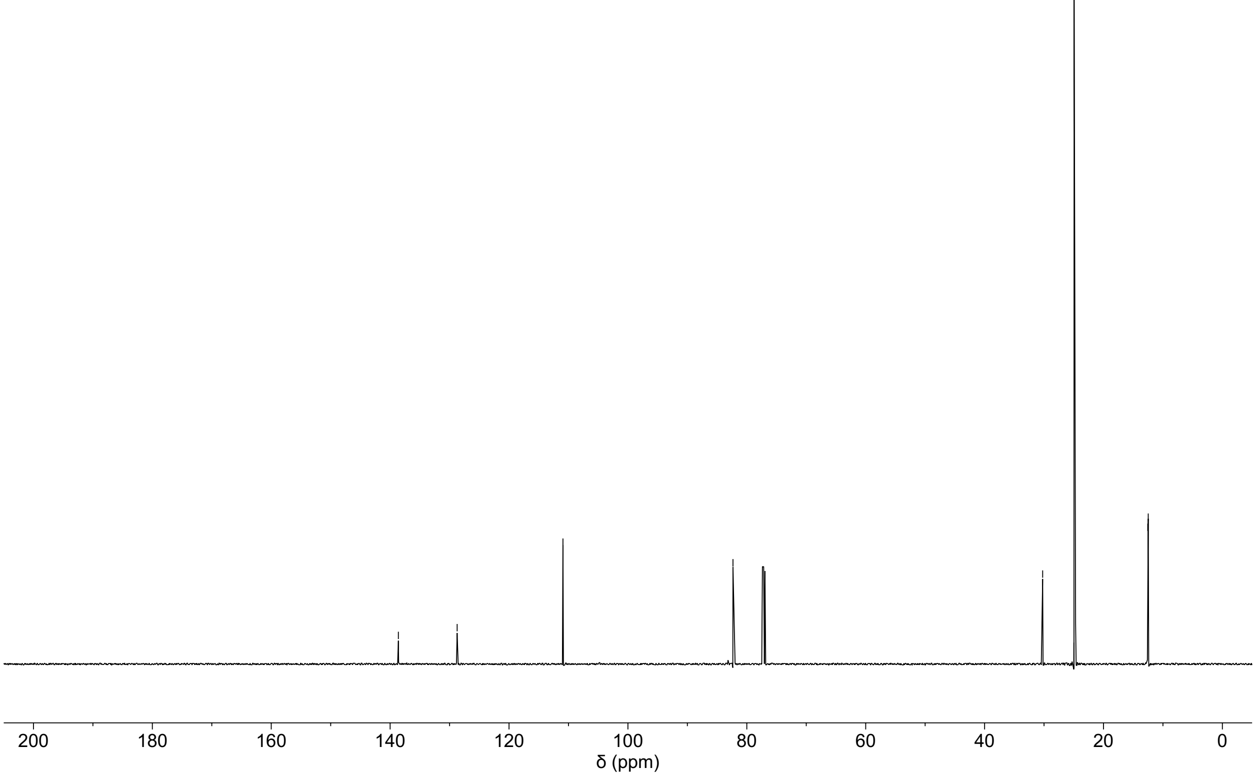

^{11}B, $CDCl_3$, 128 MHz

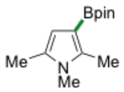

5b

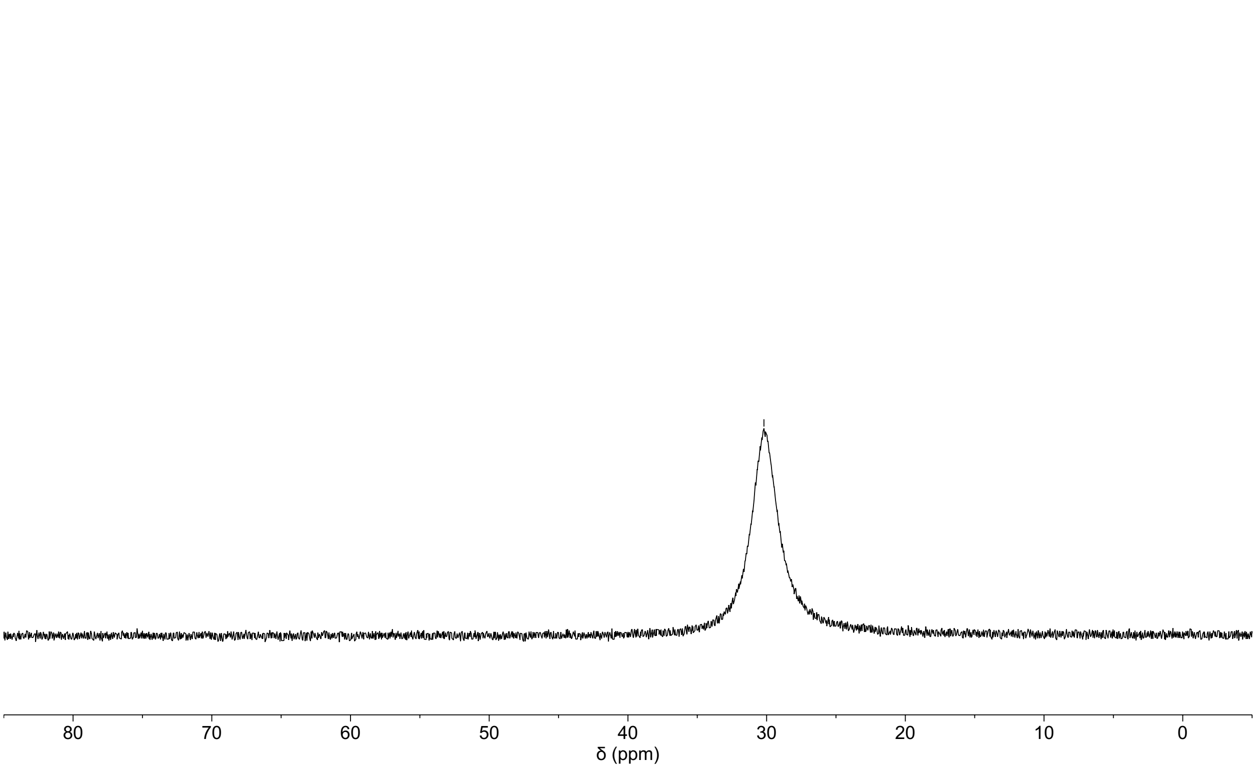

^{1}H, $CDCl_3$, 600 MHz

5c

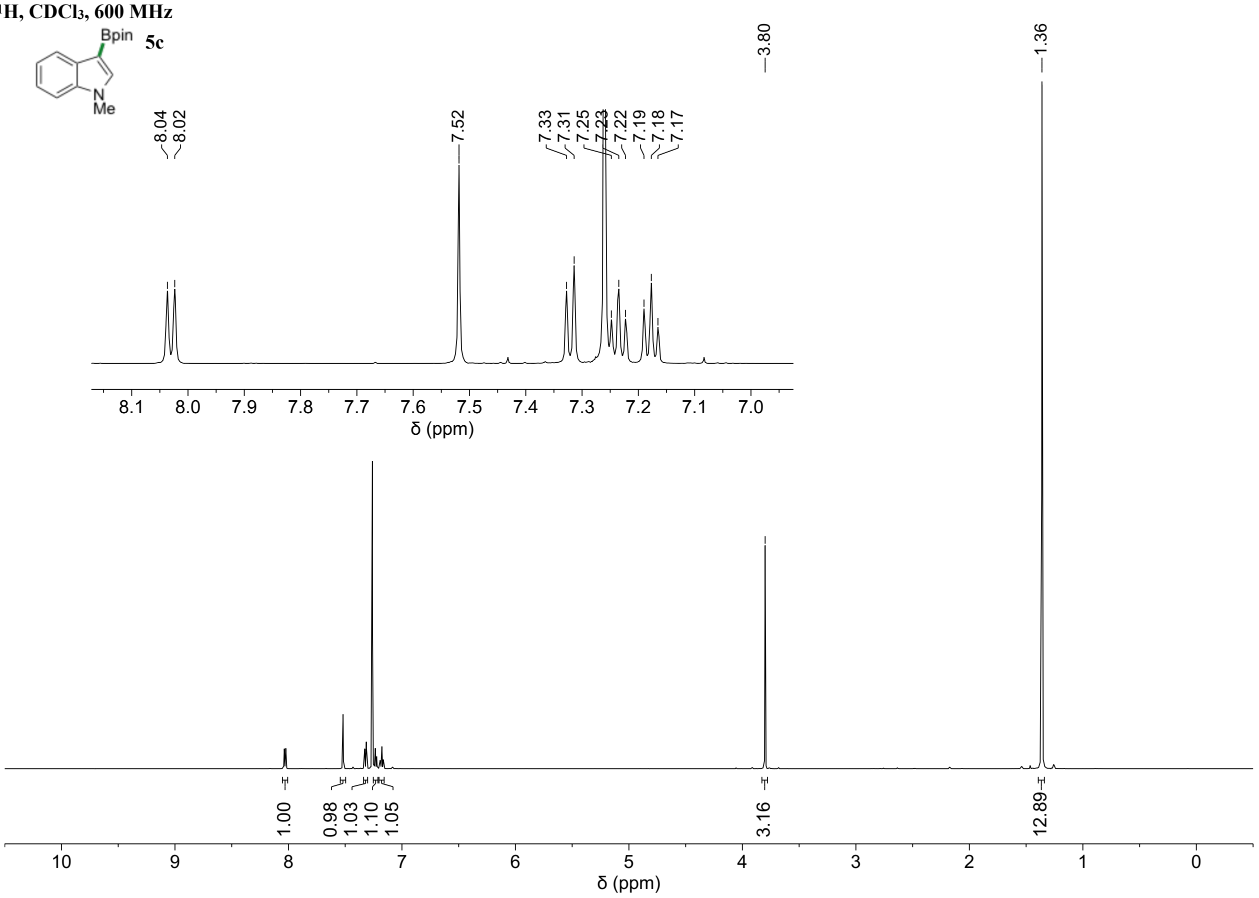

^{13}C, $CDCl_3$, 151 MHz

5c

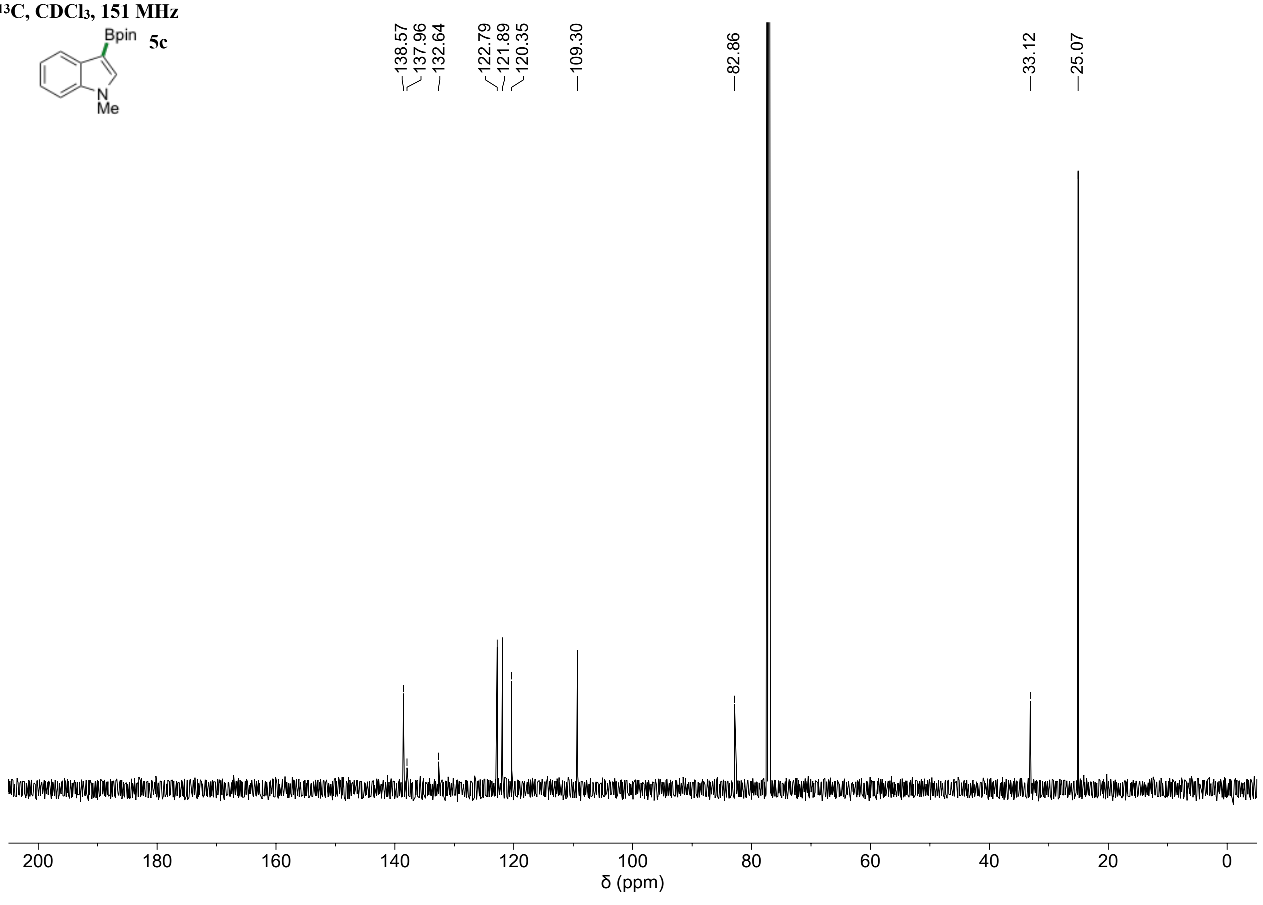

^{11}B, $CDCl_3$, 128 MHz

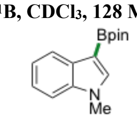

5c

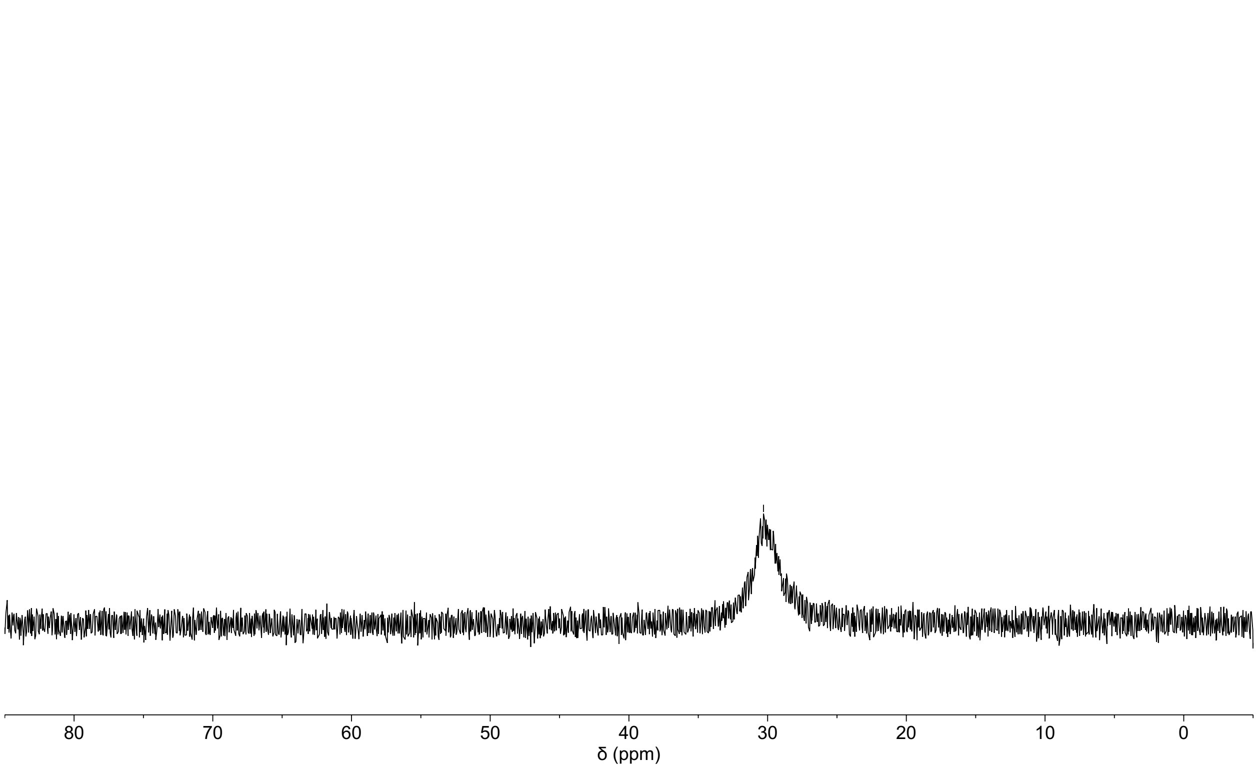

^{1}H, $CDCl_3$, 600 MHz

5d

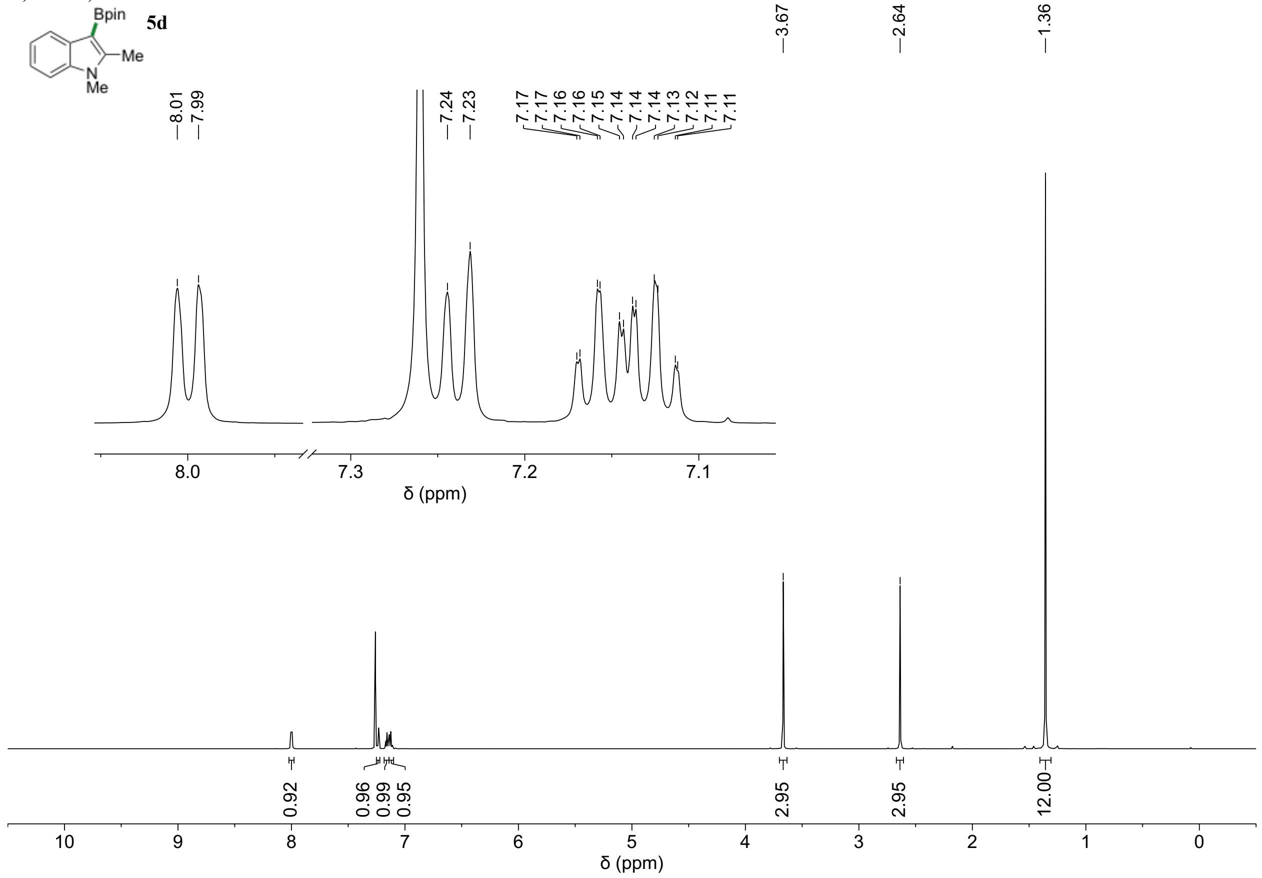

^{13}C, $CDCl_3$, 151 MHz

5d

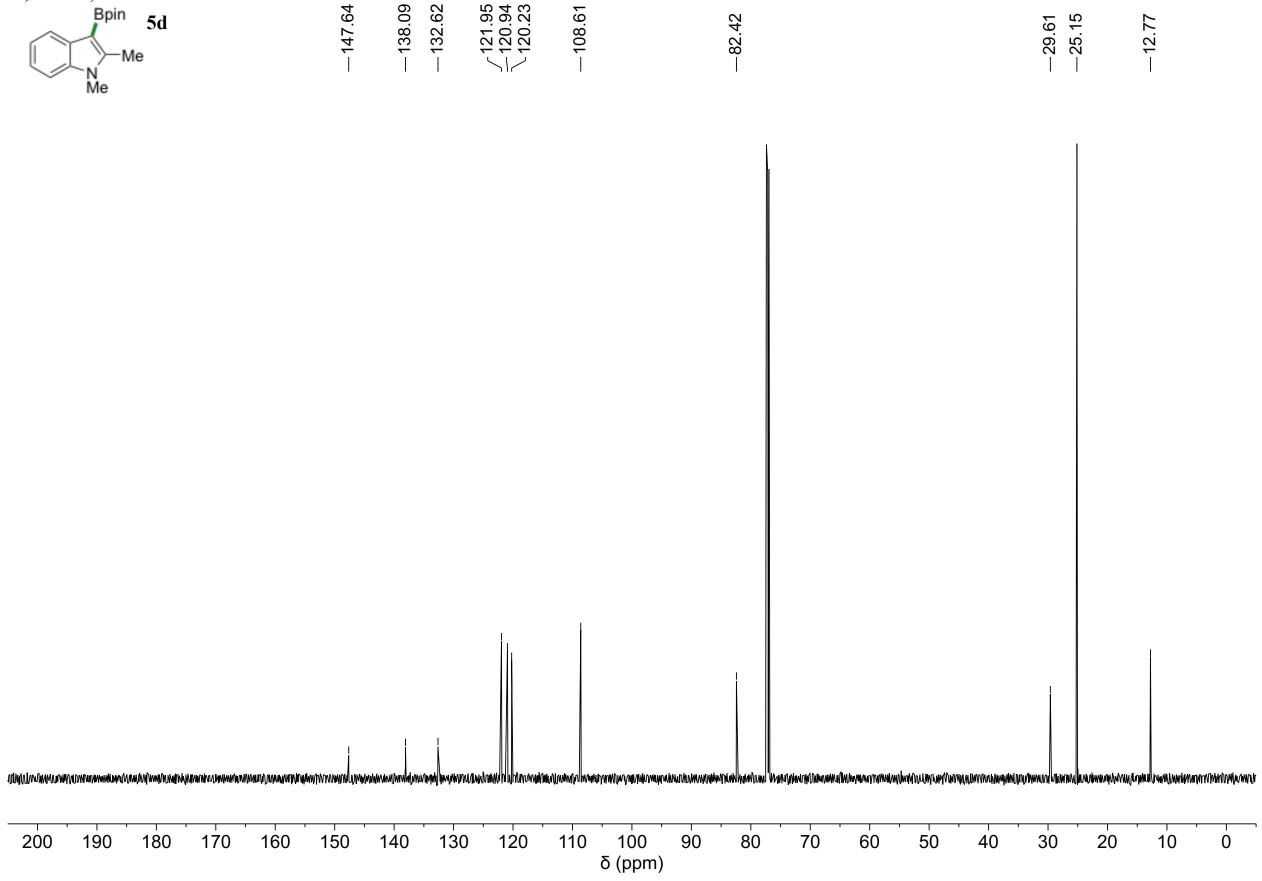

^{11}B, $CDCl_3$, 128 MHz

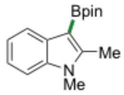

5d

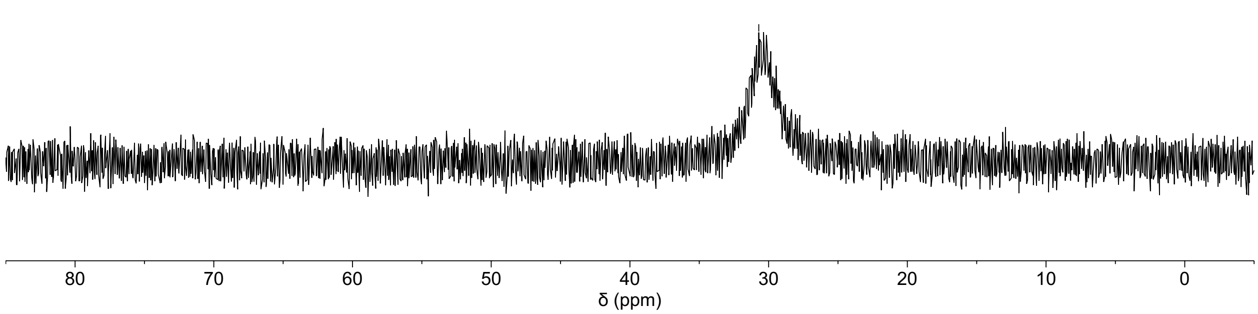

^{1}H, $CDCl_3$, 600 MHz

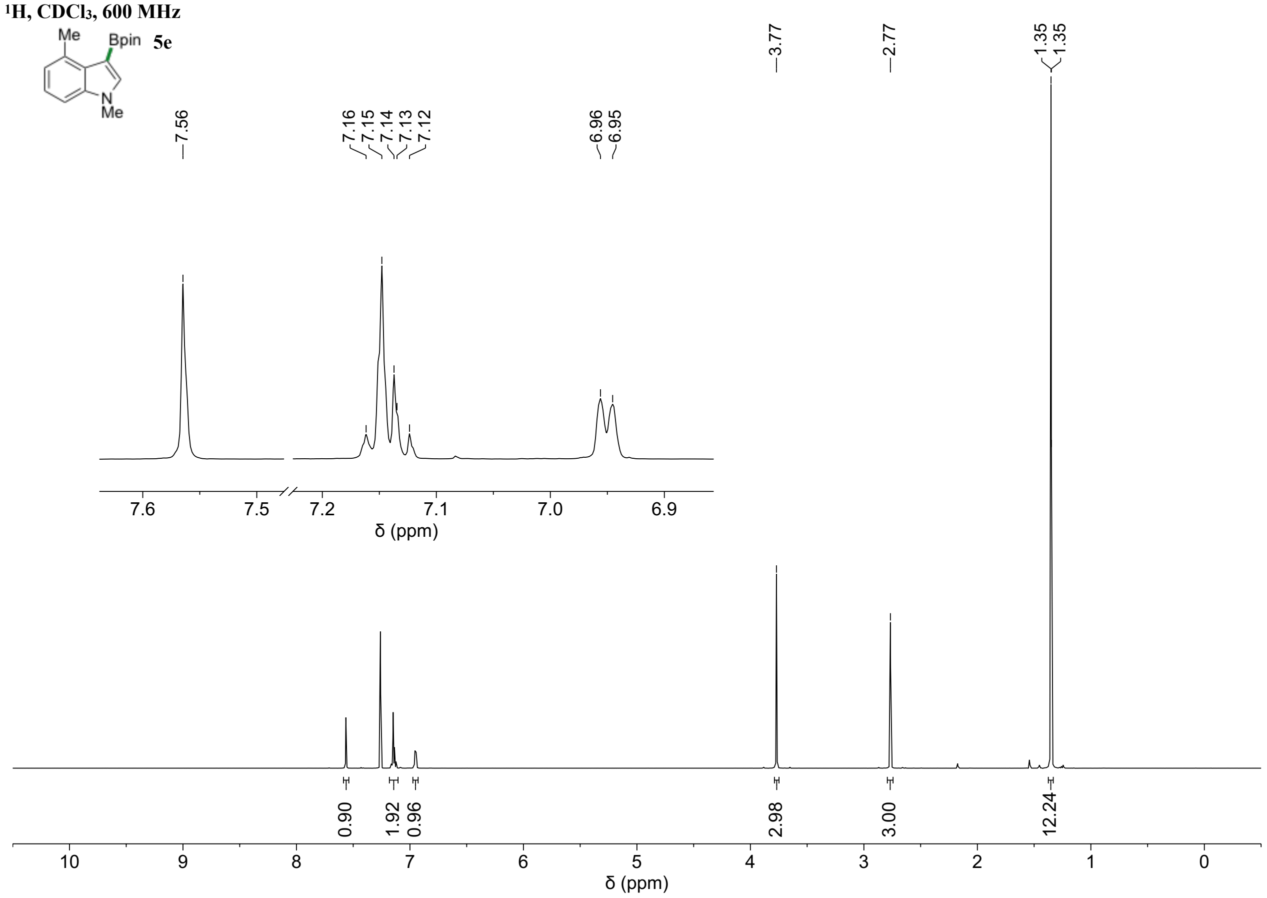

^{13}C, $CDCl_3$, 151 MHz

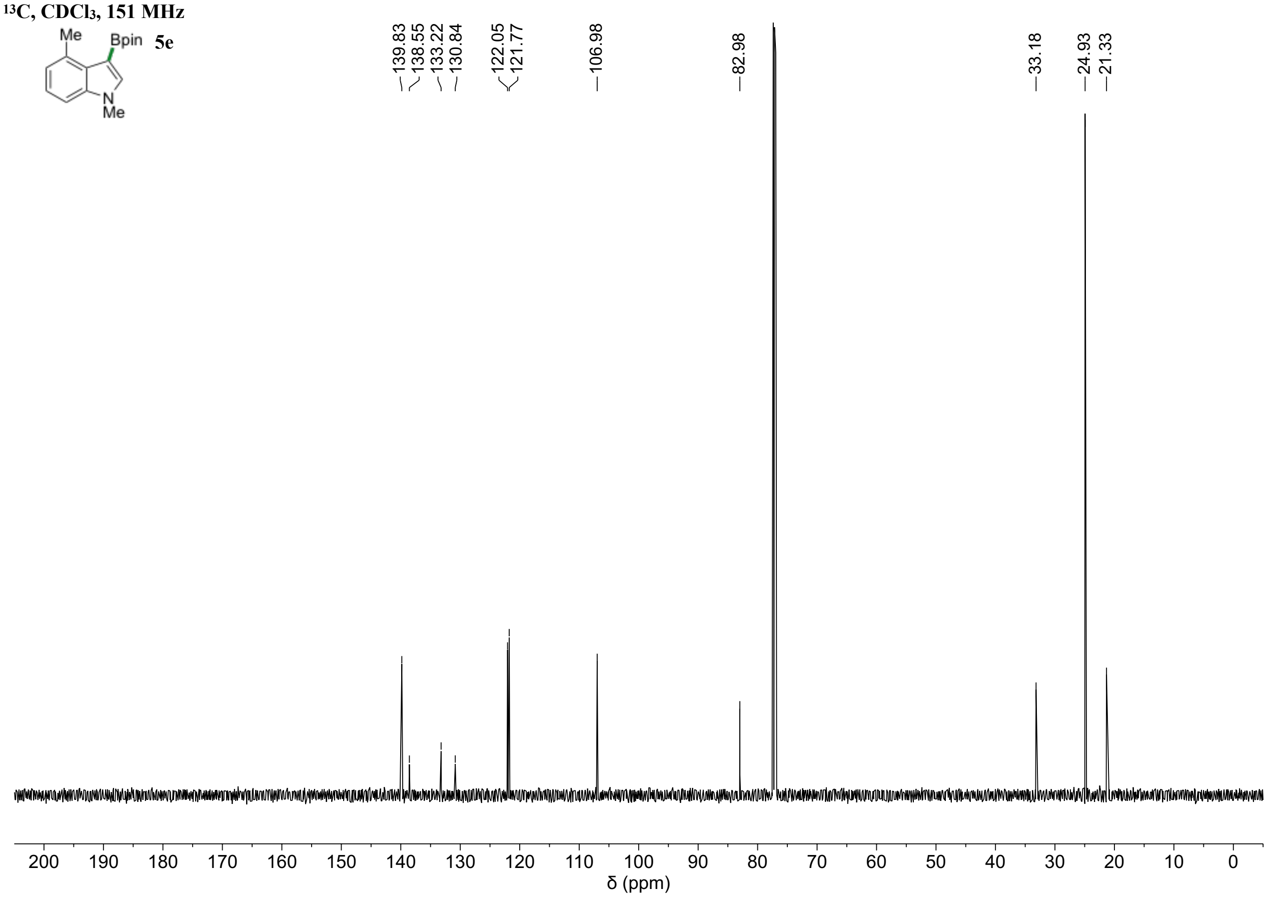

^{11}B, $CDCl_3$, 128 MHz

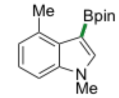

5e

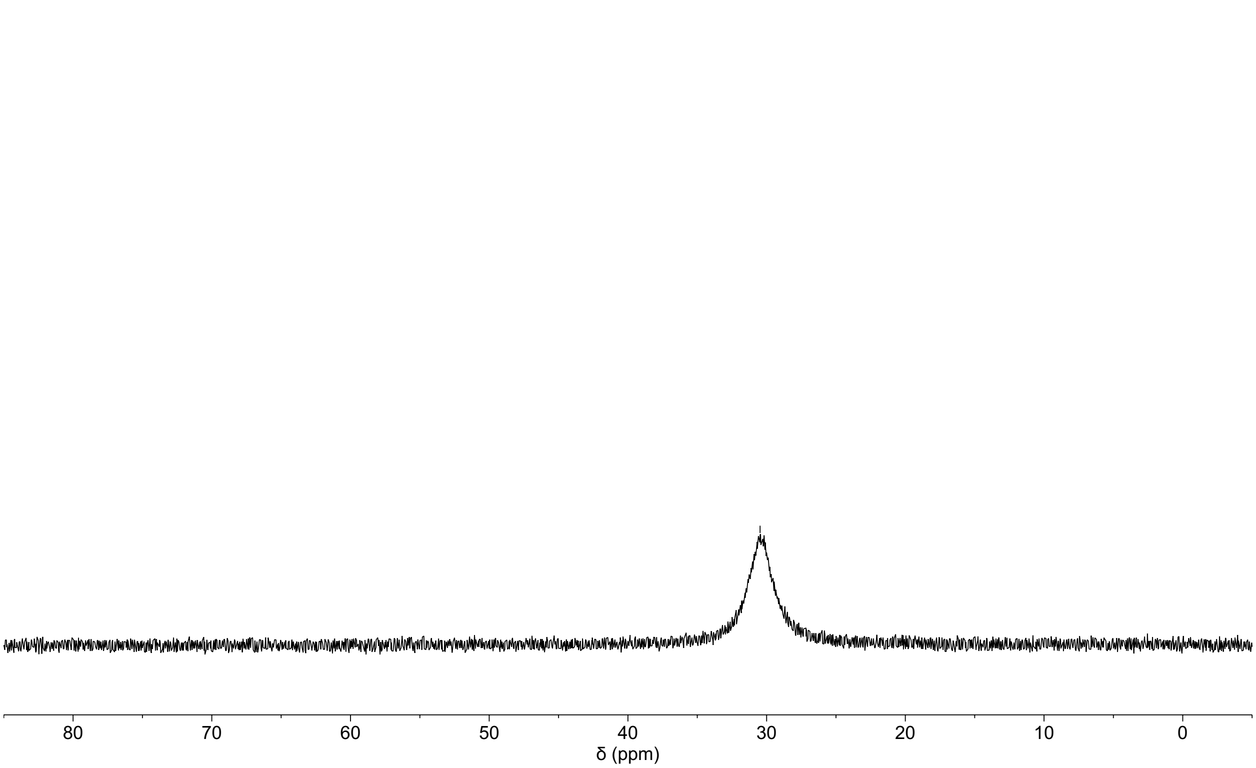

^{1}H, $CDCl_3$, 600 MHz

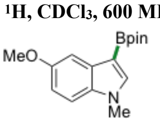

5f

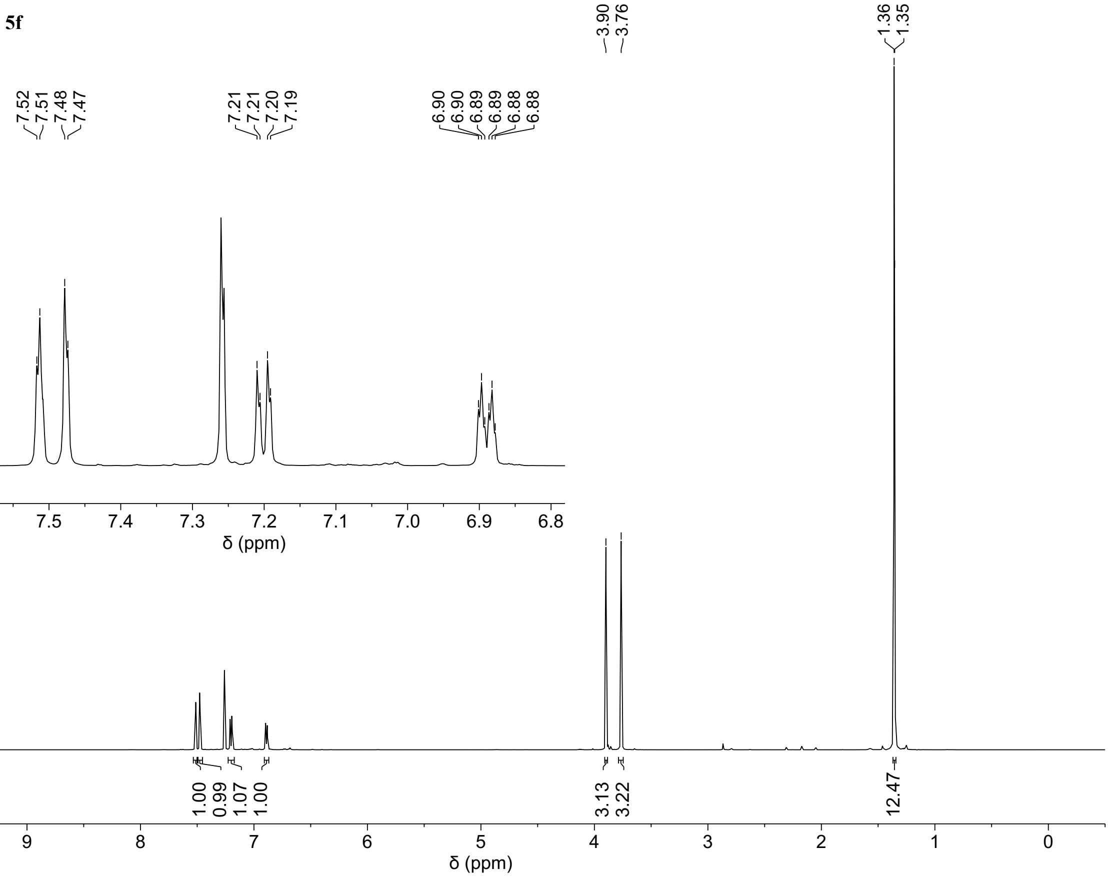

^{13}C, $CDCl_3$, 151 MHz

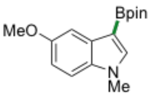

5f

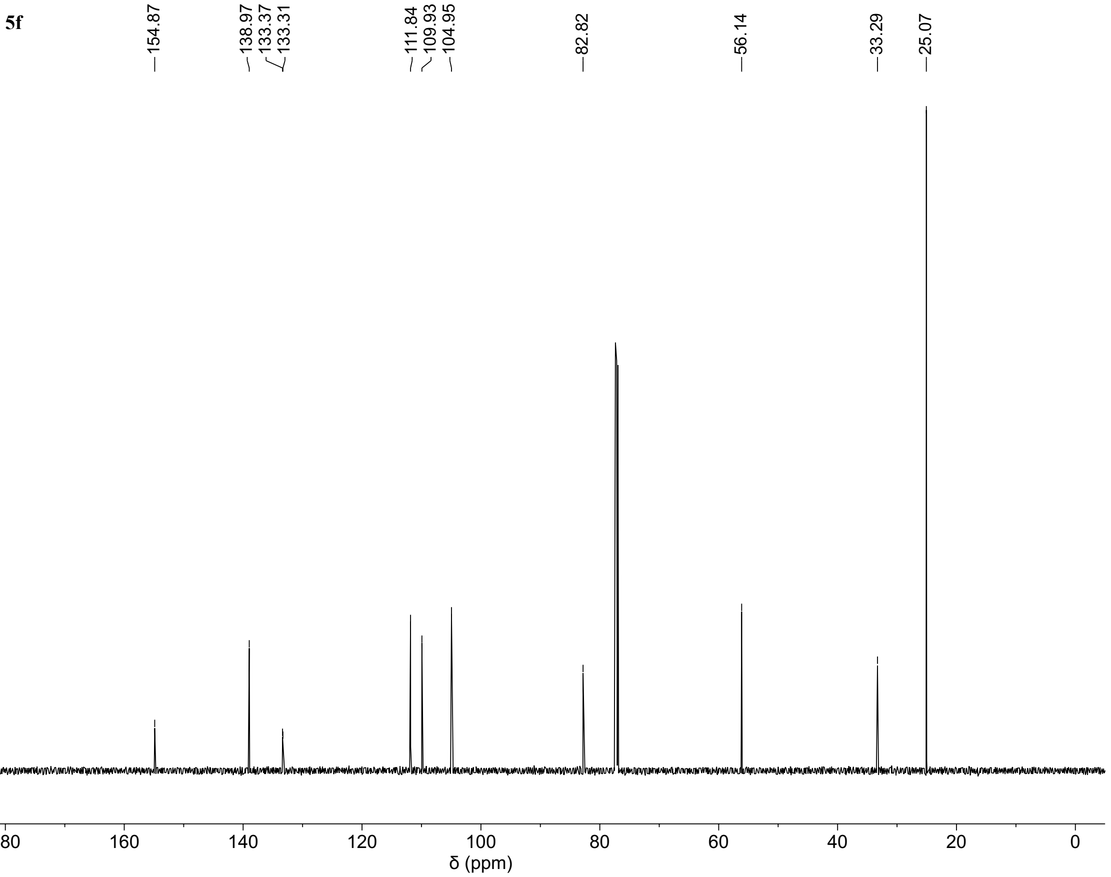

^{11}B, $CDCl_3$, 128 MHz

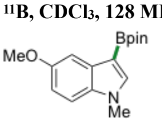

5f

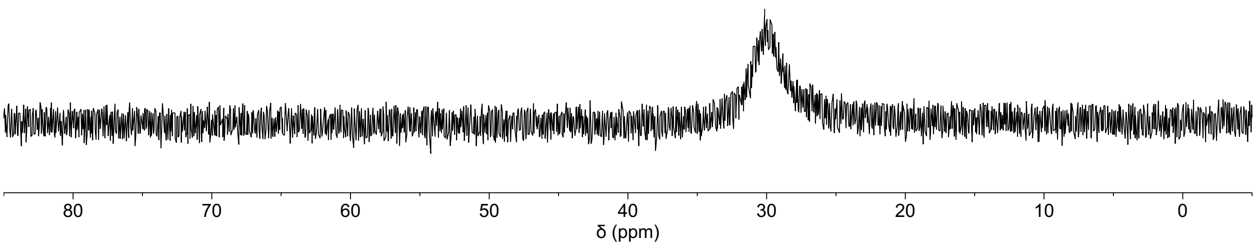

^1H, $CDCl_3$, 600 MHz

5g

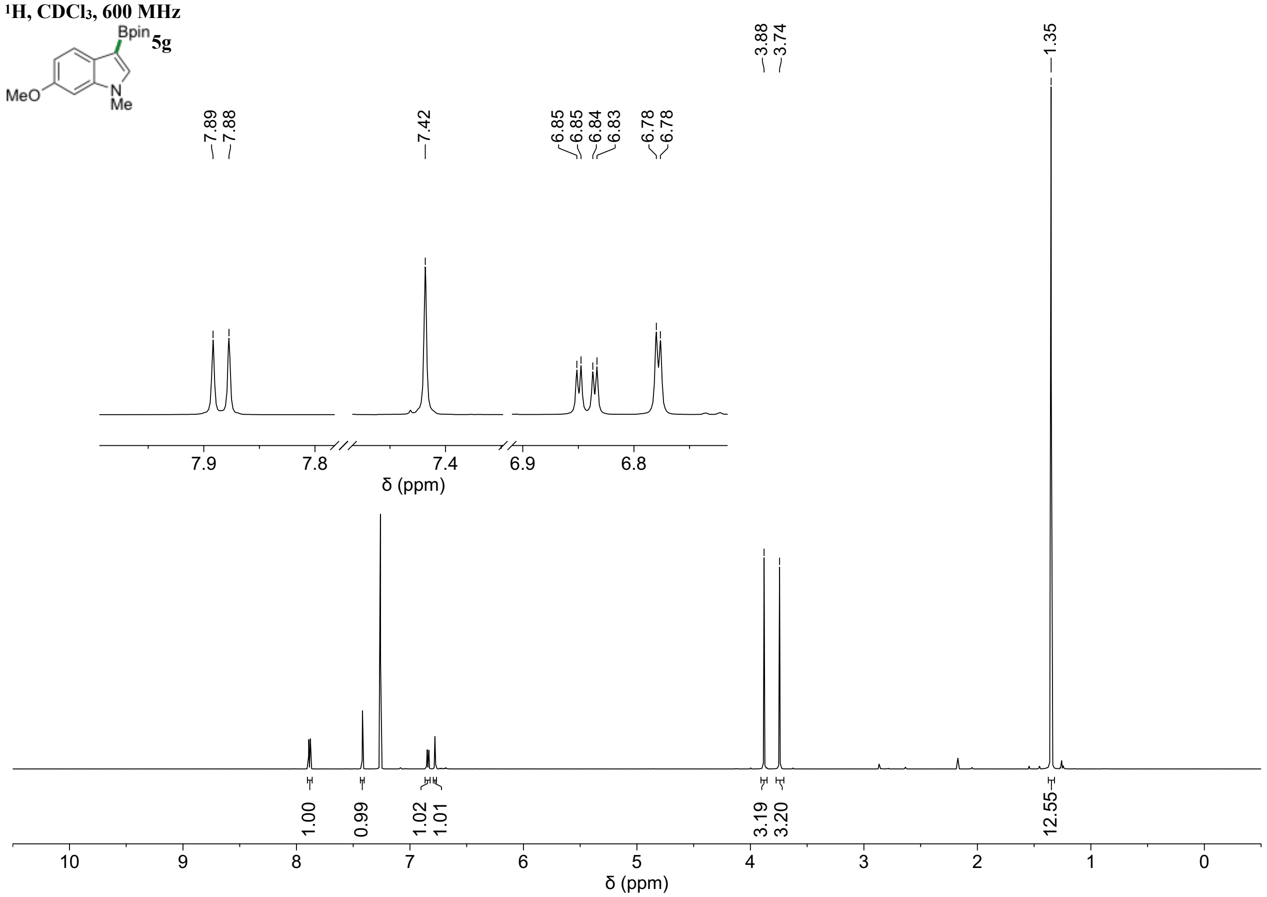

^{13}C, $CDCl_3$, 151 MHz

5g

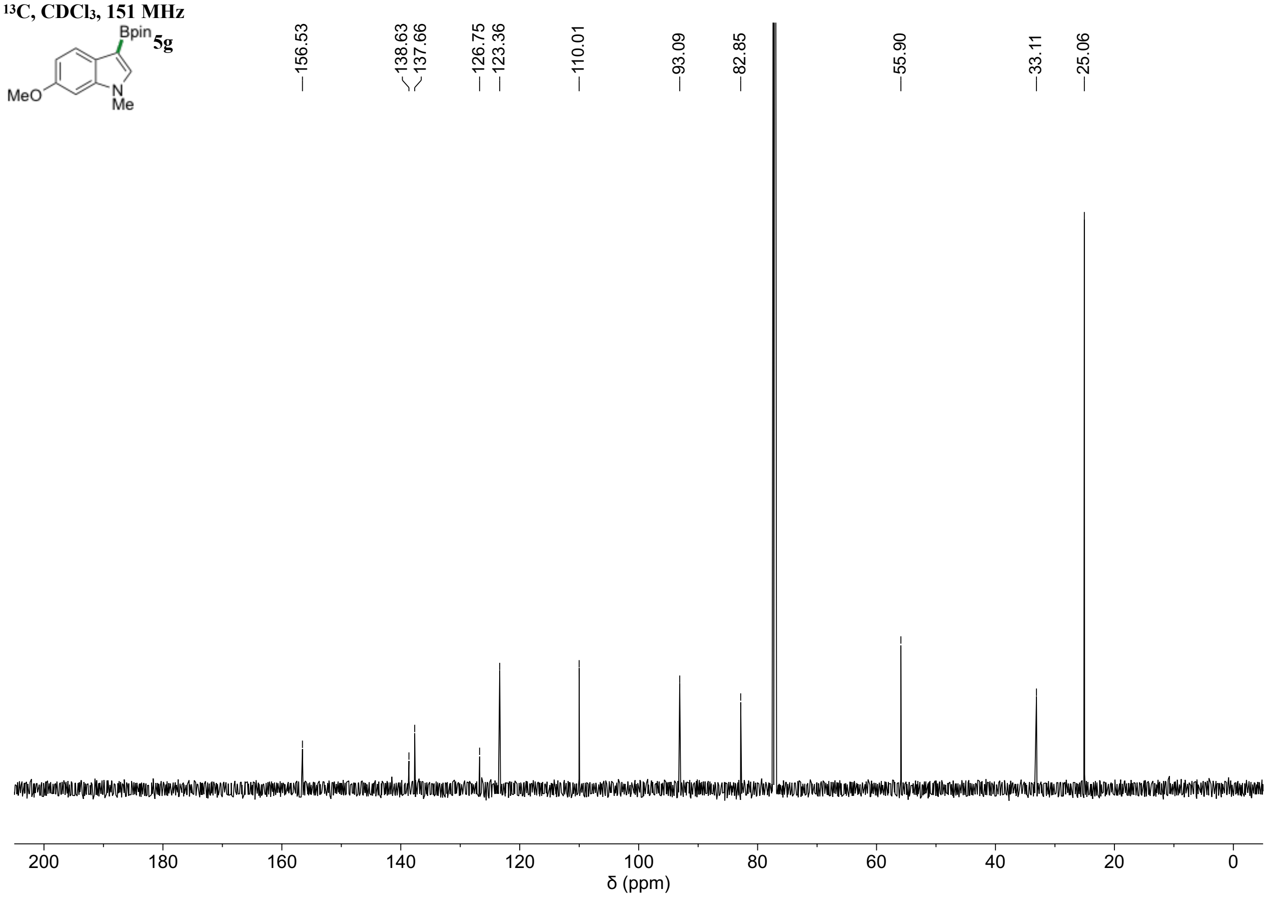

^{11}B, $CDCl_3$, 128 MHz

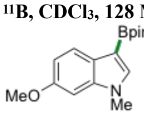

5g

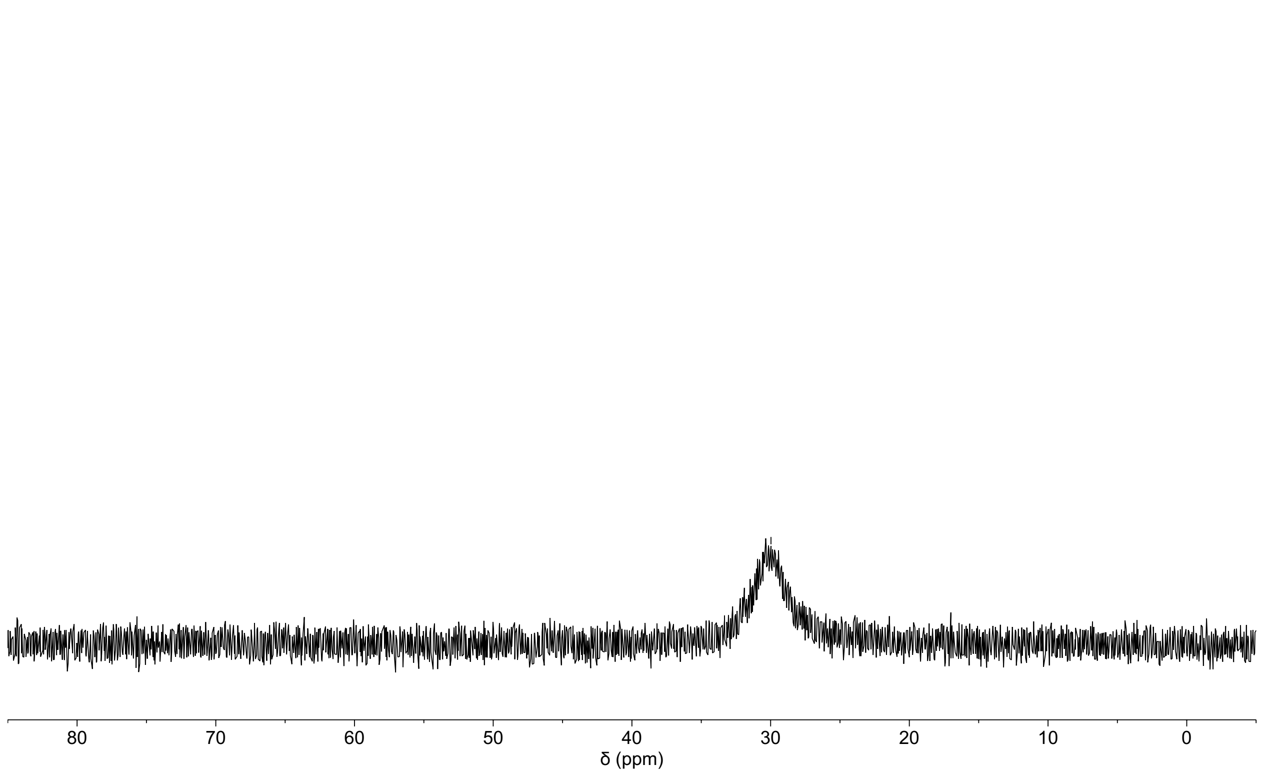

^{1}H, $CDCl_3$, 600 MHz

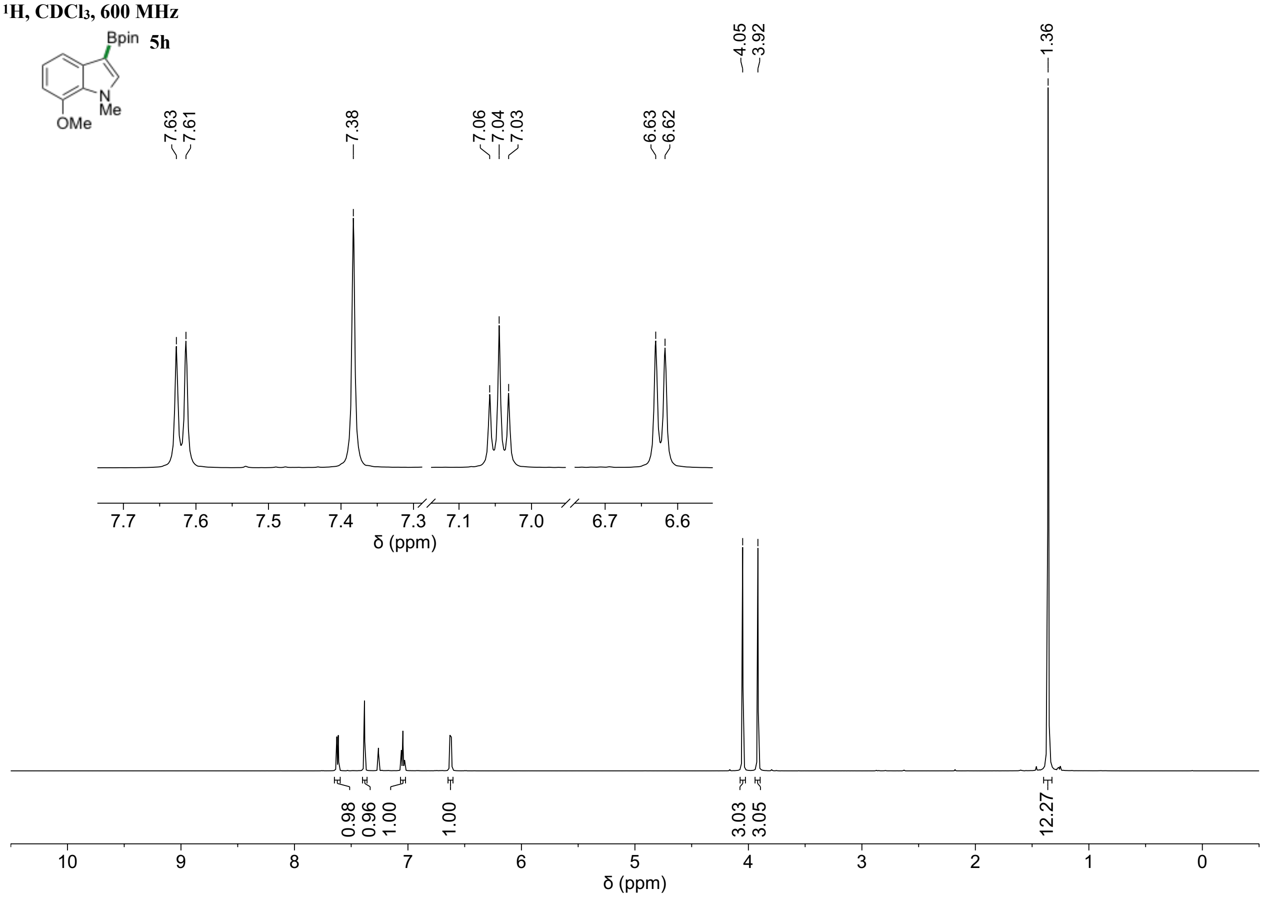

^{13}C, $CDCl_3$, 151 MHz

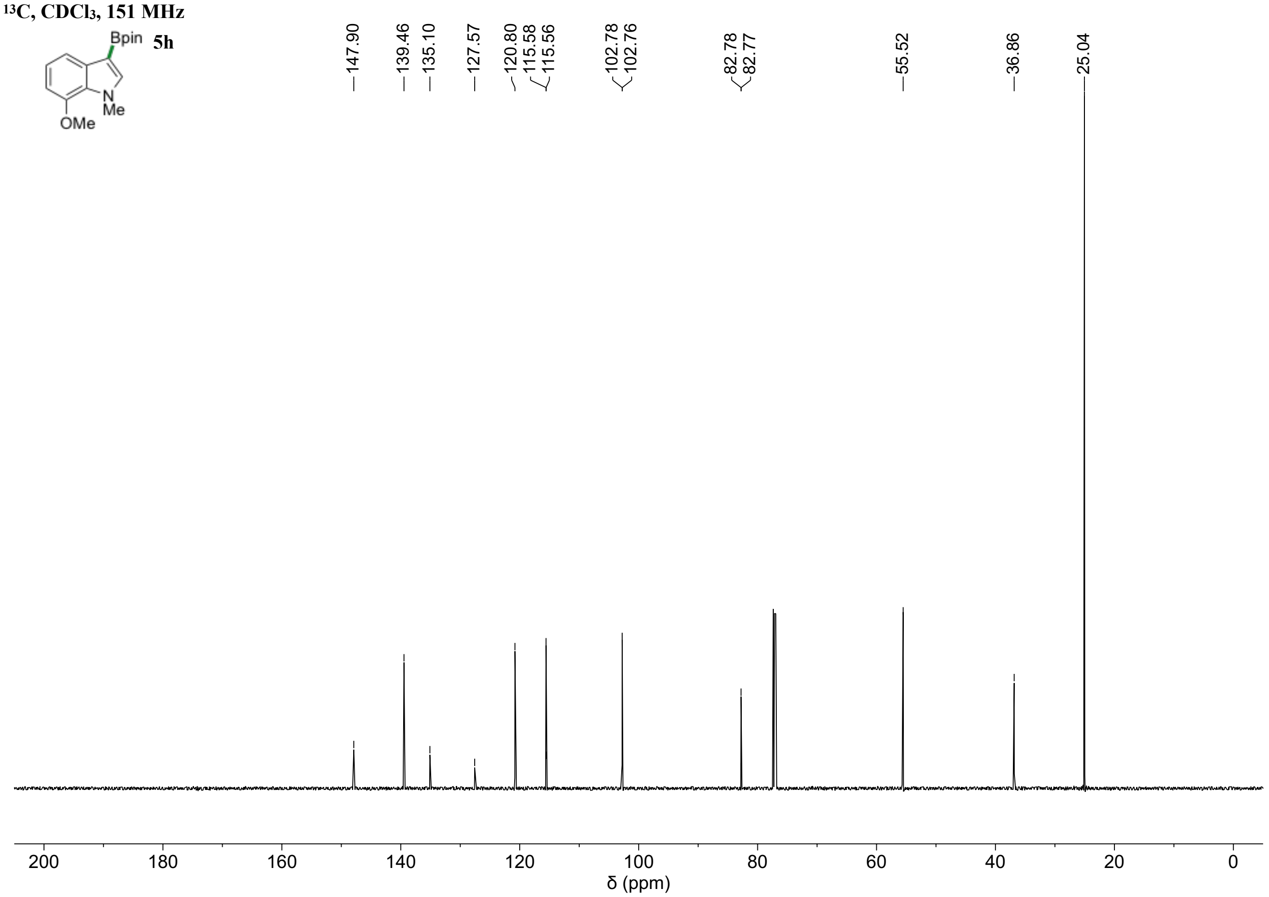

^{11}B, $CDCl_3$, 128 MHz

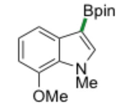

5h

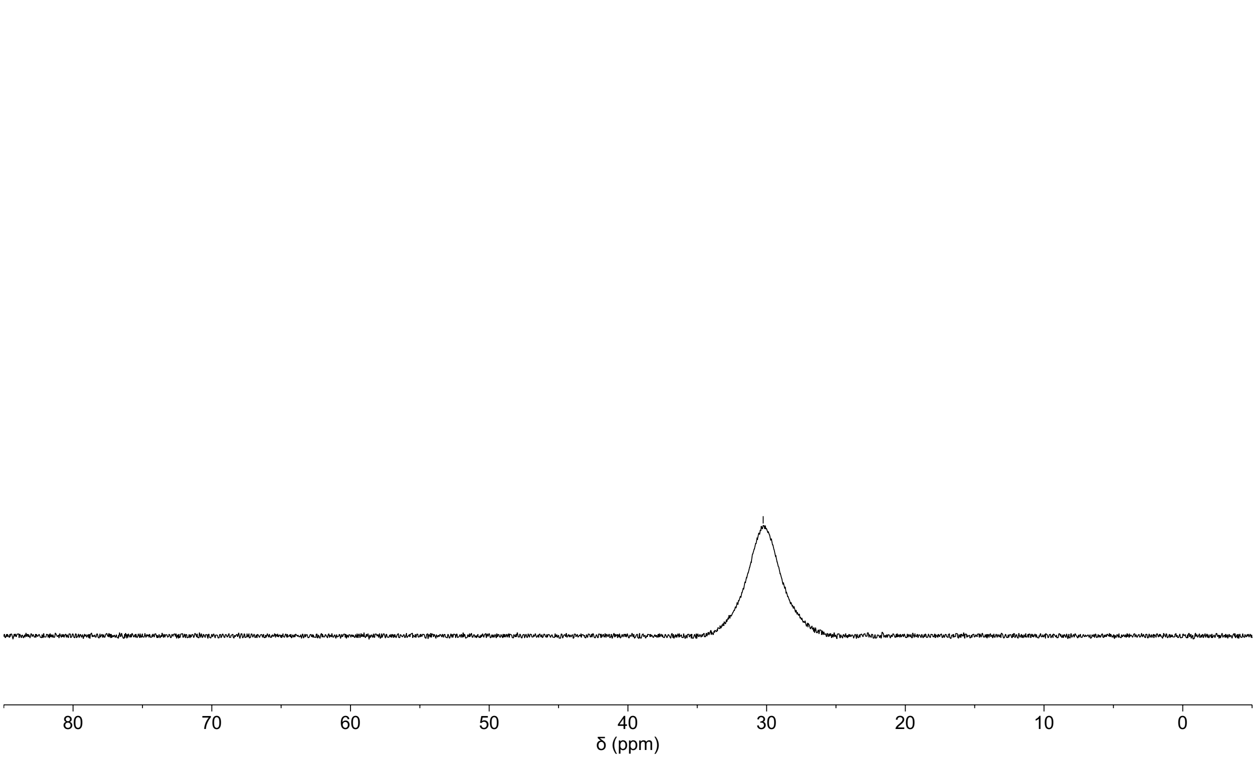

^{1}H, $CDCl_3$, 600 MHz

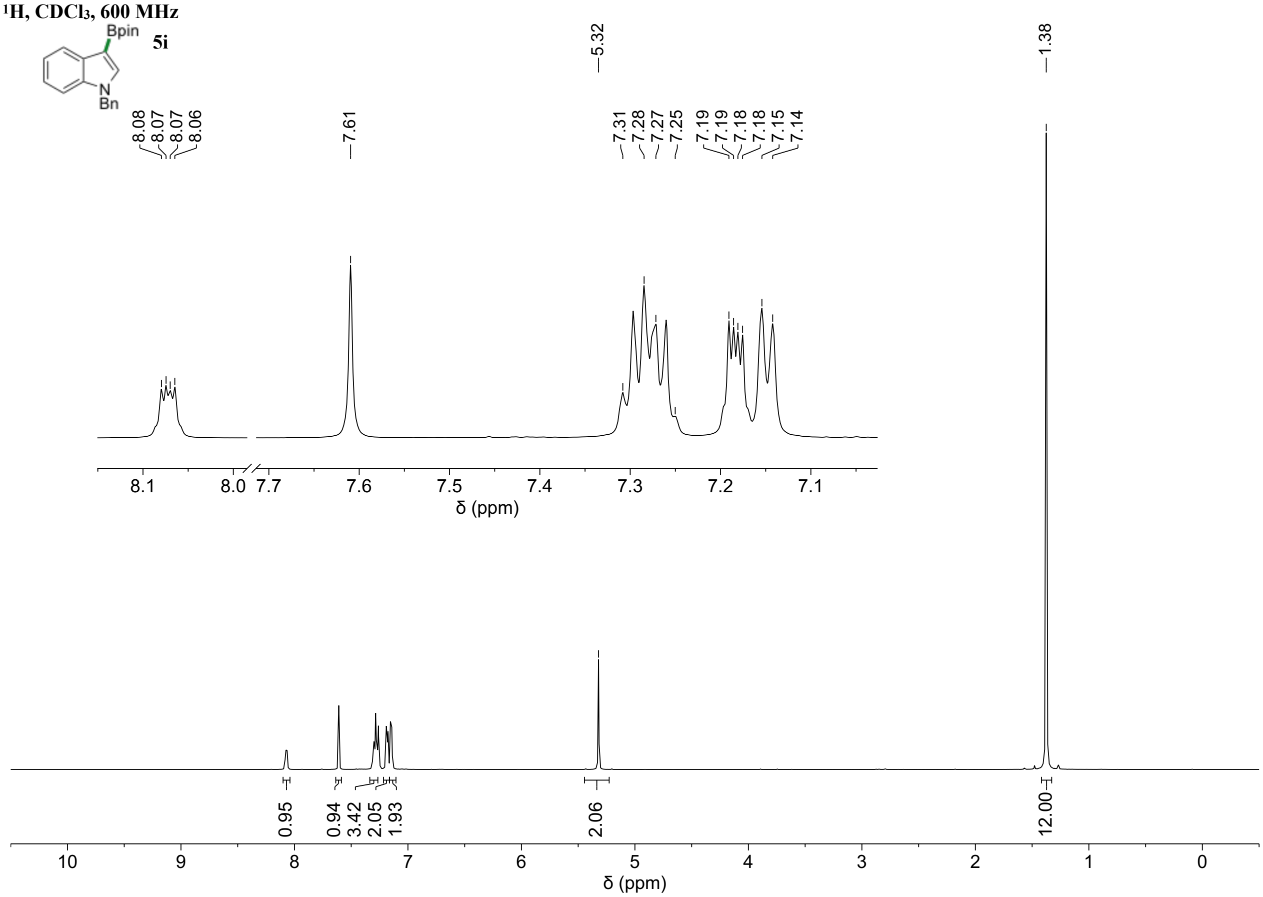

^{13}C, $CDCl_3$, 151 MHz

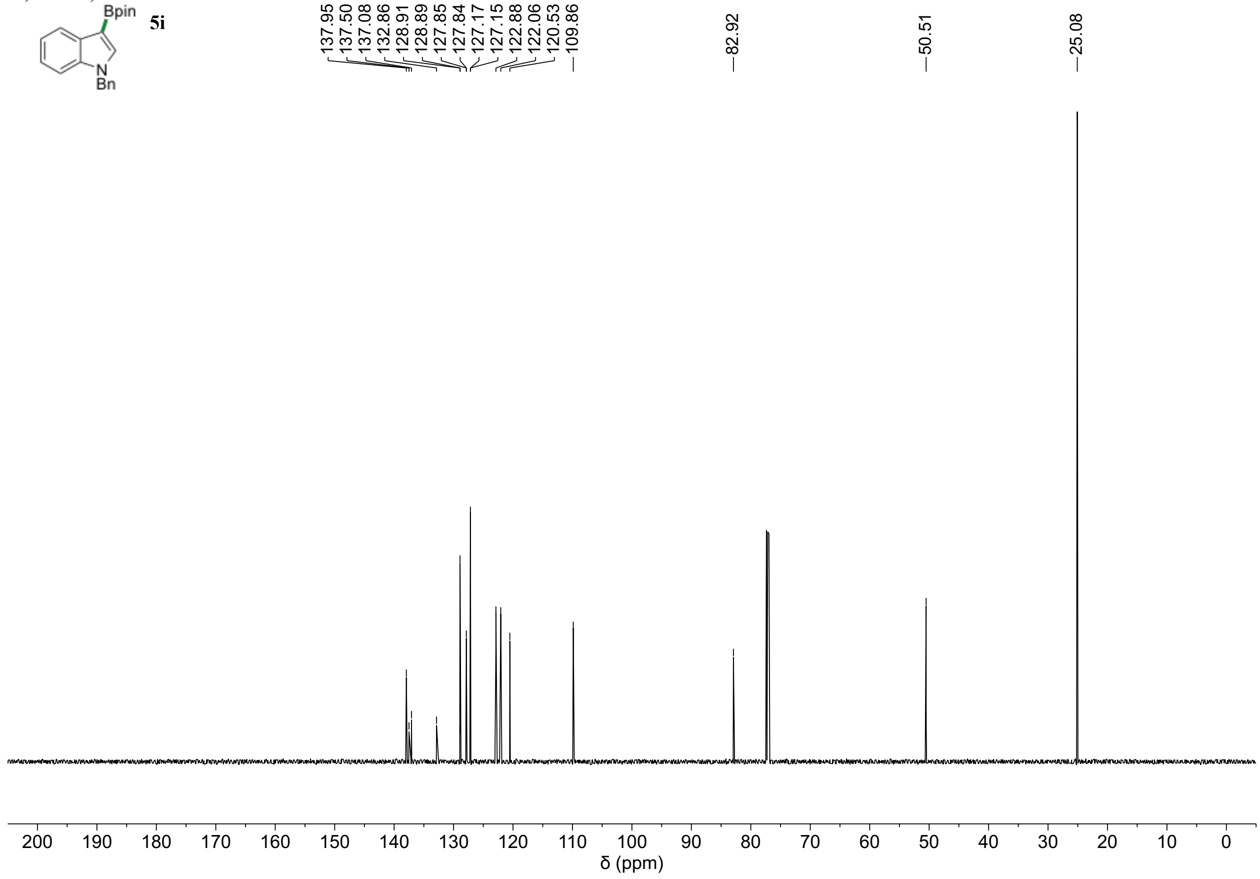

^{11}B, $CDCl_3$, 128 MHz

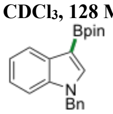

5i

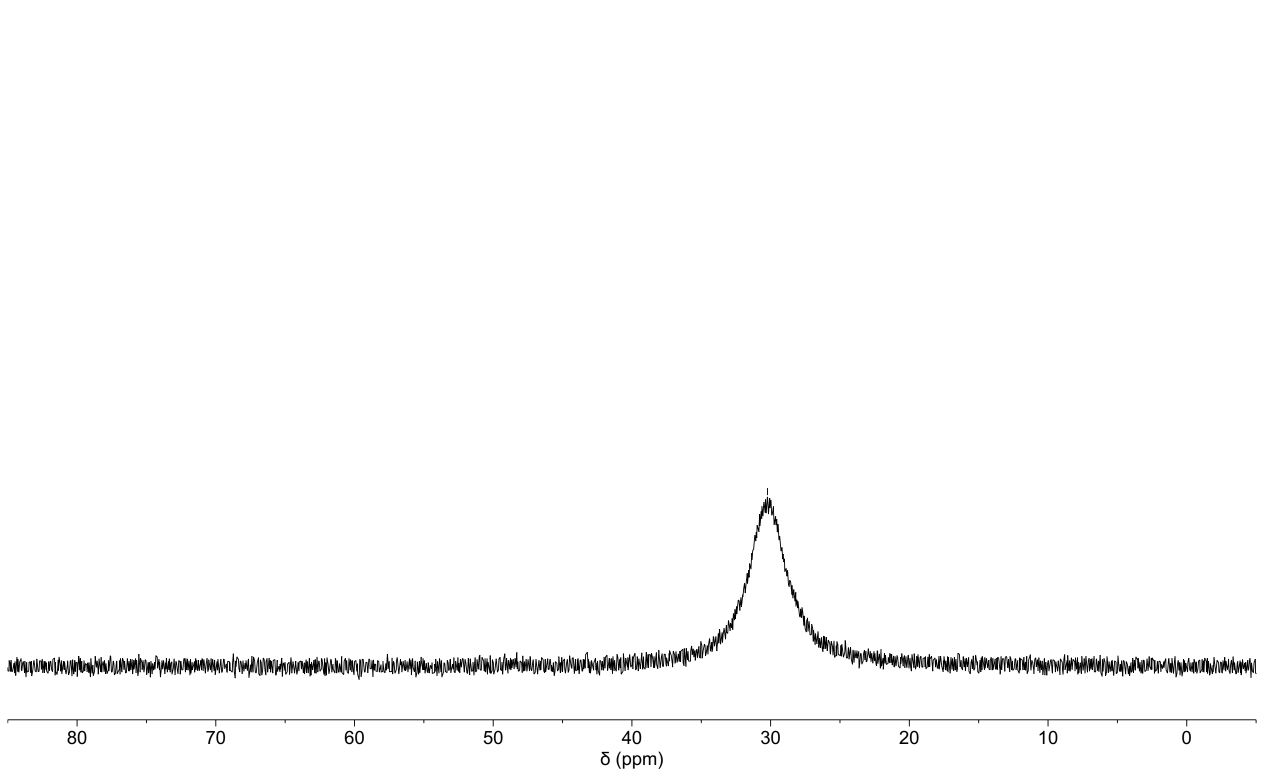

^{1}H, CDCl$_3$, 600 MHz

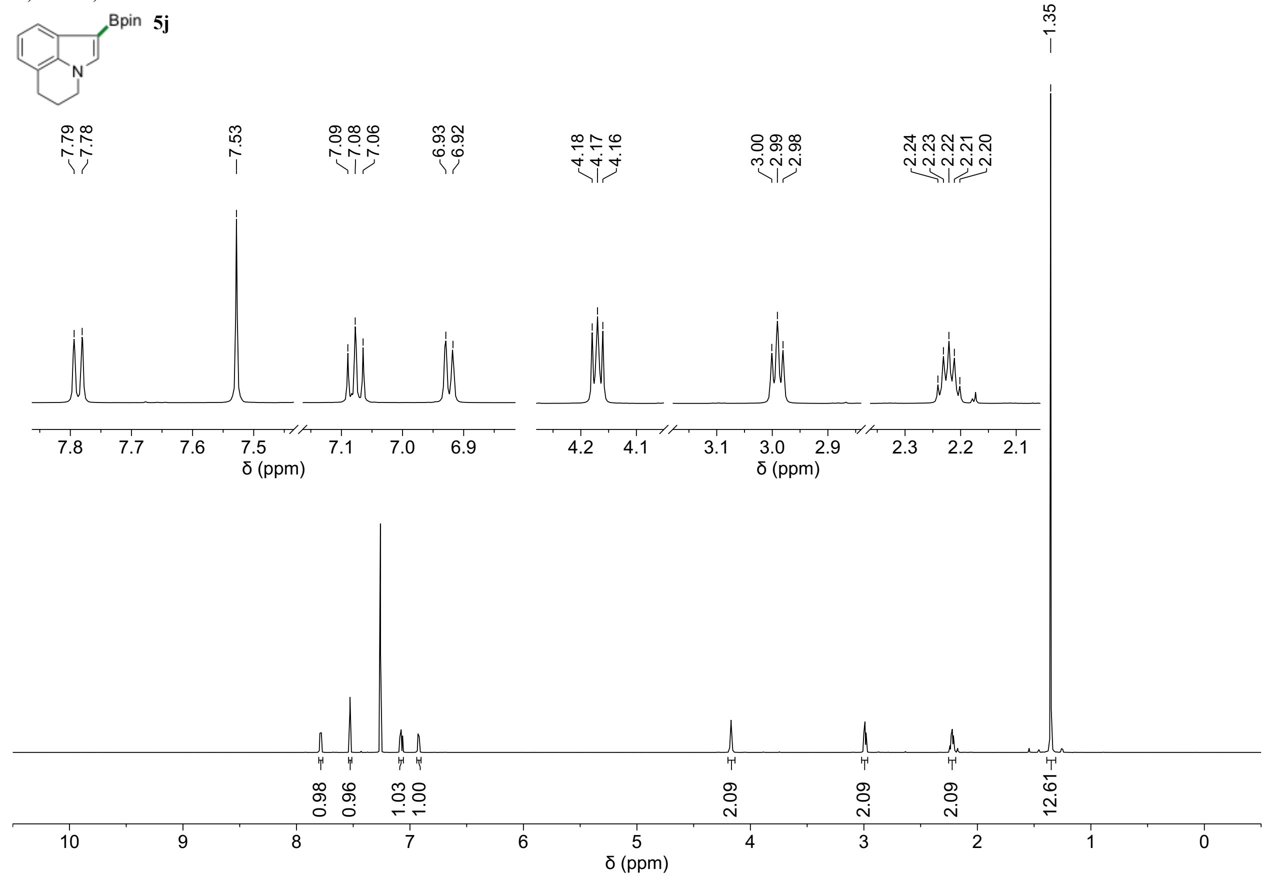

^{13}C, CDCl$_3$, 151 MHz

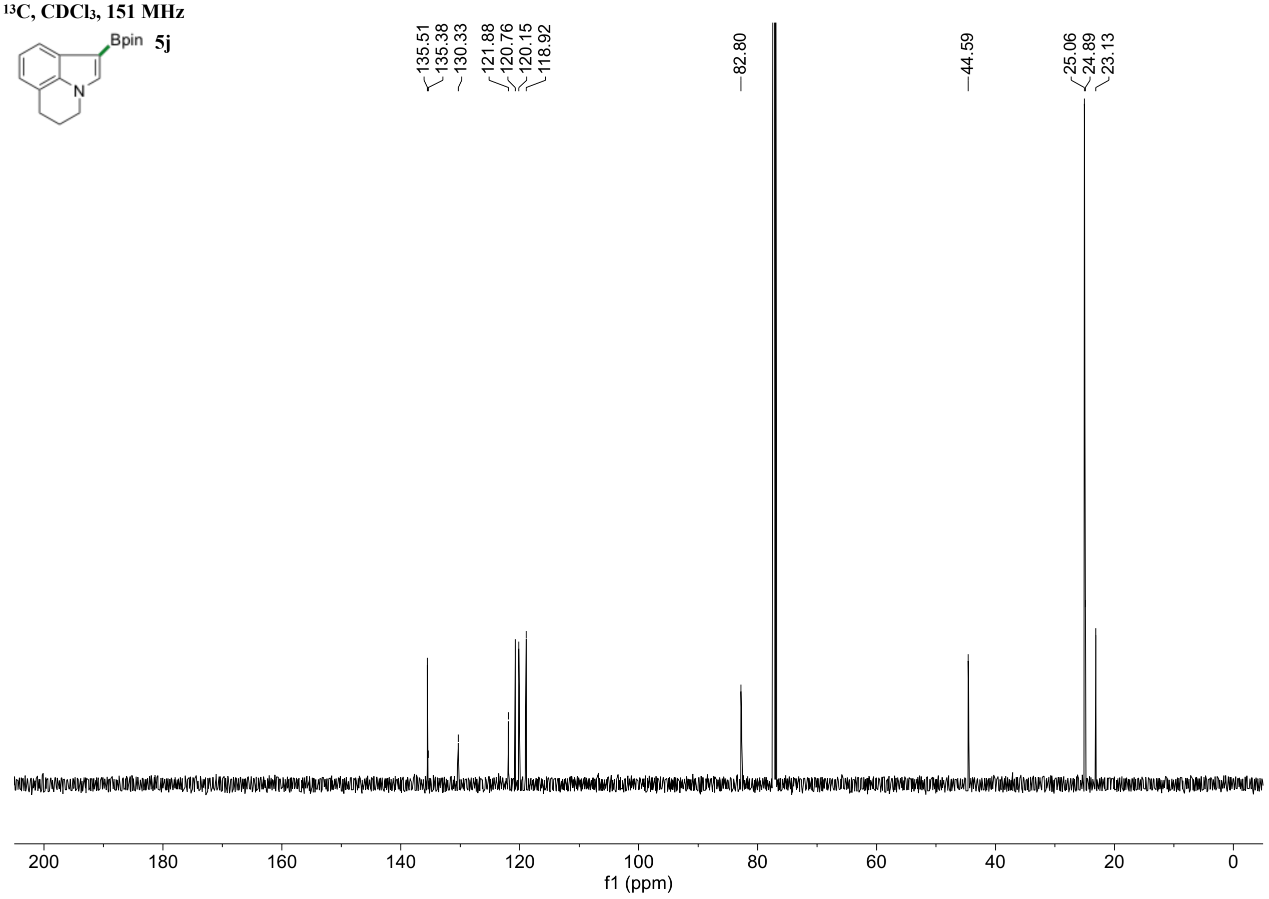

^{11}B, CDCl3, 128 MHz

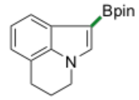

5j

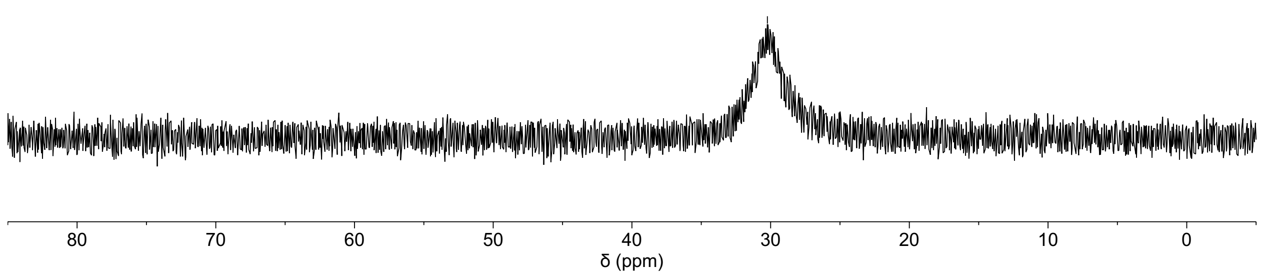

¹H, CDCl₃, 600 MHz

5k

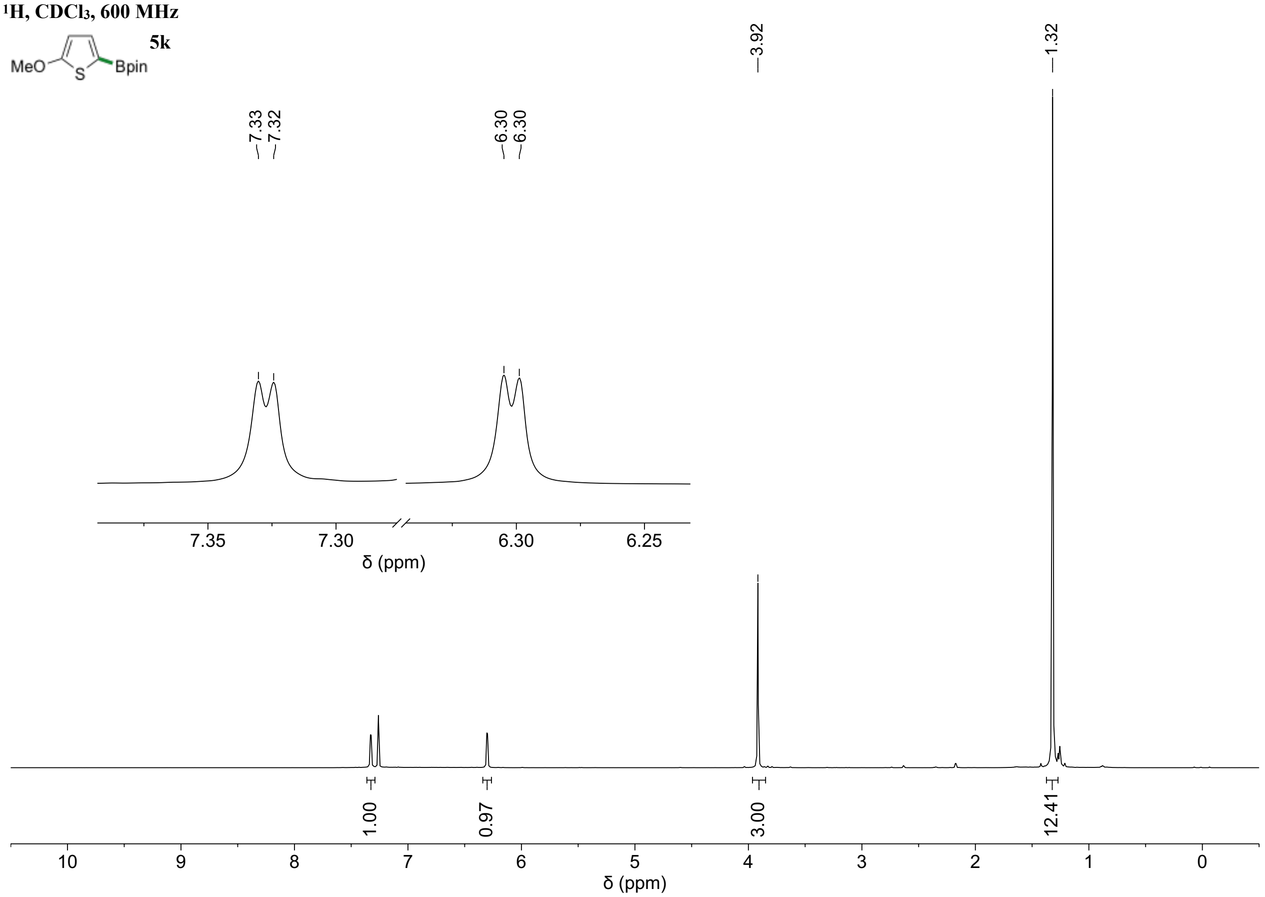

¹³C, CDCl₃, 151 MHz

5k

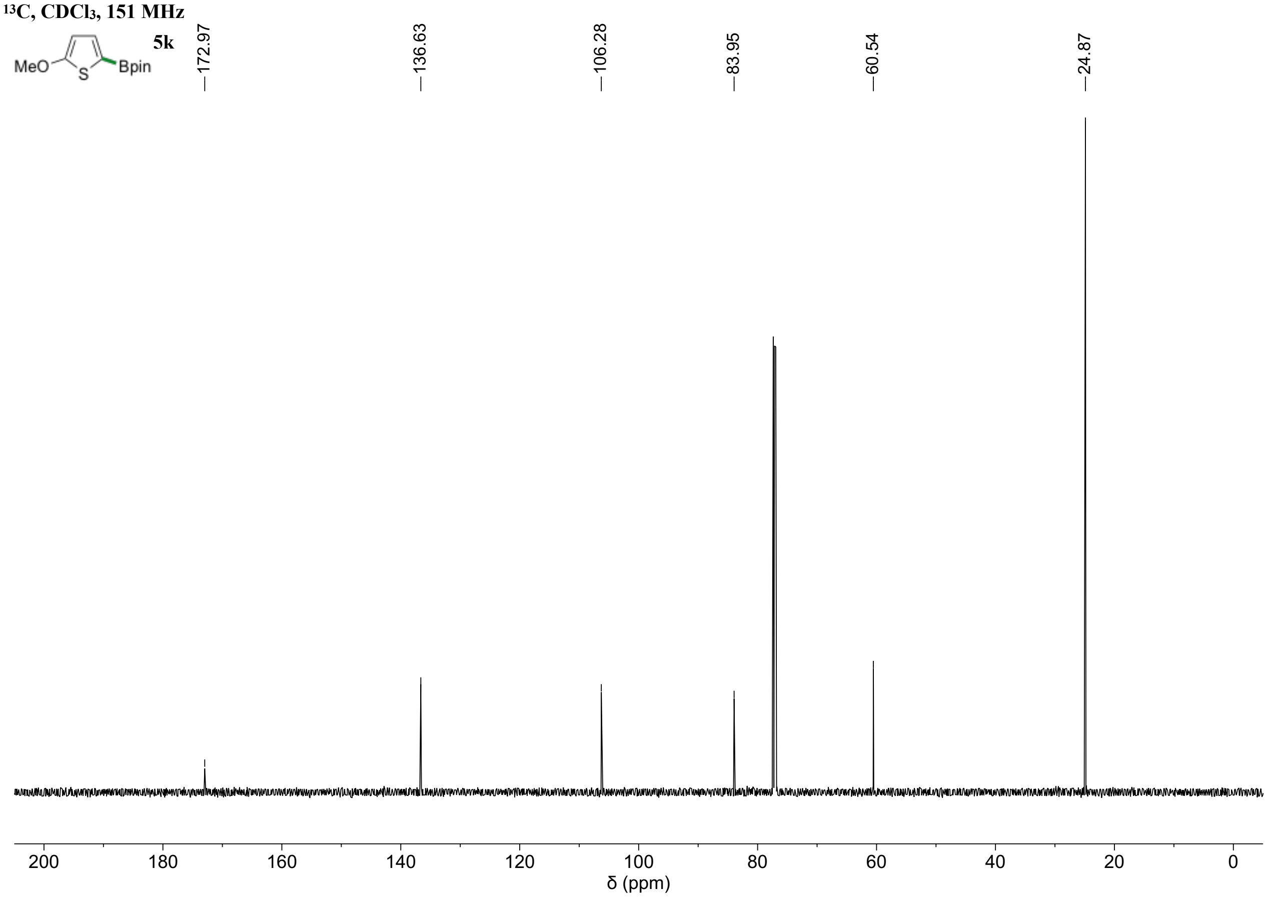

^{11}B, CDCl$_3$, 128 MHz

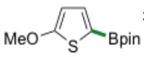

5k

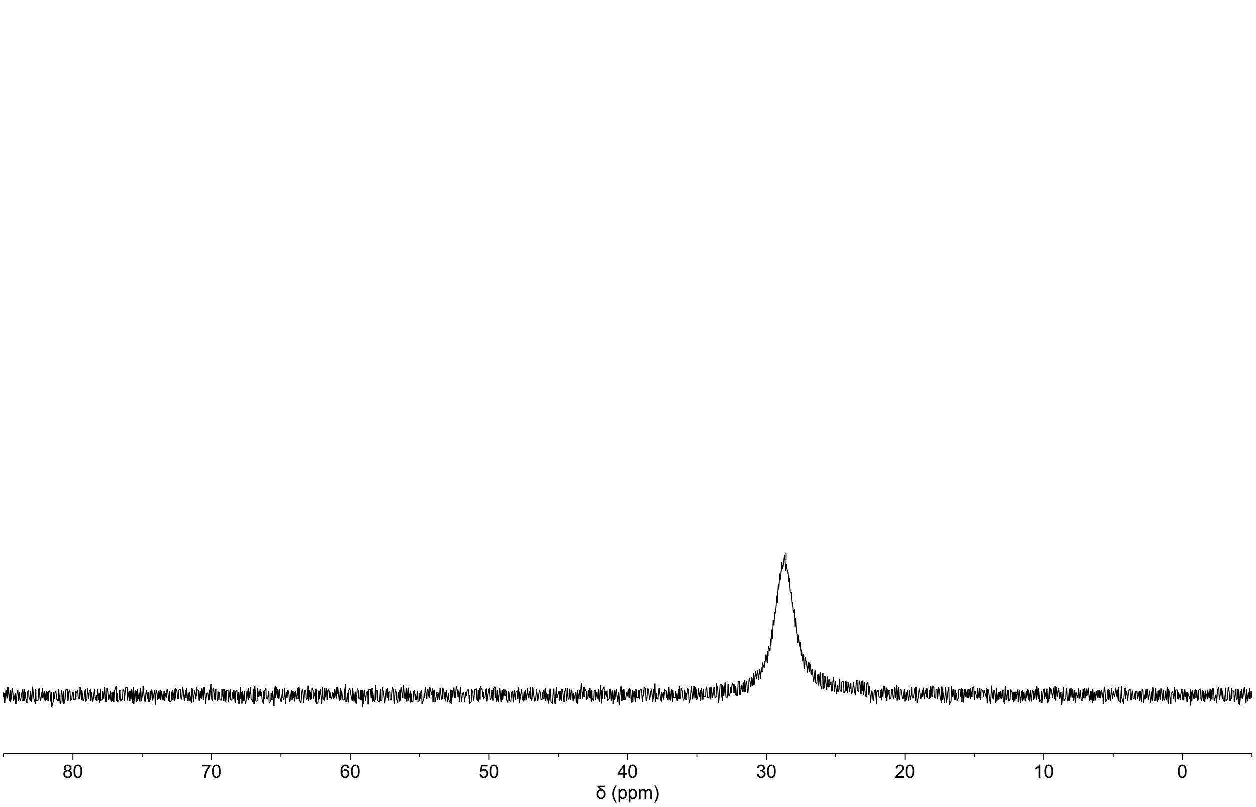

^{1}H, $CDCl_3$, 600 MHz

5m

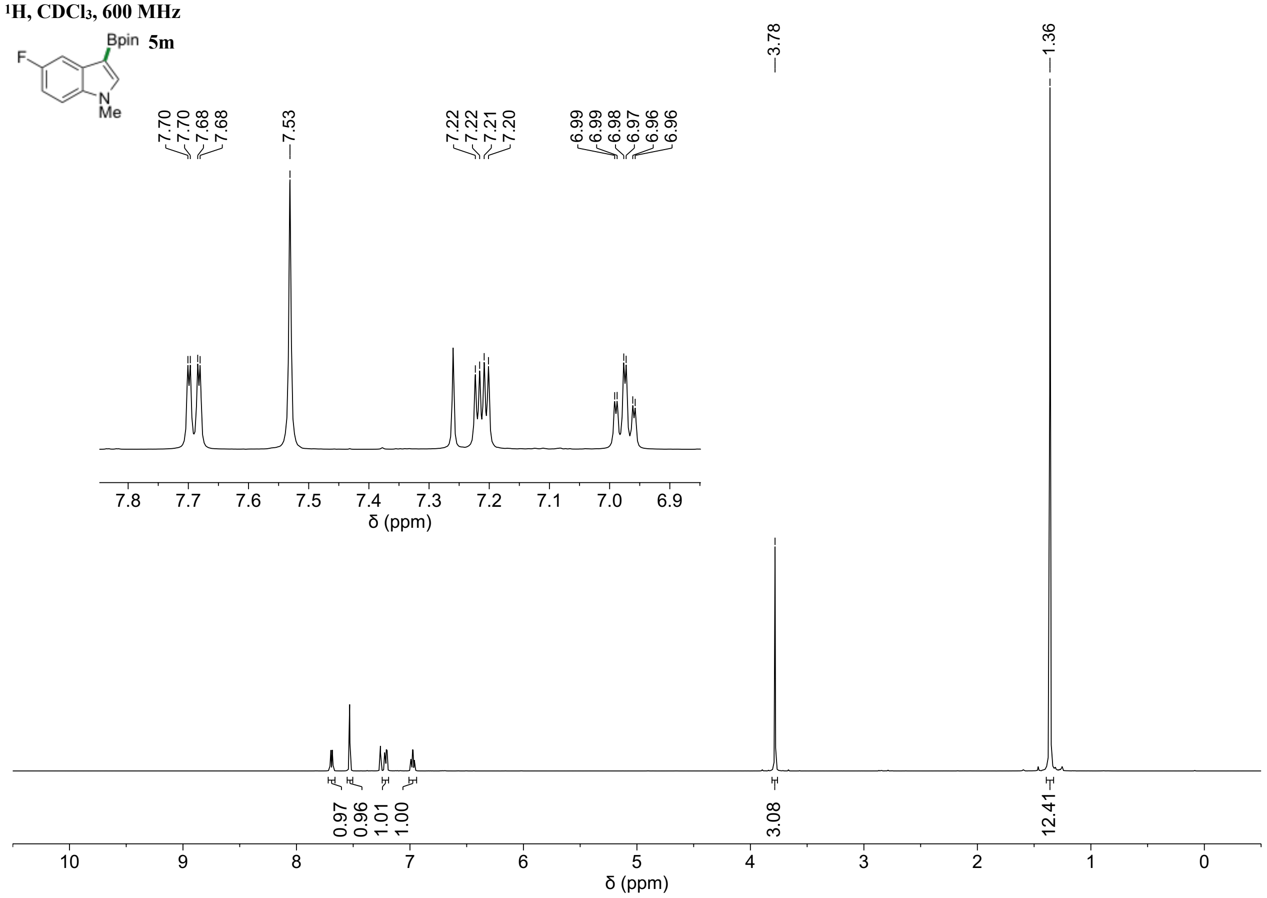

^{13}C, $CDCl_3$, 151 MHz

5m

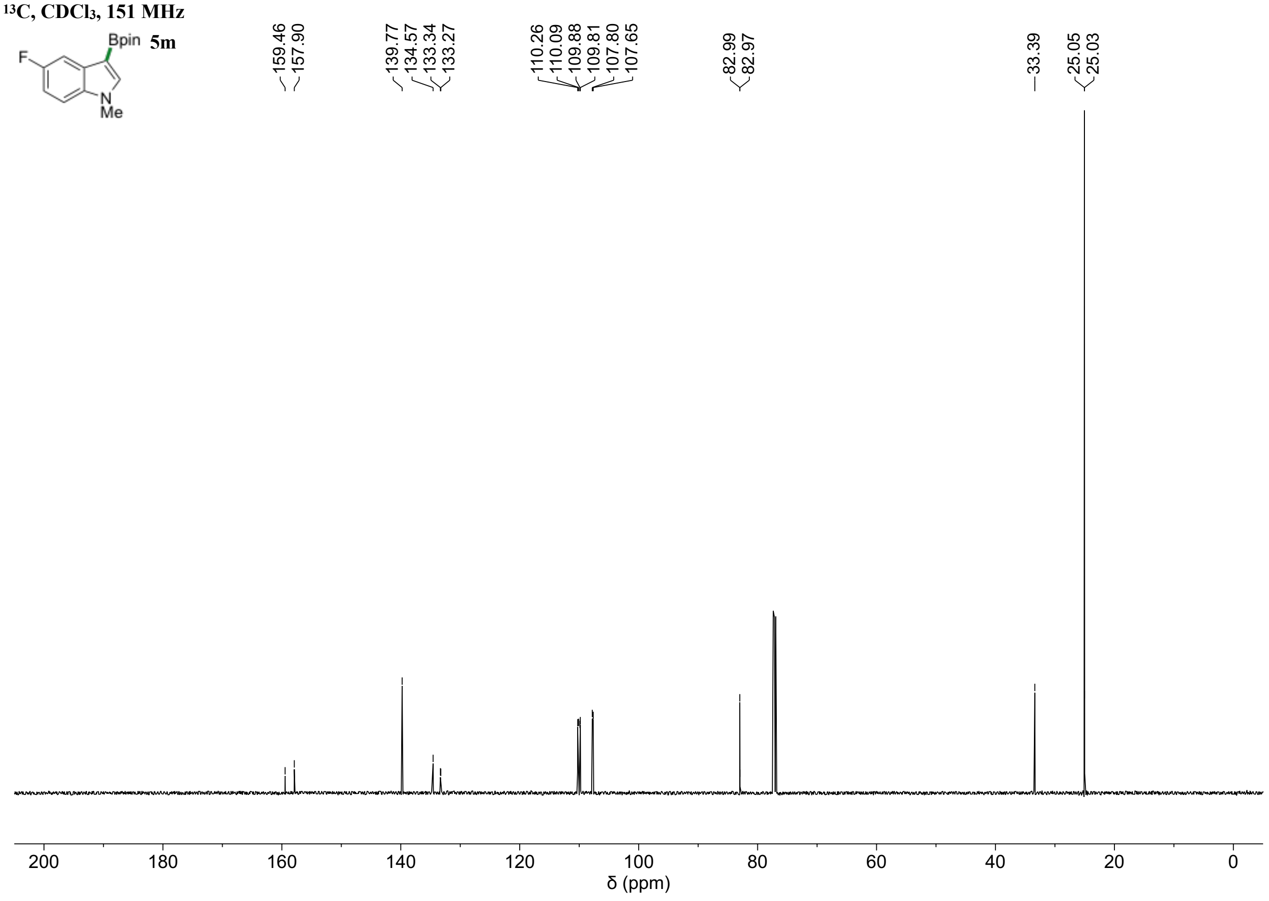

^{11}B, $CDCl_3$, 128 MHz

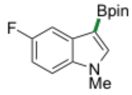

5m

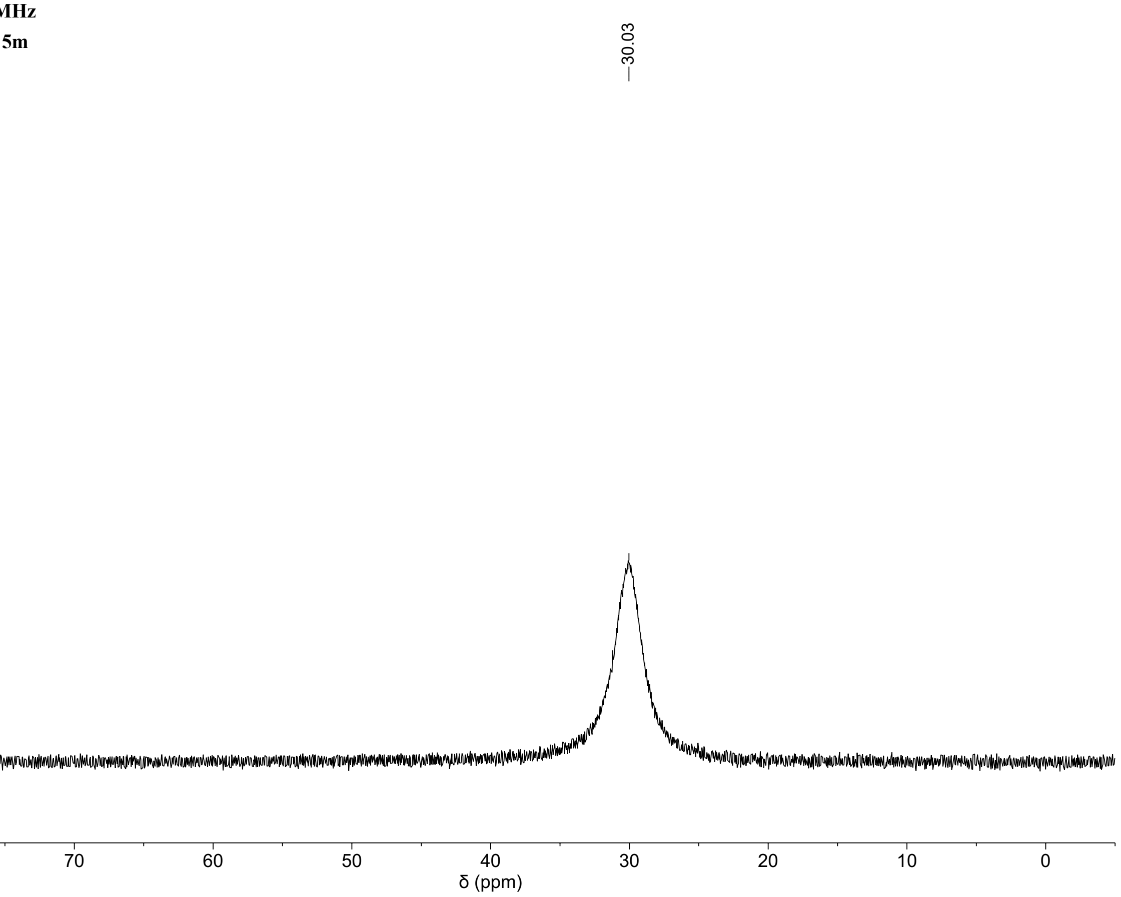

^{19}F, $CDCl_3$, 565 MHz

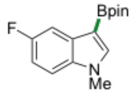

5m

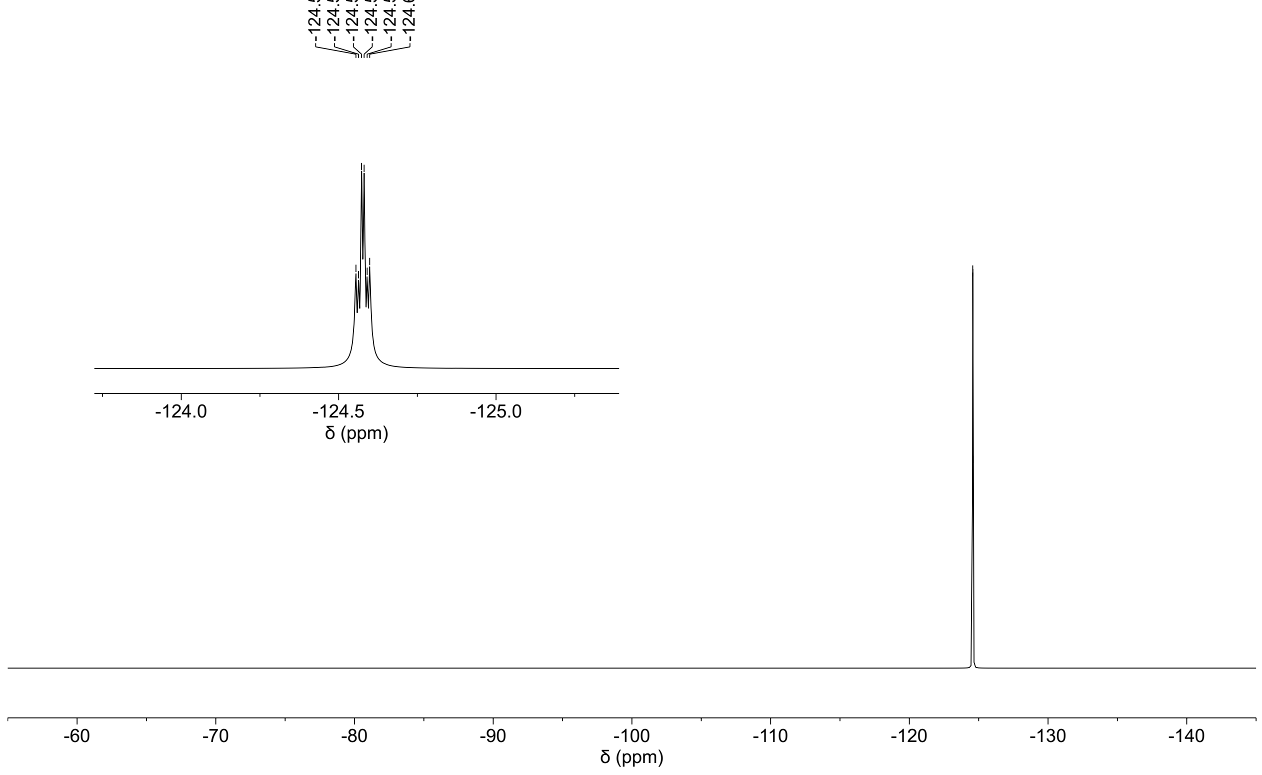

^{1}H, $CDCl_3$, 500 MHz

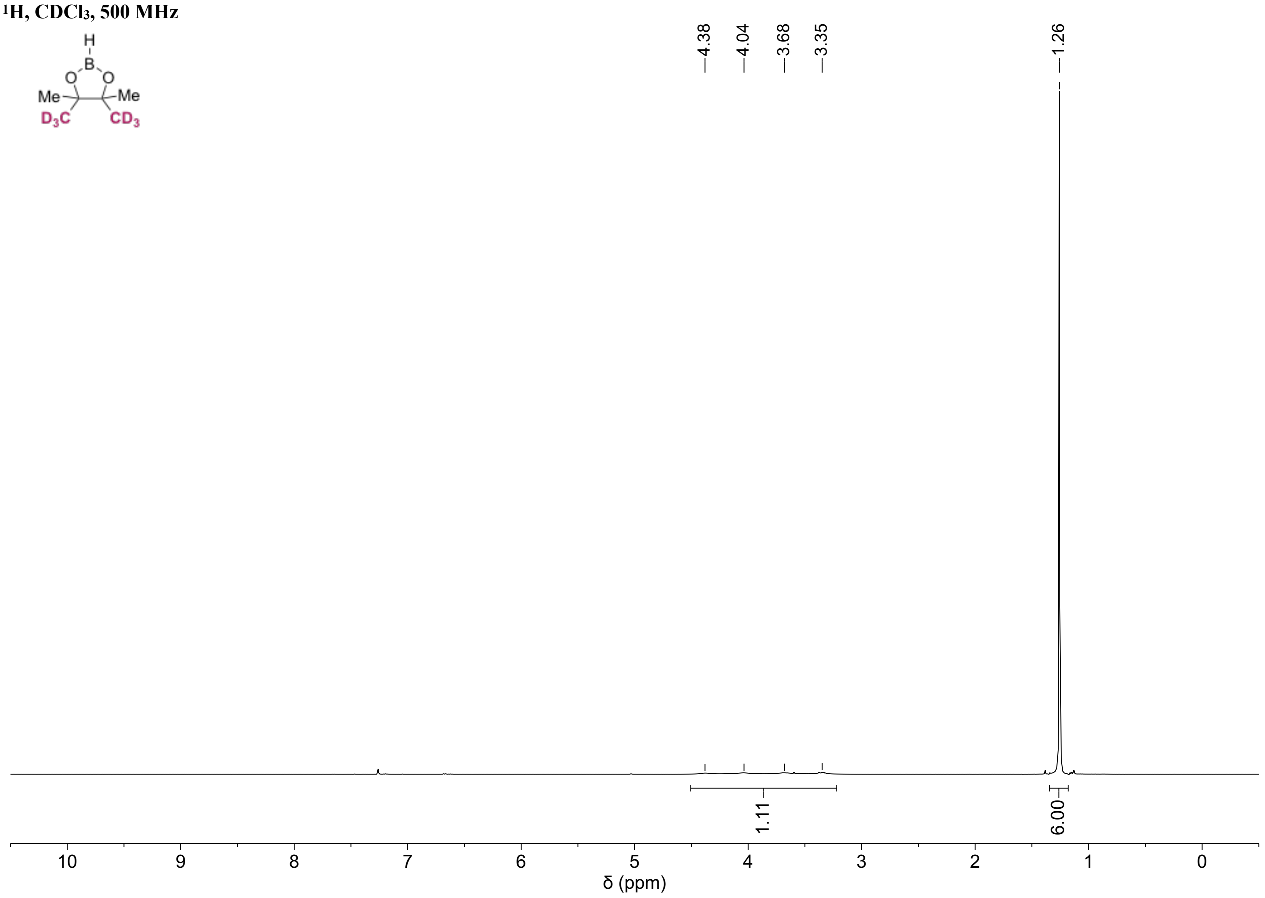

^{2}H, $CHCl_3$, 61 MHz

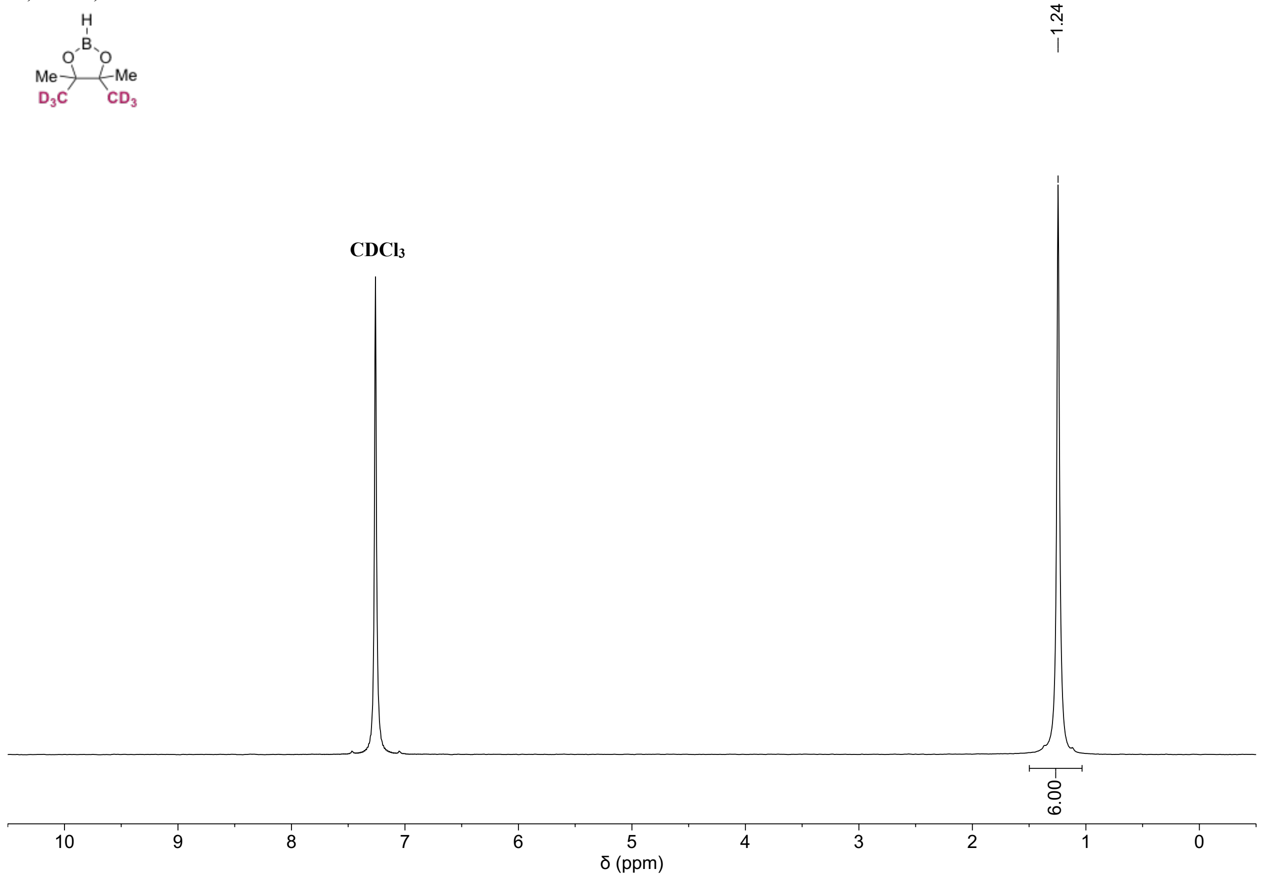

^{13}C, $CDCl_3$, 151 MHz

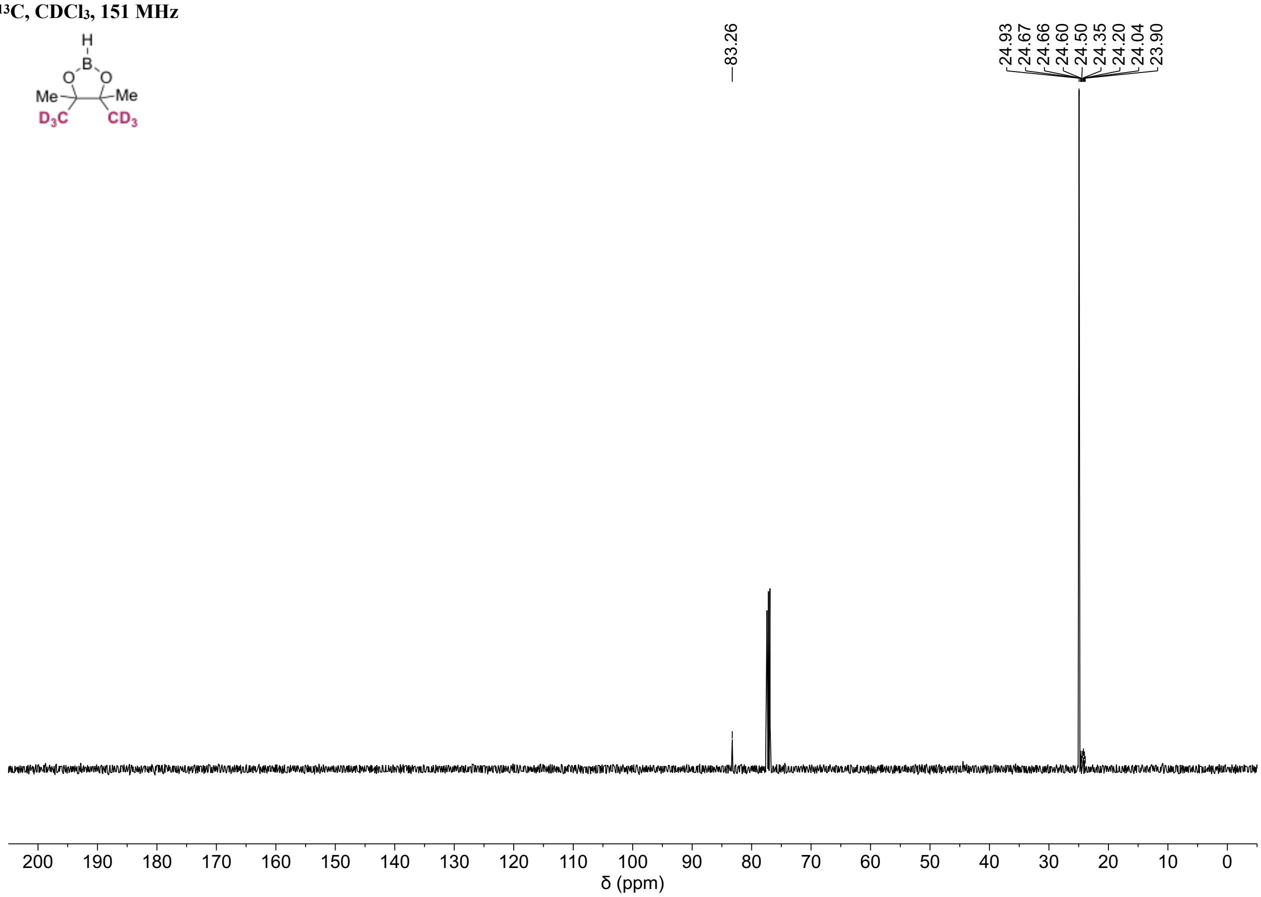

^{11}B, $CDCl_3$, 128 MHz

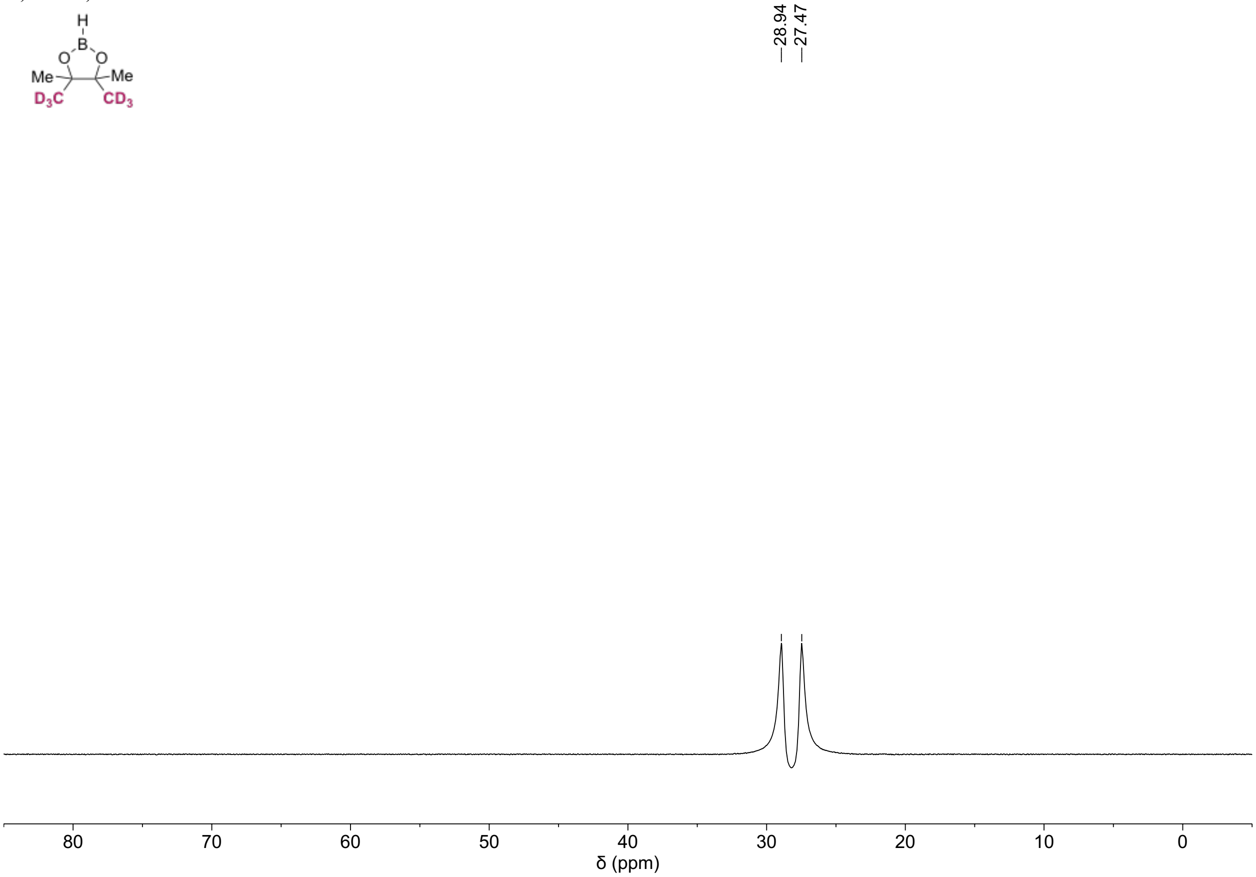

^{1}H, $CDCl_3$, 500 MHz

1

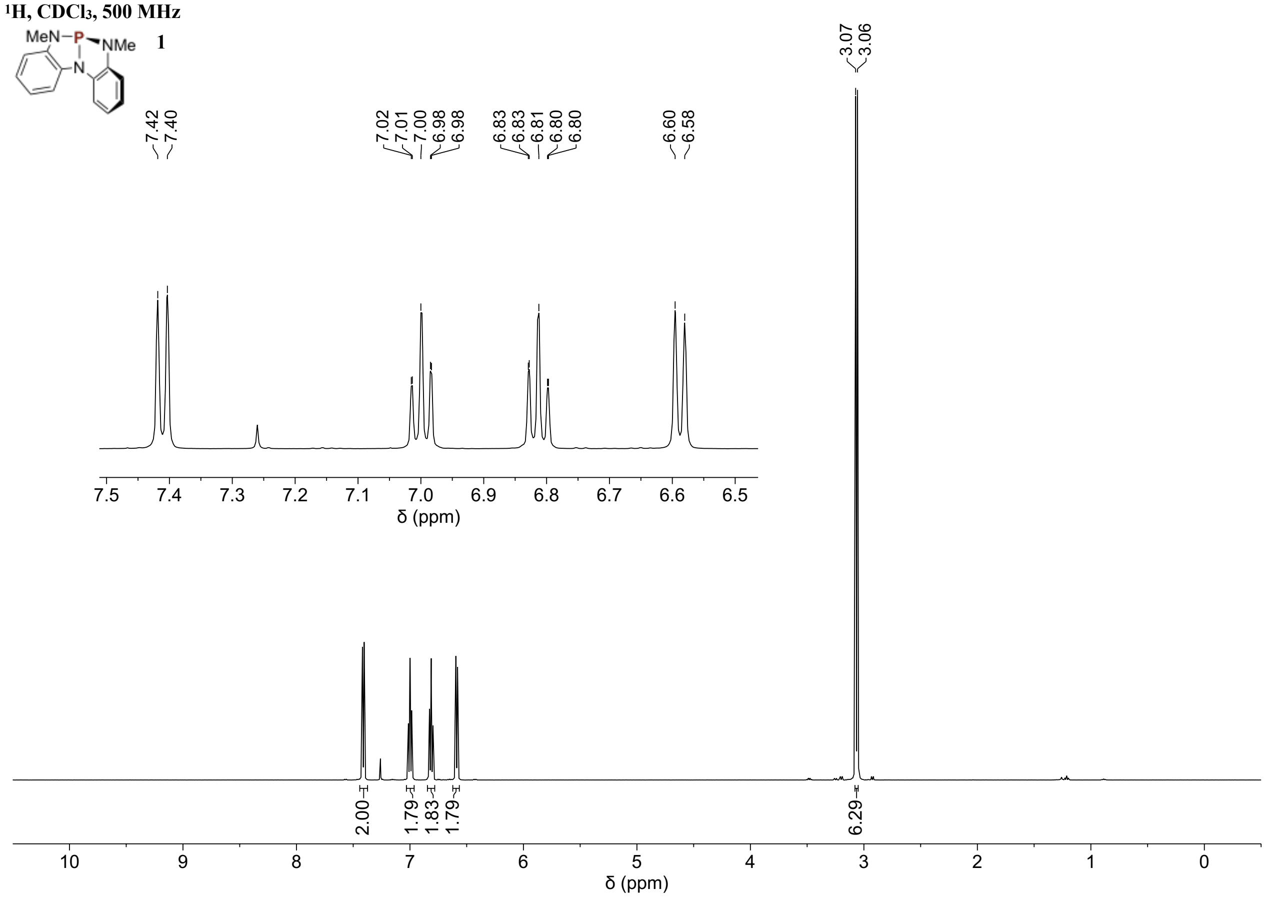

^{13}C, $CDCl_3$, 101 MHz

1

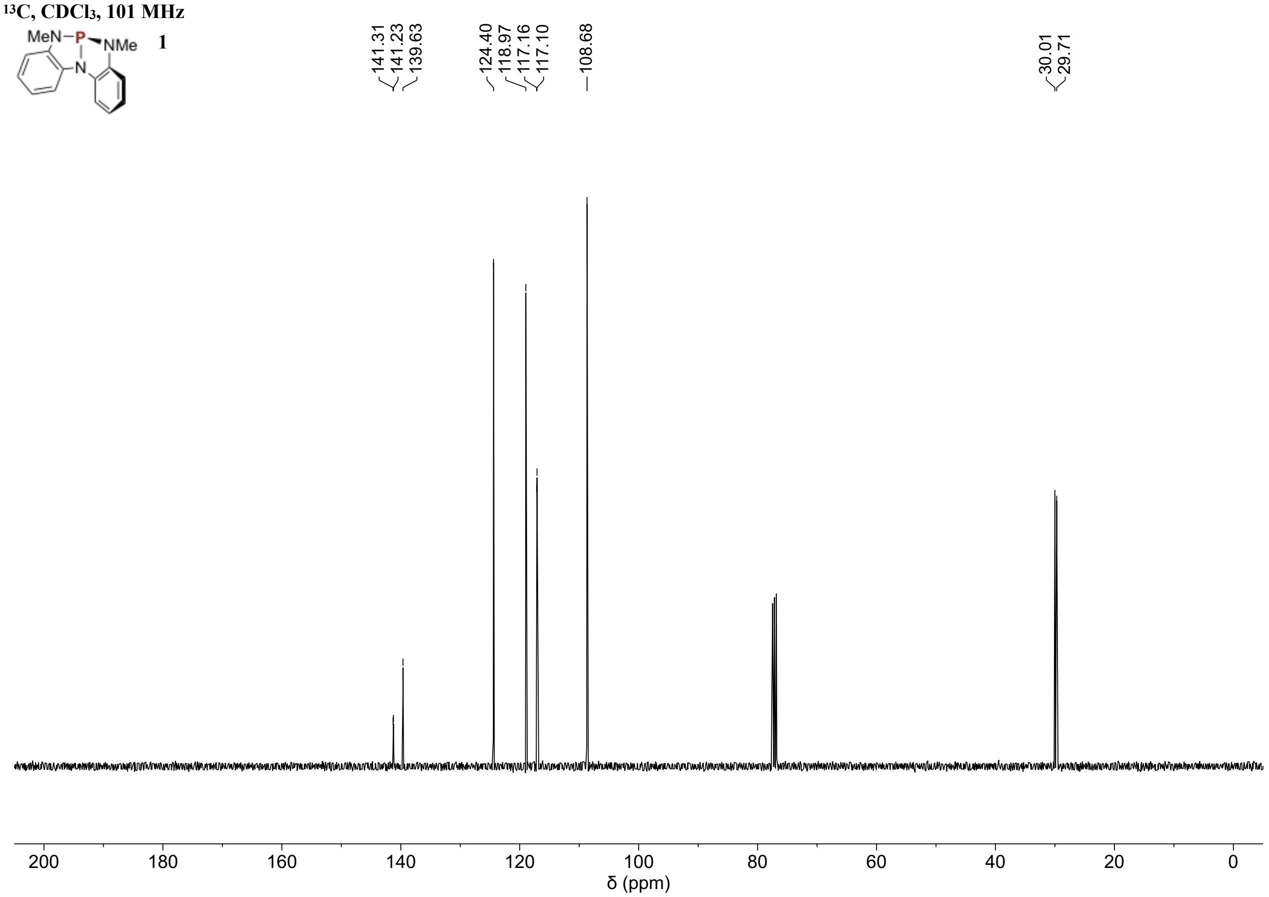

^{31}P, CDCl$_3$, 162 MHz

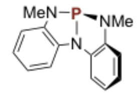

1

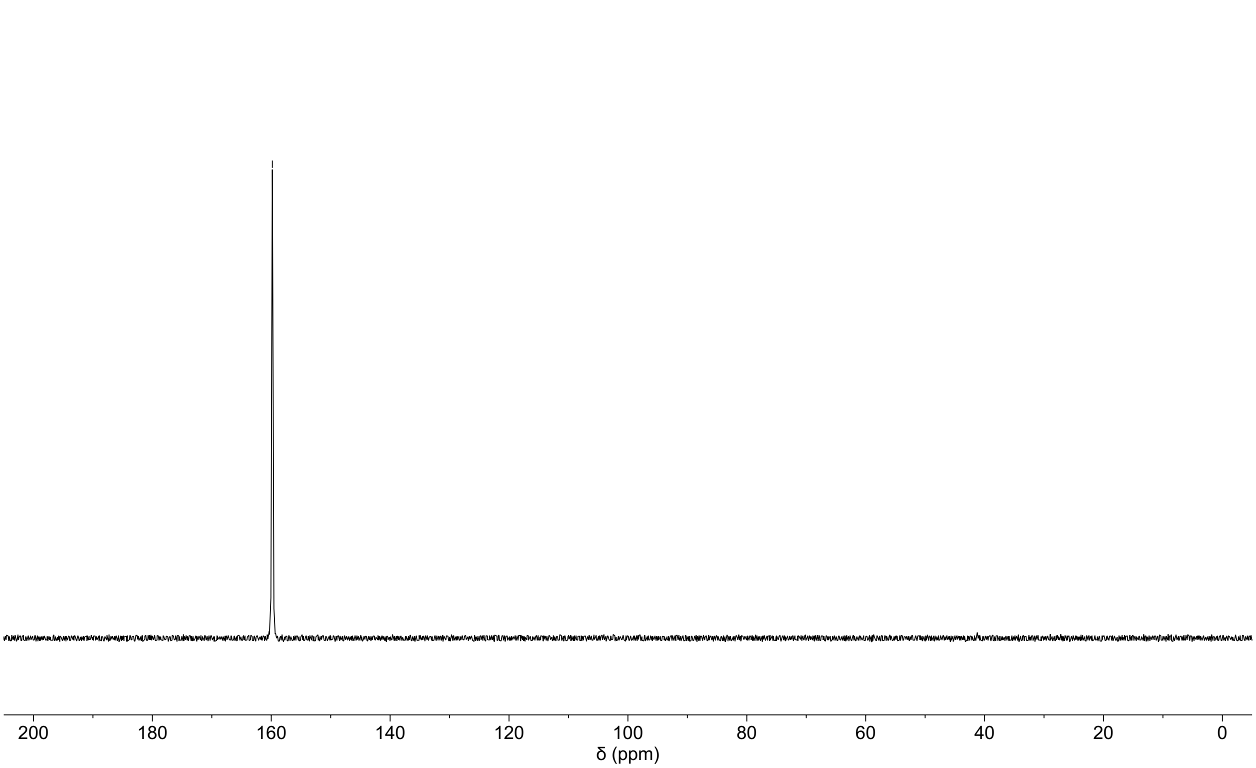

^{1}H, $CDCl_3$, 400 MHz

3

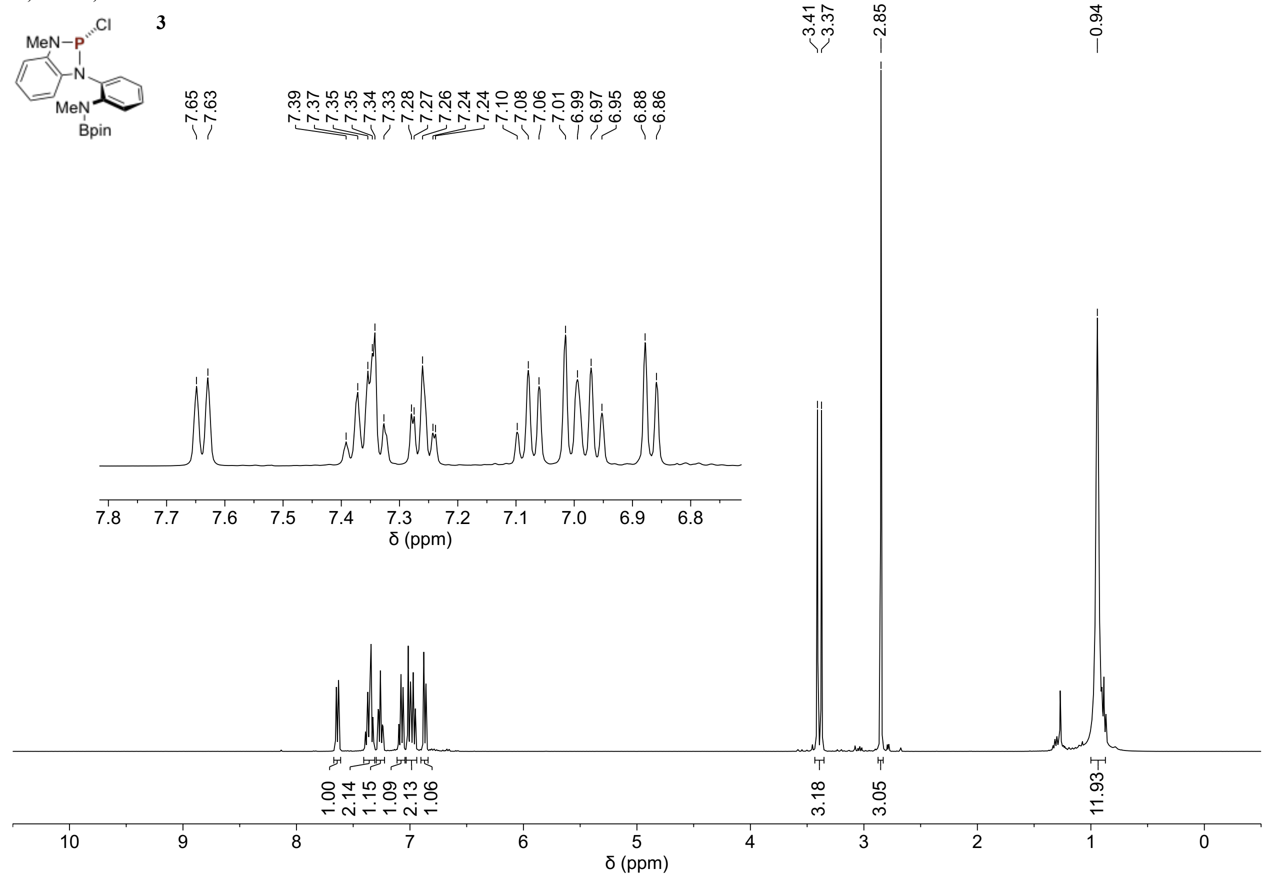

^{13}C, $CDCl_3$, 101 MHz

3

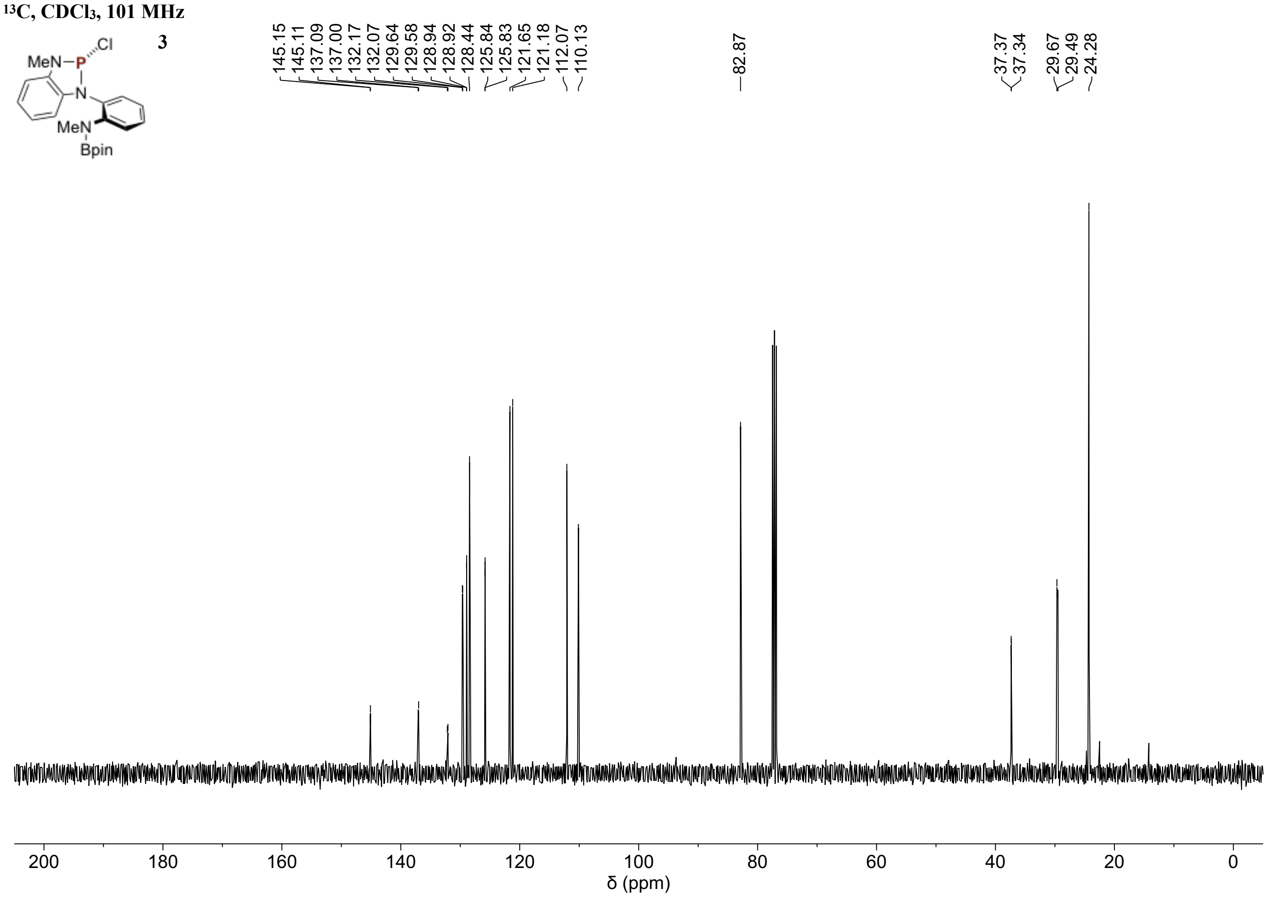

^{31}P, $CDCl_3$, 162 MHz

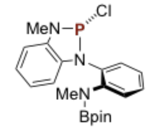

3

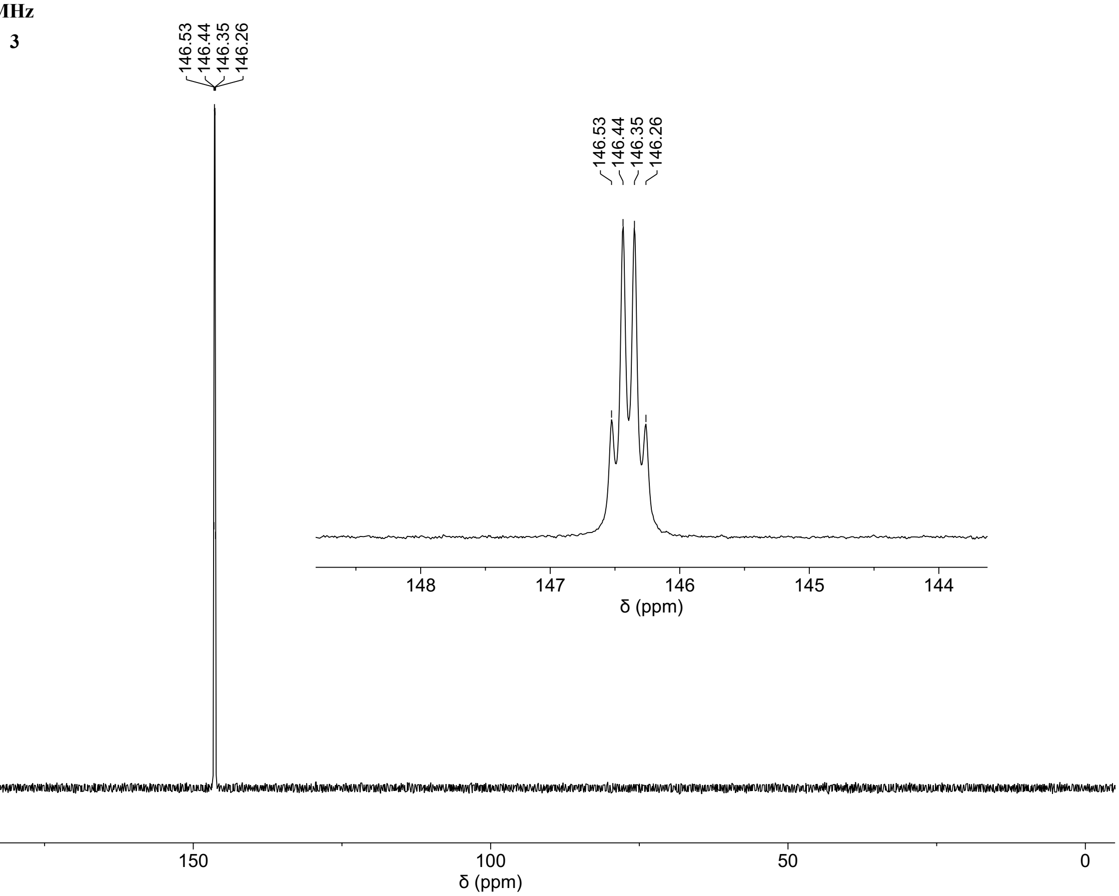

¹H, CDCl₃, 400 MHz

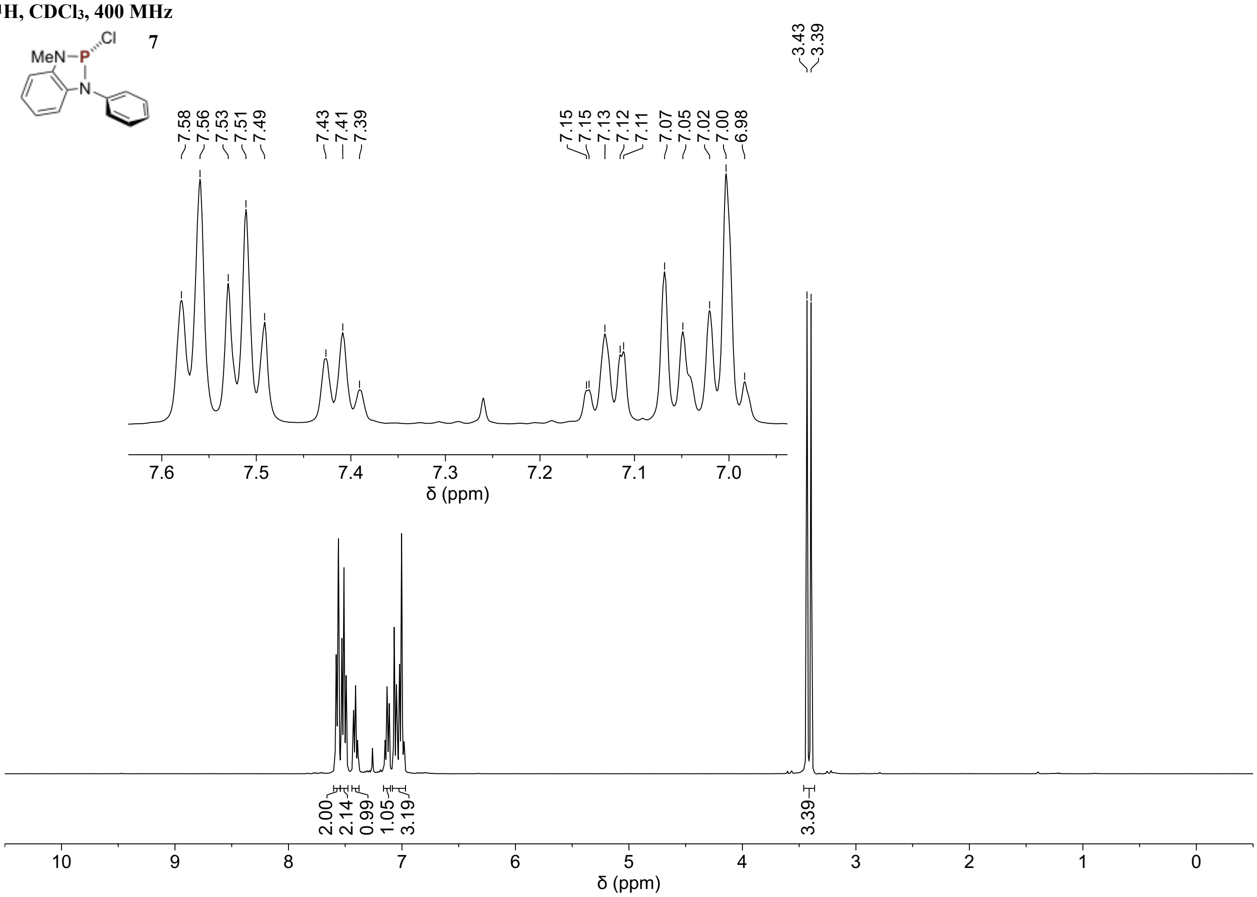

¹³C, CDCl₃, 101 MHz

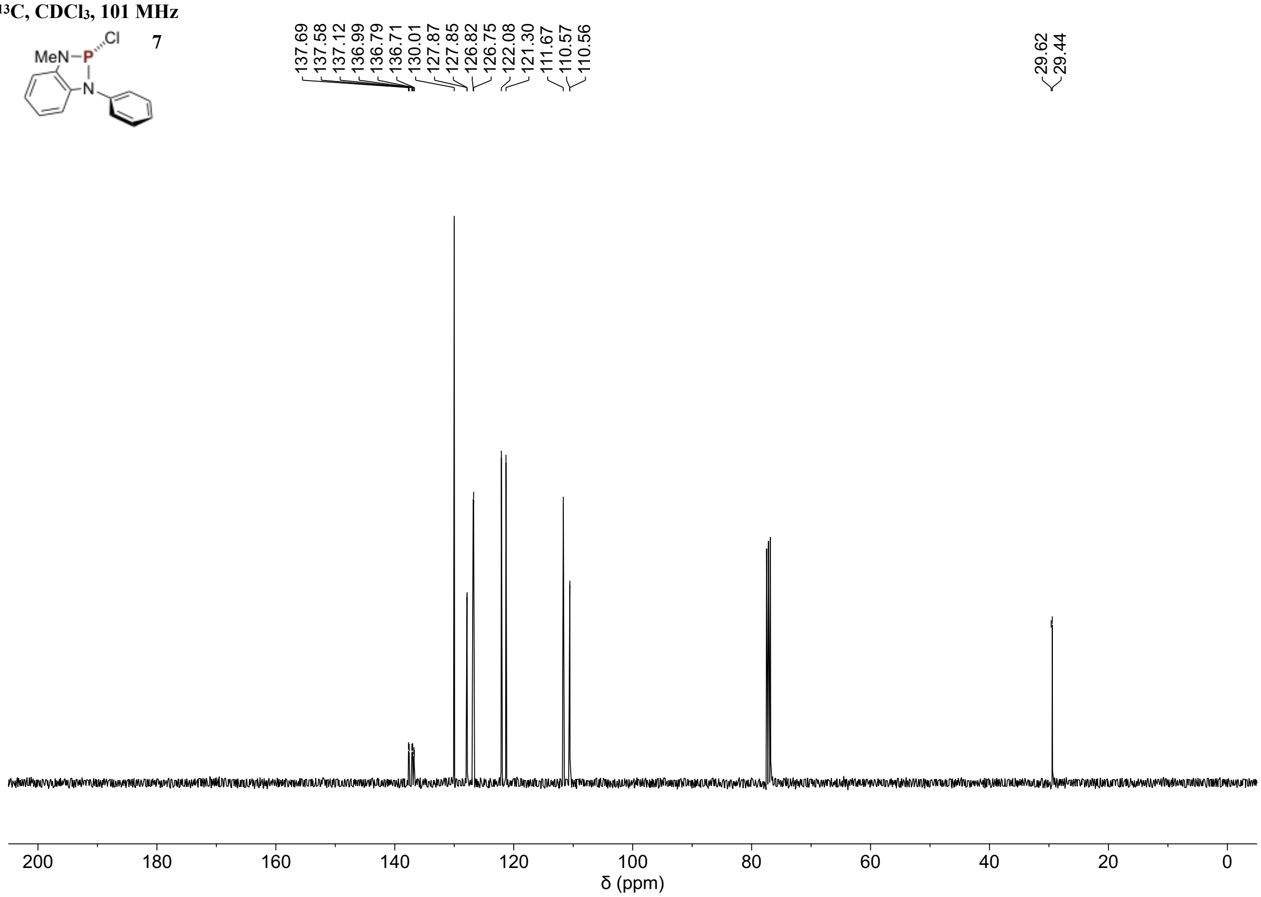

^{31}P, CDCl$_3$, 203 MHz

7

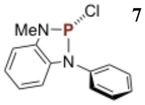

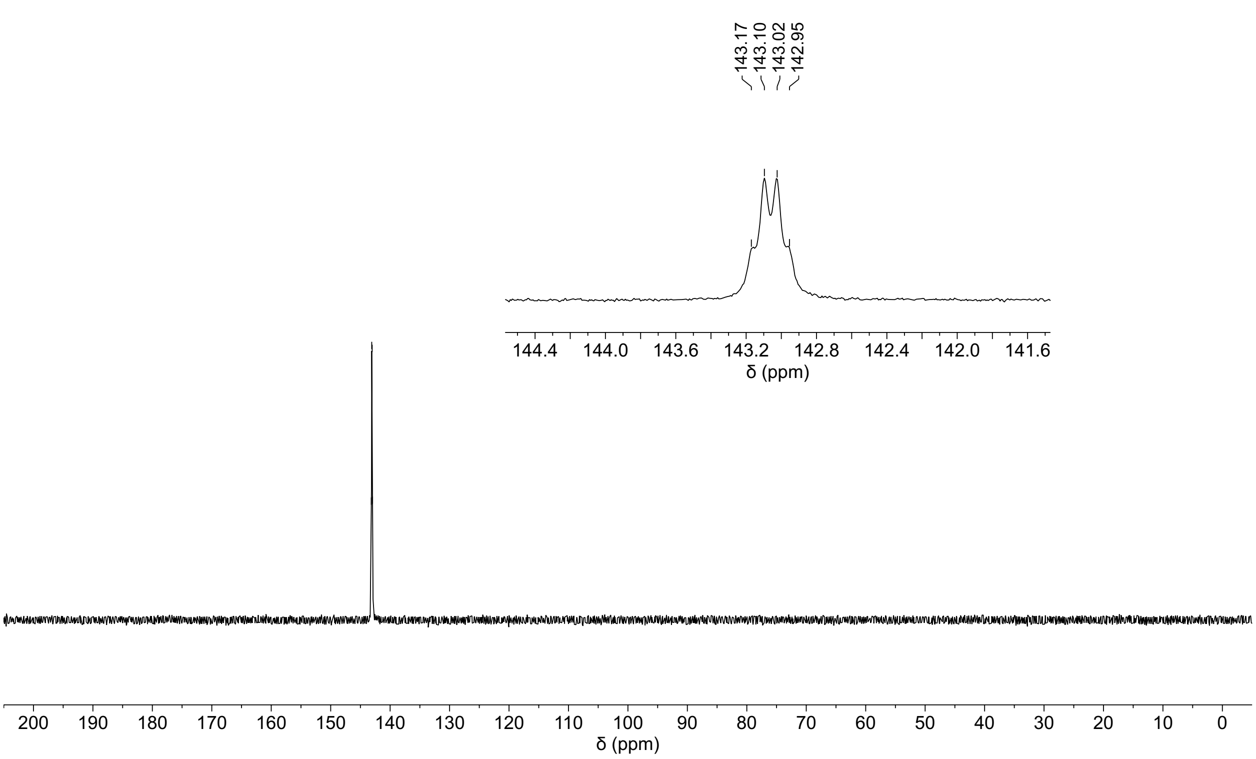

Supplement: SC-012-D0SC05620K-s001 [file SC-012-D0SC05620K-s001.pdf]
